# Polychaetes of Greece: an updated and annotated checklist

**DOI:** 10.3897/BDJ.5.e20997

**Published:** 2017-12-22

**Authors:** Sarah Faulwetter, Nomiki Simboura, Nikolaos Katsiaras, Giorgos Chatzigeorgiou, Christos Arvanitidis

**Affiliations:** 1 University of Patras, Department of Zoology, Section of Marine Biology, Patras, Greece; 2 Institute of Oceanography, Hellenic Centre for Marine Research, Anavyssos, Greece; 3 Department of Marine Sciences, University of the Aegean, Mytilini, Greece; 4 Institute of Marine Biology, Biotechnology and Aquaculture, Hellenic Centre for Marine Research, Heraklion Crete, Greece; 5 Hellenic Center for Marine Recearch (HCMR), Heraklion Crete, Greece; 6 Hellenic Centre for Marine Research, Heraklion, Greece

**Keywords:** Eastern Mediterranean, Polychaeta, Annelida, inventory, biodiversity, new records, replacement name

## Abstract

**Background:**

The last annotated checklist of marine polychaetes in Greece was published in 2001. Since then, global taxonomic progress, combined with many new species records for Greece, required a thorough review of the taxonomic, nomenclatural and biogeographic status of the national species list. This checklist revises the status of all extant polychaete species reported from the Greek Exclusive Economic Zone since 1832. The work was undertaken as part of the efforts on compiling a national species inventory (Greek Taxon Information System initiative) in the framework of the LifeWatchGreece Research Infrastructure.

**New information:**

This checklist comprises an updated and annotated inventory of polychaete species in Greek waters, compiled from literature reports, online databases, museum collections and unpublished datasets. The list provides information on 836 species-level taxa from Greece, of which 142 are considered questionable. An additional 84 species reported in the past are currently considered absent from Greece; reasons for the exclusion of each species are given. Fourteen species are reported here for the first time from Greek waters. At least 52 species in the present list constitute in fact a complex of cryptic or pseudo-cryptic species. Forty-seven species are considered non-native to the area. In addition to the species-level taxa reported in this checklist, eleven genera have been recorded from Greece with no representatives identified to species level. One replacement name is introduced. For each species, a comprehensive bibliographic list of occurrence records in Greece and the synonyms used in these publications are provided as supplementary material. Where necessary, the taxonomic, nomenclatural or biogeographic status is discussed. Finally, the findings are discussed in the wider context of Mediterranean polychaete biogeography, taxonomic practice and worldwide research progress.

## Introduction

### Polychaete research in Greece

The history of polychaete studies in Greece can be traced back to classical antiquity. Aristotle, although he tended to neglect polychaetes in his works on natural history (Voultsiadou et al. 2017) gave the first description of a “scolopendra thalassia” (referring to *Hermodice carunculata* Pallas, 1776): “*There are also marine scolopendras, similar to the terrestrial ones, but smaller in size; they live on the rocky substrates. Their colour is richer red and they bear more and much slender feet than the terrestrial scolopendras. They do not occur in great depths, as do the marine snakes*” (History of Animals, 4^th^ century B.C.). Modern systematic polychaete research in the country started in the 19^th^ century (e.g. Brullé 1832, Forbes 1842, von Marenzeller 1893). Since then, most research has been directed towards the study of macrobenthic coastal soft bottom polychaete communities in an ecological context. To date, the only taxonomic work studying exclusively planktonic polychaetes in Greece is by Wesenberg-Lund (1939a), based on samples taken during the Danish Oceanographic Expedition of the research vessel “Thor” from a few stations in Greek waters (Mavraki et al. 2016). Hard substrates, meiobenthic communities and deep sea habitats are understudied in Greece (e.g. Tselepides et al. 2004) while no terrestrial or fresh water polychaetes are known from the country. Specialised taxonomic or faunistic works from Greece are rare and the large body of ecological studies comprises the most important source for species records and thus for the creation of national checklists.

### Rationale for the checklist

The first polychaete checklists covering the Greek waters were those of Arvanitidis (2000a) for the Aegean Sea (including the Turkish Aegean and the Sea of Marmara) under the broader framework of the entire Mediterranean inventory and of Simboura and Nicolaidou (2001) for all Greek seas; both checklists included only benthic species. In the framework of the European Union project PESI (Pan-European Species directories Infrastructures), Faulwetter (2010) combined the above lists and additional sources into an updated checklist, but this was simply a computer-based compilation of existing sources without further verification or annotation of the records.

After 2001, marine research, including benthic surveys (carried out by the Hellenic Centre for Marine Research (HCMR) and several universities) was intensified. Many new species records – often not only new for Greece, but also for the Mediterranean Sea – resulting from these studies, as well as the worldwide progress of taxonomic knowledge, required a thorough review of the taxonomic, nomenclatural and biogeographic status of many polychaete species recorded from Greece.

The construction of the Greek Taxonomic Information System (Bailly et al. 2016), which forms the taxonomic backbone of the LifeWatchGreece Research Infrastructure, provided the framework for the present study: the compilation of a comprehensive, annotated and updated checklist of the Greek polychaete fauna, expanding and updating former works (i.e. Arvanitidis 2000a, Simboura and Nicolaidou 2001). In addition, this publication complements recent checklists from other areas of the Mediterranean and Black Sea (e.g. Çınar 2005, Castelli et al. 2008, Ayari et al. 2009, Zaâbi et al. 2009, Martín and Gil 2010, Zaâbi et al. 2012, Çınar et al. 2014, Mikac 2015) and thus serves as an information resource for future polychaete studies in this biogeographic region.

### Taxonomic scope

Several recent studies indicate the paraphyletic status of polychaetes (e.g. Rouse and Pleijel 2001, de Oliveira Almeida et al. 2003, Weigert and Bleidorn 2016) and the higher classification of Annelida has been in a constant flux over the last years (e.g. Zrzavý et al. 2001, Helm et al. 2012, Weigert and Bleidorn 2016). As the phylogenetic position of many groups within the Annelida is not yet resolved, we chose to restrict the "polychaetes" to their traditional definition in this checklist and include those clades covered by Rouse and Pleijel (2001). We anticipate this approach to be more helpful to the majority of users of this checklist than following the most recent phylogeny which would exclude certain "traditional" polychaete families (see Weigert and Bleidorn 2016).

### Geographic scope and characteristics of the region

This checklist includes all polychaete taxa reported from the waters of the Greek Exclusive Economic Zone (EEZ) (as delimited by the Marine Regions database, see also Bailly et al. 2016). It does not cover a biogeographic area but constitutes an inventory of the national polychaete fauna. Thus, species reported from neighbouring countries but not (yet) from Greece are not included in this list, despite the fact that they are highly likely to occur in Greek water too.

Greece is located centrally in the Eastern Mediterranean Sea and thus lies in one of the world's marine biodiversity hotspots (Bianchi and Morri 2000a, Coll et al. 2010). Its coastline stretches over 18,000 km, showing a variety of coastal landforms, from cliffs and rocky shores to beaches, lagoons and river deltas and encompassing around 9,800 islands and rocky islets (Issaris and Salomidi 2012). The Greek EEZ comprises parts of three biogeographically distinct water bodies: the Aegean Sea in the east, the Ionian Sea in the west and the Libyan Sea in the south. The Aegean Sea exchanges waters in the north with the Black Sea through the Bosphorus Strait, the Marmara Sea and the Strait of Dardanelles. Extensive faulting and vertical tectonic movements have resulted in the fracturing of the Hellenic mountain chains and the creation of deeply incised embayments and semi-enclosed basins, whereas active underwater volcanoes and shallow-water hydrothermal vents can be found in several places in the Aegean Sea (Dando et al. 1999; see also Sini et al. (2017) for a detailed overview of the ecology of the Aegean Sea). The island of Crete forms the southern border of the Aegean and separates it from the Libyan Sea. The Libyan Sea is characterised by deep sea features such as trenches, extensive deep sea plateaus, hydrothermal vents, cold seeps and mud volcanoes. It is part of the Levantine basin and linked to the Red Sea via the Suez canal which forms the point of entry for Lessepsian migrant species. In the west, the Ionian Sea communicates with the Western Mediterranean and the Adriatic Sea. The Greek part of the Ionian Sea (East Ionian Sea) is characterised by the existence of the deep Hellenic Trench with depths exceeding 5,000 m – the deepest part of the Mediterranean Sea. Primary production in the Mediterranean decreases towards the eastern basin and thus the Greek waters are highly oligotrophic (SoHelME 2005), characterised by a limited availability of nutrients, primarily phosphorus (Thingstad et al. 2005). However, within this oligotrophic environment relatively eutrophic areas do exist (e.g. the North-West Aegean is highly productive in terms of nutrients and biological production compared to the Ionian, the South Aegean and the Levantine basin (e.g. Lykousis et al. 2002)). Knowledge on the polychaete fauna in each of these regional seas is unequally distributed, as the overwhelming majority of benthic research (around 80% of studies) has been performed in the Aegean Sea, whereas the deep sea habitats characterising the Libyan Sea are almost completely unexplored.

## Materials and methods

### Literature resources

**Figure 1.**
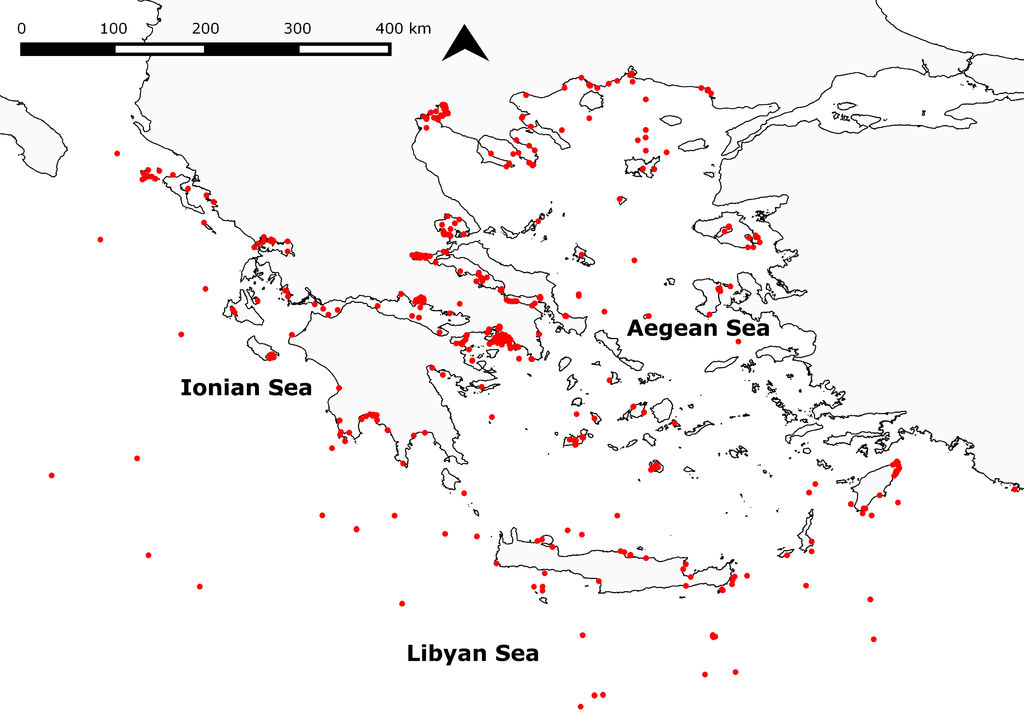
Sampling stations from which polychaetes were recorded, compiled from literature sources and databases which where not included in previous checklists.

Existing checklists on Greek polychaetes were combined, complemented, verified and annotated with information on species occurrences in Greece, using over 260 bibliographic and online resources, covering almost 200 years of research (1832–2017). A comprehensive list of all references from Greece for each species is contained in Suppl. material 2. These sources include publications from scientific journals, book chapters, theses, conference proceedings and biogeographic and museum collection databases. In addition, unpublished records generated in the framework of research projects conducted within the Hellenic Centre of Marine Research (HCMR) after 2001 were used to assess the occurrence status of many controversial and non-native species (Suppl. material 1). We deliberately chose to include grey literature and database records, as many of these have been used in secondary works and we prefer to draw attention to doubtful records rather than ignoring them. The additions to the previous checklists comprise polychaete records from 486 sampling stations which were compiled from a) literature and data sources from after 2001 and b) literature and data sources from before 2001 if these had not been covered by previous checklists (Fig. 1). The majority of these stations were monitored regularly or occasionally. Samples were mainly collected from shallow soft-bottom habitats, though a few samples from pelagic, deep-sea and hard substrate habitats are also included.

The final checklist was submitted to the World Register of Marine Species and the Pan-European Species directories Infrastructure to replace the latest version by Faulwetter (2010).

### How to read this checklist

The nomenclature used here follows the World Register of Marine Species (WoRMS) and the most recent taxonomic literature. Taxa are arranged alphabetically in hierarchical order (genera and species within families); suprafamiliar classifications are omitted. The inclusion of species follows a conservative approach: species with a questionable presence in Greek waters are included in the list and clearly indicated as such; we excluded only species that are either considered taxonomically indeterminable according to the most recent taxonomic works or where Greek records were shown to be misidentifications. These are presented separately at the end of the manuscript.

For each species, one or more of the following types of information may be provided:

**1. Nomenclature**

*If no information is provided in the "Nomenclature" field*: Species has only been recorded under its currently accepted name.

*If information is provided in the "Nomenclature" field:* Not a comprehensive list of synonyms (for this, the reader is advised to consult WoRMS), but all names under which the species has been reported from Greece (respective literature resources for each name in Suppl. material 2). Subjective and objective synonyms (subjective: two different type specimens exist and original descriptions exist, synonymy is based on expert opinion; objective: different names exist as a result of assigning a species to a different genus or rank but are based on the same type specimen) as well as different rank assignments (e.g. subgeneric vs. generic level) are listed. Misspellings and spelling variations (e.g. species epithets ending in -a vs. -us) are excluded. Misidentifications (i.e. specific specimens which were in the past identified as belonging to another species but then re-examined) are also omitted to avoid confusion; instead, a remark is given in the Notes field and the record is annotated in Suppl. material 2.

**2. Native Status**

*If no information is provided in the "Native status" field*: Species is considered native to the Mediterranean.

*If information is provided in the "Native status" field*: Contains information on the native status of the species in Greece, following the terminology and definitions by Zenetos et al. (2011):

- ***non-native (established)***: Species with at least two independent published records from Greece, separated in time and space;
- ***non-native (casual)***: Reported only once in the scientific or grey literature from Greece;
- ***non-native (questionable)***: Non-native species reported from Greece whose presence in the country is questionable and needs to be confirmed by an expert, or which have an unresolved taxonomic status. In this paper, doubtful records of species with an alien origin are not indicated as non-native if they have never been reported before as alien from the Mediterranean, whereas species previously reported as alien from the Mediterranean Sea but shown here to be questionable are retained as "non-native (questionable)" to highlight their status for further assessment by experts;
- ***cryptogenic***: Species of unknown origin that could be either native or introduced, clear evidence for either origin being absent.

**3. Notes**

*If no information is provided in the "Notes" field*: Species has an uncontroversial taxonomic and biogeographic status and regular records from Greece and the Mediterranean exist. A full reference list of all reports from Greece for each species can be found in Suppl. material 2.

*If information is provided in the "Notes" field:* May contain information on:

- the taxonomic history, nomenclatural or occurrence status where these are deemed useful for assessing the taxonomic or occurrence status of the species in Greece.
- literature references reporting the species from Greece and additional information on the species' distribution: only in cases where the species has been reported fewer than three times from Greece (excluding checklists and other secondary works).
- whether the species has originally been described from the Mediterranean.

*Distribution information*:

Where statements are made on the occurrence (or absence) of the species in other areas of the Mediterranean, this information was derived from a combined assessment of:

- the checklist of Mediterranean polychaetes by Martín and Gil (2010);
- recent regional checklists of polychaetes in the Mediterranean (e.g. Çınar 2005, Castelli et al. 2008, Ayari et al. 2009, Zaâbi et al. 2009, Çınar et al. 2014, Mikac 2015);
- extensive unpublished notes by C. Arvanitidis covering the literature on Mediterranean polychaetes until ca. 1994;
- literature research.

Information on species distributions outside the Mediterranean are derived from a combined assessment of literature sources and distribution records in the Ocean Biogeographic Information System (OBIS) (unless a specific source is cited). Distribution information is not intended to list all known distributions of the species but rather to provide an estimate of the species’ likelihood to occur in the Mediterranean.

*Questionable species*:

Species are indicated as "questionable" if one or more of the following criteria are met:

- the species concept is not clearly defined or outdated and in need of revision, rendering literature records highly uncertain;
- species is indicated as possibly absent from the Mediterranean in a recent taxonomic work (preferably a review) based on examination of Mediterranean specimens;
- species is in the Mediterranean reported exclusively (or almost exclusively) from Greece and has otherwise a distinctly different distribution range;
- species has been reported only once in grey literature before 1993 (chosen as a cut-off date, as thorough investigations of the Greek polychaete fauna followed afterwards)

Species reported as "cf." in primary records are not indicated as questionable, as the modifier "cf." already implies uncertainties in the identification and often a possible deviation from the nominal species.

*Species complexes*:

Species known to constitute a complex of cryptic (morphologically indistinguishable but genetically different) or pseudo-cryptic (morphologically very similar) species are here treated as sensu lato, following *Nygren and Pleijel (2011).* Thus, clarifications pending through molecular analyses, we assume the presence of at least one species of the complex in Greece. The keyword "Species complex" in the Notes field indicates that the name has been applied to a species complex as defined above.

## Checklists

### Acoetidae Kinberg, 1856

#### Eupanthalis kinbergi

McIntosh, 1876

##### Notes

Many records of *Eupanthalis kinbergi* in the Mediterranean actually belong to *Euarche tubifex* Ehlers, 1887, including the description by Fauvel (1923) (Pettibone 1989). Arvanitidis (2000a) reports both *Euarche tubifex* and *Eupanthalis kinbergi* in his checklist of Aegean polychaetes, re-assigning all records of *Eupanthalis kinbergi* that were probably identified using the key by Fauvel (1923) to *Euarche tubifex*, but no primary records of *Euarche tubifex* from Greece exist.

#### Panthalis oerstedii

Kinberg, 1856

#### Polyodontes frons

Hartman, 1939

##### Notes

Questionable status. In the Mediterranean only reported from Greece (Bogdanos and Satsmadjis 1983, Bogdanos and Satsmadjis 1987, NCMR 1989). Commonly distributed in the Greater Caribbean Region.

#### Polyodontes maxillosus

(Ranzani, 1817)

##### Notes

Type locality: Mediterranean.

### Acrocirridae Banse, 1969

#### Acrocirrus frontifilis

(Grube, 1860)

##### Notes

Type locality: Mediterranean (Croatia).

#### Flabelligena mediterranea

(Kolmer, 1985)

Flabelligella mediterranea Kolmer, 1985

##### Notes

Originally described from the Hellenic Trench (Matapan Deep; 35°49'48''N, 22°20'42''E; 4690 m depth), no other records from Greece.

#### Macrochaeta clavicornis

(M. Sars, 1835)

### Ampharetidae Malmgren, 1866

#### Adercodon pleijeli

Mackie, 1994

##### Notes

Reported from Greece by Simboura and Zenetos (2005). Type locality: Mediterranean (Banyuls-sur-Mer, France).

#### Alkmaria romijni

Horst, 1919

##### Notes

Questionable status. In the Mediterranean, only reported from Greece (Kisseleva 1961). Record doubted by Arvanitidis (1994) and Çınar et al. (2014) based on the species' ecology: *Alkmaria romijni* is typically a brackish water species but has been reported from a depth of 65–100 m in the open waters of the Aegean Sea.

#### Amage adspersa

(Grube, 1863)

##### Notes

Type locality: Mediterranean (Adriatic).

#### Ampharete acutifrons

(Grube, 1860)

##### Notes

Questionable status. Mediterranean records of *Ampharete acutifrons* in the Mediterranean are doubted by several authors (e.g. Parapar et al. 1993a, Mikac 2015) based on the species' boreal distribution. Specimens of *Ampharete acutifrons* from the Adriatic were shown to belong to *Ampharete lindstroemi* Hessle, 1917 (Mikac 2015), thus *Ampharete acutifron*s may actually be absent from the region. Jirkov (2001) considers *Ampharete acutifrons* to probably constitute a species complex, based on observed differences between Atlantic and Pacific species.

#### Ampharete grubei

Malmgren, 1865

##### Notes

*Ampharete grubei* had long been considered a junior synonym of *Ampharete acutifrons* (Grube, 1860) and was reinstated by Jirkov (2001). Primary literature records from Greece under the name *Ampharete grubei* exist but were subsequently reported by Arvanitidis (2000a) and Simboura and Nicolaidou (2001) as *Ampharete acutifrons*.

#### Ampharete octocirrata

(Sars, 1835)

Sabellides octocirrata (M. Sars, 1835)

#### Amphicteis gunneri

(M. Sars, 1835)

#### Anobothrus gracilis

(Malmgren, 1866)

##### Notes

Reported from worldwide locations but probably restricted to circumpolar areas; specimens from other regions may belong to other species (Jirkov 2008).

#### Auchenoplax crinita

Ehlers, 1887

#### Lysippe labiata

Malmgren, 1866

#### Melinna monoceroides

Fauvel, 1936

#### Melinna palmata

Grube, 1870

#### Neosabellides oceanica

(Fauvel, 1909)

##### Notes

Questionable status. Few records from the Mediterranean outside Greece (e.g. Spain: Nebra et al. 2011; France: Guérin and Massé 1976) otherwise known from the European Atlantic coasts. Arvanitidis (1994) regards its presence in the Mediterranean as doubtful.

#### Sosane sulcata

Malmgren, 1866

#### Uschakovius enigmaticus

Laubier, 1973

##### Notes

Originally described from the Hellenic Trench (Matapan Deep; 36°01'48''N, 22°24'36''E; 3174 m depth), no other records from Greece. Considered endemic to the type locality (Paterson et al. 2009).

### Amphinomidae Lamarck, 1818

#### Chloeia candida

Kinberg, 1857

Chloeia viridis Schmarda, 1861

##### Notes

Reported from Greece by Arvanitidis (1994). In the Mediterranean also reported from the western basin (Fauvel and Rullier 1959), otherwise distributed in the Caribbean and tropical Atlantic. *Chloeia viridis* is the name prevailing in literature, with *Chloeia candida* as a synonym (referred to synonymy by Hartman (1948), although Barroso and Paiva (2011) stress that this synonymy should be re-evaluated). The publication year of Kinberg's species is sometimes cited as 1910, but the species was in fact described in 1857 (Kinberg 1857) and thus the name *Chloeia candida* takes priority.

#### Chloeia venusta

Quatrefages, 1866

##### Notes

Type locality: Mediterranean (Sicily).

#### Chloenopsis atlantica

(McIntosh, 1885)

##### Notes

Böggemann (2009) examined the syntypes of *Chloenopsis atlantica* and postulated a possible synonymy of *Chloenopsis atlantica* with *Bathychloeia sibogae* Horst, 1910. However, Kudenov and Borda (2013), having studied the types of the monotypic genera *Chloenopsis* and *Bathychloeia*, consider both genera distinct.

#### Eurythoe complanata

(Pallas, 1766)

##### Ecological interactions

###### Native status

Non-native (casual)

##### Notes

Species complex. Reported from Greece by Chatzigeorgiou et al. (2016) based on a single specimen (identified using Day (1967) and Barroso et al. (2010)). *Eurythoe complanata* is a complex of cryptic and pseudo-cryptic species (Barroso et al. 2010, Arias et al. 2013a), containing two cryptic forms of *Eurythoe complanata* and the species *Eurythoe laevisetis* Fauvel, 1914, which differs from *Eurythoe complanata* sensu stricto by the absence of harpoon-shaped notochaetae (Arias et al. 2013a). In the Mediterranean, two of the species in this species complex occur: *Eurythoe laevisetis* in the central and western basin, and *Eurythoe complanata* in the eastern basin and the Alboran Sea. Both species are native to the Caribbean and tropical Atlantic and non-native to the Mediterranean, but their exact pathways of introduction to the Mediterranean and thus the identity of the species occurring in the eastern basin are yet unknown (Arias et al. 2013a).

#### Hermodice carunculata

(Pallas, 1766)

Amphinome savignyi Brullé, 1832 | *Hermodice carunculata* (Pallas, 1766)

##### Notes

The identity of the circumtropical species *Hermodice carunculata* has been the subject of several investigations during the last years. Yáñez-Rivera and Salazar-Vallejo (2011), based on morphological investigations of fixed and living specimens, resurrected *Hermodice nigrolineata* Baird, 1868 for the Eastern Atlantic and Mediterranean populations of the species. Ahrens et al. (2013) found no genetic differences between *Hermodice carunculata* populations of the Caribbean and the Eastern Atlantic and Mediterranean and placed *Hermodice nigrolineata* again into synonymy with *Hermodice carunculata*. The findings of Chatzigeorgiou et al. (2014) concerning the phylogenetic relationships of the eastern Mediterranean populations of *Hermodice carunculata* support the results of Ahrens et al. (2013).

#### Linopherus canariensis

Langerhans, 1881

##### Ecological interactions

###### Native status

Non-native (casual).

##### Notes

Reported from Greece by Chatzigeorgiou et al. (2016). Atlantic species, considered alien to the Mediterranean (Çınar 2009). Known from Turkey and Cyprus (Çınar 2009), Italy (Cosentino and Giacobbe 2010) and Egypt (Dorgham et al. 2014).

#### Notopygos megalops

McIntosh, 1885

##### Notes

Questionable status. The Greek records of *Notopygos megalops* probably belong to a different species. *Notopygos megalops* was originally described based on a juvenile specimen from the Caribbean Sea and used to be considered a synonym of *Notopygos crinita* (Grube, 1855) before it was re-described by Yáñez-Rivera and Carrera-Parra (2012) from the Caribbean Sea based on adult characters. This re-description of *Notopygos megalops* differs from the description by Fauvel (1923) (which was probably used for the identification of the Greek specimens) in the following characters: Branchiae from chaetiger 6 (Yáñez-Rivera and Carrera-Parra 2012) instead of chaetiger 7 (Fauvel 1923); anterior branchiae with main stem and seven branchial filaments, in median chaetigers branching into four stems with five to seven filaments each (Yáñez-Rivera and Carrera-Parra 2012) vs. small tufts of 2–4 filaments anteriorly and only a single papilliform filament anteriorly Fauvel (1923). Yáñez-Rivera and Carrera-Parra (2012) define the start of the branchiae on chaetiger 6 as characteristic for the species; this seems to be invariable in juvenile individuals, too (McIntosh 1895). Thus, the species described by Fauvel (1923) and any specimens identified based on his description probably belong to a different species.

#### Pareurythoe borealis

(M. Sars, 1862)

##### Notes

Questionable status. Reported from Greece by Kisseleva (1983). Distributed in the boreal Atlantic, in the Mediterranean reported from the western basin (e.g. Jacquotte 1963, Bellan 1964a). The species' presence in Greece is doubtful, as it has not been reported from the Mediterranean for over three decades and is usually distributed in colder waters.

### Aphroditidae Malmgren, 1867

#### Aphrodita aculeata

Linnaeus, 1758

##### Notes

Specimens of *Aphrodita aculeata* from the Adriatic and Aegean may have been confused with *Aphrodita alta* Kinberg, 1865 or *Aphrodita perarmata* Roule, 1898 (Barnich and Fiege 2000b). In addition, the description of *Aphrodita aculeata* by Fauvel (1923) contains elements of both *Aphrodita aculeata* and *Aphrodita alta*, thus specimens identified using Fauvel's key may belong to either species.

#### Aphrodita perarmata

Roule, 1898

##### Notes

Questionable status. Reported from Greece by Marinov (1959), otherwise only known from the type locality in the North-East Atlantic. *Aphrodita perarmata, Aphrodita aculeata* Linnaeus, 1758 and *Aphrodita alta* Kinberg, 1865 are easily confused and all records require verification (Barnich and Fiege 2000b).

#### Laetmonice filicornis

Kinberg, 1856

##### Notes

Reported from Greece by Arvanitidis (2000a) based on a single specimen; in the Mediterranean also known from France (Bellan 1964a), Spain (Campoy 1982), Cyprus (Çınar 2005), Italy (Castelli et al. 2008) and the Adriatic (Mikac 2015), otherwise distributed in the North Atlantic.

#### Laetmonice hystrix

(Savigny in Lamarck, 1818)

Hermione hystrix (Savigny in Lamarck, 1818) | *Laetmonice hystrix* (Savigny in Lamarck, 1818)

##### Notes

Type locality: Mediterranean.

#### Pontogenia chrysocoma

(Baird, 1865)

##### Notes

Type locality: Mediterranean.

### Arenicolidae Johnston, 1835

#### Abarenicola claparedi

(Levinsen, 1884)

Abarenicola claparedi (Levinsen, 1884) | *Arenicola claparedei* Levinsen, 1884

##### Notes

Type locality: Mediterranean.

#### Arenicola marina

(Linnaeus, 1758)

##### Notes

Questionable status. Reported from Greece by Antoniadou et al. (2006) from rocky shores. The record is considered questionable on the basis of habitat and geographic distribution (the species occurs in tidal mudflats of the European Atlantic coast and North Sea).

#### Branchiomaldane vincenti

Langerhans, 1881

##### Notes

Reported from Greece by Arvanitidis (1994) and Chatzigeorgiou et al. (2016). In the Mediterranean also known from Israel (Ben-Eliahu 1976b), Tunisia (Cantone et al. 1978), Spain (Alós 1990), Italy (Castelli et al. 2008), Egypt (Abd-Elnaby 2009), Turkey (Çınar et al. 2014) and the Adriatic (Mikac 2015), otherwise distributed in the East Atlantic (Gravina 1991).

### Asteriomyzostomidae Jägersten, 1940

#### Asteriomyzostomum asteriae

(Marenzeller, 1895)

Myzostoma asteriae Marenzeller, 1895

##### Notes

Originally described from Greece as parasitic on the starfish species *Sclerasterias richardi* (Perrier in Milne-Edwards 1882) and *Sclerasterias neglecta* (Perrier, 1891) collected near Santorini, Kythira and Samos; no other records from Greece. Almost nothing is known of the species' distribution; the extensive description of its anatomy by Stummer-Traunfels (1903) is based on specimens provided by Marenzeller, presumably from the same collection as the type material.

### Capitellidae Grube, 1862

#### Capitella capitata

(Fabricius, 1780)

Capitella capitata (Fabricius, 1780) | *Capitella capitata capitata* (Fabricius, 1780)

##### Notes

Questionable status. Species complex. Several sibling species have been separated from *Capitella capitata* sensu lato worldwide since Grassle and Grassle (1976) showed it to constitute a complex of species. Blake (2009) restricts *Capitella capitata* sensu stricto to Arctic areas and considers Mediterranean specimens, including the description by Fauvel (1927), to show significant differences to *Capitella capitata* and thus to belong to other species. In Greece, there is evidence so far of at least another sibling species population (Gamenick et al. 1998). However, because of the high morphological similarity of the various sibling species, they cannot be reliably distinguished in routine investigations (Blake 2000). See also notes under *Capitella teleta* Blake, Grassle & Eckelbarger, 2009.

#### Capitella giardi

(Mesnil, 1897)

Capitella giardi (Mesnil, 1897) | *Capitellides giardi* Mesnil, 1897

#### Capitella minima

Langerhans, 1881

Capitella minima Langerhans, 1881 | *Capitomastus minimus* (Langerhans, 1881)

#### Capitella teleta

Blake, Grassle & Eckelbarger, 2009

##### Notes

Reported from Greece by Maidanou et al. (2017). *Capitella teleta* is one of the sibling species which used to be referred to as *Capitella* sp. I in laboratory and genetic studies (Blake et al. 2009). In the Mediterranean also known from France (Blake et al. 2009) and Turkey (Çınar et al. 2017). Present in the adjacent Sea of Marmara (Çinar et al. 2015) and Black Sea (Kurt-Şahin et al. 2017). Otherwise distributed in the North Atlantic and Pacific Ocean.

#### Capitellethus dispar

(Ehlers, 1907)

##### Notes

Questionable status. Listed by Arvanitidis (2000a), subsequently included in the checklist by Simboura and Nicolaidou (2001) but in fact based on a record from the Turkish Aegean, which is considered questionable by Çınar et al. (2014). The actual first record of the species from Greece is by Akoumianaki (2004). However, this species can be confused with several species of *Notomastus* Sars, 1851, based only on the thoracic chaetal formula which was traditionally used for identification. Green (2002) clarified some inaccuracies in previous descriptions of *Capitellethus* and used the teeth pattern in hooks as a reliable differentiating character between these two genera. If the presence of this species in Greece can be confirmed, it should be regarded as a non-native species. Originally described from New Zealand.

#### Dasybranchus caducus

(Grube, 1846)

##### Notes

Type locality: Mediterranean.

#### Dasybranchus gajolae

Eisig, 1887

##### Notes

Type locality: Mediterranean (Gulf of Naples).

#### Decamastus gracilis

Hartman, 1963

##### Notes

Questionable status. In the Mediterranean only reported from Greece (Akoumianaki 2004). Distributed in the Eastern Pacific, the Greater Caribbean and questionably in the Red Sea (Wehe and Fiege 2002).

#### Heteromastus filiformis

(Claparède, 1864)

##### Notes

Frequently reported from Greece and other parts of the Mediterranean; type locality: Mediterranean (Port-Vendres, France). Green (2002) clarified confusions that had arisen from typographic errors in the genus designation made by Hutchings and Rainer (1981). However, a number of problems concerning the correct identification of the species still exist. Juvenile specimens can be confused with adults of *Mediomastus* species, since chaetigers four and five initially carry hooks which are replaced by capillaries with age (Capaccioni-Azzati and El-Haddad 2015). In addition, different opinions regarding the presence and location of abdominal gills exist in literature (Capaccioni-Azzati and El-Haddad 2015).

#### Leiocapitella dollfusi

(Fauvel, 1936)

Leiocapitella dollfusi (Fauvel, 1936) | *Leiocapitella glabra* Hartman, 1947

##### Notes

Bellan (1964a) proposed the synonymy of *Leiocapitella glabra* (type locality California) with *Leiocapitella dollfusi* (originally described from the Moroccan coasts) based on the overlapping variability in the chaetal formula of the two species. Ben-Eliahu and Fiege (1995), probably unaware of Bellan (1964a), proposed a possible synonymy of *Leiocapitella dollfusi* with *Leiocapitella glabra* on the basis of the identical hook dentition, in addition to the chaetal formula (Capaccioni-Azzati and El-Haddad 2015). However, it is noteworthy that the presence of branchiae in posterior segments, a character reported by Fauvel (1936a) for *Leiocapitella dollfusi*, has not been confirmed in material previously identified as *Leiocapitella glabra* (Hartman 1947, Ben-Eliahu and Fiege 1995), due to lack of posterior parts. In the present study, in agreement with Bellan (1964a), and Capaccioni-Azzati and El-Haddad (2015), we consider the older name *Leiocapitella dollfusi* as having priority over *Leiocapitella glabra*.

#### Leiocapitellides analis

Hartmann-Schröder, 1960

##### Ecological interactions

###### Native status

Non-native (casual)

##### Notes

Reported from Greece by Akoumianaki (2004) and Neofitou et al. (2014). In the Mediterranean also known from Egypt (Abd-Elnaby 2009). Originally described from the Red Sea.

#### Leiochrus alutaceus

Ehlers, 1908

##### Notes

Questionable status. In the Mediterranean only reported from Greece (Akoumianaki 2004). Commonly distributed in the Pacific. Worldwide, records of this genus are rare and uncertain (e.g. Hilbig and Blake 2006, Probert et al. 2009). However, as species of *Leiochrides* can key out as *Leiochrus* in Fauchald (1977), the presence of the former genus in Greece is possible if the key by Fauchald has been used.

#### Mastobranchus trinchesii

Eisig, 1887

##### Notes

Type locality: Mediterranean (Gulf of Naples).

#### Mediomastus capensis

Day, 1961

##### Ecological interactions

###### Native status

Non-native (casual)

##### Notes

Reported from Greece by Maidanou et al. (2017). Originally from South Africa, in the Mediterranean also known from Italy (Castelli et al. 2008), Cyprus (Çınar 2005), Spain (Capaccioni-Azzati and El-Haddad 2015) and the Adriatic (Mikac 2015). Zenetos et al. (2005) and Zenetos et al. (2010) question the identification of the species in the Mediterranean, but without providing specific information.

#### Mediomastus fragilis

Rasmussen, 1973

#### Neoheteromastus lineus

Hartman, 1960

##### Notes

Questionable status. In the Mediterranean only reported from Greece (Akoumianaki 2004). Commonly distributed in the East Pacific.

#### Neomediomastus glabrus

(Hartman, 1960)

##### Ecological interactions

###### Native status

Non-native (casual)

##### Notes

In the Mediterranean only reported from Greece, by Dando et al. (1995) from shallow hydrothermal vents and by Simboura and Zenetos (2005) from a depth of 1250 m. Commonly distributed in deep sea habitats of the East Pacific, the Chukchi Sea and possibly the Bay of Biscay (Simboura and Zenetos 2005), thus the record by Dando et al. (1995) is considered questionable as it is from a shallow-water habitat.

#### Neopseudocapitella brasiliensis

Rullier & Amoureux, 1979

##### Ecological interactions

###### Native status

Non-native (questionable)

##### Notes

Questionable status. Reported from Greece by NCMR (1992), Simboura (1996) and Katsiaras and Simboura (2015). In the Mediterranean also known from Italy (Gravina and Somaschini 1990), Spain (Capaccioni-Azzati et al. 1992), Cyprus (Çınar 2005), Turkey (Çınar et al. 2014) and the Adriatic (Mikac 2015). Originally described from Brazil. However, Capaccioni-Azzati and El-Haddad (2015) list some noteworthy inconsistencies amongst Mediterranean records and the original description regarding the presence of chaetae on the peristomium and the last setiger with capillaries present. Therefore, it is likely that the Mediterranean records belong to an undescribed species.

#### Notomastus aberans

Day, 1957

##### Ecological interactions

###### Native status

Non-native (established)

##### Notes

Natively distributed in East Africa and the Red Sea, first Mediterranean record from Greece (Harmelin 1968), nowadays distributed throughout the Mediterranean (Capaccioni-Azzati and El-Haddad 2015).

#### Notomastus formianus

Eisig, 1887

##### Notes

Type locality: Mediterranean (Gulf of Naples).

#### Notomastus latericeus

Sars, 1851

##### Notes

Morphologically very similar to *Notomastus profundus* (Eisig, 1887), therefore some authors have suggested a possible synonymy between the two species (Capaccioni-Azzati and El-Haddad 2015; references within).

#### Notomastus lineatus

Claparède, 1869

##### Notes

Reported from Greece by Koukouras (1979) and Papazacharias (1991). Type locality: Mediterranean (Gulf of Naples).

#### Notomastus profundus

(Eisig, 1887)

##### Notes

Type locality: Mediterranean (Gulf of Naples).

#### Peresiella clymenoides

Harmelin, 1968

##### Notes

Originally described from specimens collected in Marseilles and the Greek islands of Crete and Santorini.

#### Pseudocapitella incerta

Fauvel, 1913

##### Notes

Type locality: Mediterranean (French Riviera).

#### Pseudoleiocapitella fauveli

Harmelin, 1964

##### Notes

Type locality: Mediterranean (Gulf of Lion).

### Chaetopteridae Audouin & Milne Edwards, 1833

#### Chaetopterus variopedatus

(Renier, 1804)

##### Notes

Type locality: Mediterranean.

#### Mesochaetopterus sagittarius

(Claparède, 1870)

##### Notes

Type locality: Mediterranean (Gulf of Naples).

#### Phyllochaetopterus gracilis

Grube, 1863

##### Notes

Reported from Greece by Pérès (1959). Type locality: Mediterranean (Adriatic).

#### Phyllochaetopterus socialis

Claparède, 1869

##### Notes

Type locality: Mediterranean (Gulf of Naples).

#### Spiochaetopterus costarum

(Claparède, 1869)

Spiochaetopterus costarum (Claparède, 1869) | *Telepsavus costarum* Claparède 1869

##### Notes

Species complex. *Spiochaetopterus costarum* was long believed to be a cosmopolitan species but in fact the name has been applied to a number of pseudo-cryptic species (e.g. Bhaud 1998, Bhaud et al. 2003). Mediterranean specimens are currently considered to belong to *Spiochaetopterus costarum* sensu stricto, as the type locality of the species is in the Gulf of Naples.

#### Spiochaetopterus solitarius

(Rioja, 1917)

Phyllochaetopterus solitarius Rioja, 1917| *Spiochaetopterus solitarius* (Rioja, 1917)

#### Spiochaetopterus typicus

M. Sars, 1856

##### Notes

Reported from Greece by Makra and Nicolaidou (2000) and Tselepides (1992). In the Mediterranean also reported from France (Guille and Laubier 1966) and the Adriatic (Mikac 2015). Bhaud (1998) restricts the species' distribution to the boreal Atlantic and considers it absent from the temperate waters of the Mediterranean, but the report by Tselepides is from deep waters (500 m and 700 m) off Crete, where environmental conditions could be more favourable for the species. Other boreal species have been shown to occur in deep Mediterranean waters (Fiege et al. 2000a).

### Chrysopetalidae Ehlers, 1864

#### Arichlidon reyssi

(Katzmann, Laubier & Ramos, 1974)

Arichlidon reyssi (Katzmann, Laubier & Ramos, 1974) | *Bhawania reyssi* Katzmann, Laubier & Ramos, 1974

##### Notes

Type locality: Mediterranean (Adriatic).

#### Chrysopetalum debile

(Grube, 1855)

Chrysopetalum debile (Grube, 1855) | *Paleanotus debile* (Grube, 1855)

##### Notes

Type locality: Mediterranean (Villfranche-sur-Mer, France).

#### Hyalopale bispinosa

Perkins, 1985

##### Notes

Recently identified from Greece (Watson and Chatzigeorgiou, submitted) based on two specimens from Crete (for details see Record 1; Record 2).

#### Paleanotus chrysolepis

Schmarda, 1861

### Cirratulidae Carus, 1863

#### Aphelochaeta filiformis

(Keferstein, 1862)

Aphelochaeta filiformis (Keferstein, 1862) | *Cirratulus filiformis* Keferstein, 1862

#### Aphelochaeta marioni

(Saint-Joseph, 1894)

Aphelochaeta marioni (Saint-Joseph, 1894) | *Tharyx marioni* (Saint-Joseph, 1894)

#### Aphelochaeta cf. monilaris

(Hartman, 1960)

##### Notes

In the Mediterranean only reported from Greece (Simboura 1996, as Aphelochaeta cf. monilaris). Commonly distributed along the Pacific coast of North America. The Greek material differs from Hartman's original description mainly in the number of thoracic chaetigers (11 vs. 15 in the species described by Hartman 1960) and in the absence of eyespots (two eyespots in Hartman's material) and may belong to a different taxon (Simboura 1996).

#### Aphelochaeta multibranchis

(Grube, 1863)

Aphelochaeta multibranchis (Grube, 1863) | *Tharyx multibranchis* (Grube, 1863)

##### Notes

Type locality: Mediterranean.

#### Caulleriella alata

(Southern, 1914)

Caulleriella alata (Southern, 1914) | *Heterocirrus alatus* (Southern, 1914)

##### Notes

Mediterranean records could also comprise specimens of the recently described *Caulleriella mediterranea* Lezzi, 2017 which differs from *Caulleriella alata* mainly in having a biannulate instead of triannulate prostomium (Lezzi 2017).

#### Caulleriella bioculata

(Keferstein, 1862)

Caulleriella bioculata (Keferstein, 1862) | *Heterocirrus bioculatus* (Keferstein, 1862)

#### Caulleriella viridis

(Langerhans, 1881)

##### Notes

Reported from Greece by Maidanou et al. (2017). The only other Mediterranean record is an unconfirmed record from Corsica (Ifremer 2016) and neither of the two Mediterranean reports is accompanied by a taxonomic description. Originally described from Madeira, little is known on its distribution range. If its presence is confirmed, the species should probably be considered non-native to the Mediterranean.

#### Chaetozone caputesocis

(Saint-Joseph, 1894)

Caulleriella caputesocis (Saint-Joseph, 1894) | *Chaetozone caputesocis* (Saint-Joseph, 1894)

#### Chaetozone corona

Berkeley & Berkeley, 1941

Chaetozone corona Berkeley & Berkeley, 1941

##### Ecological interactions

###### Native status

Non-native (established)

##### Notes

Reported from Greece by Simboura et al. (2010), found several times afterwards (HCMR, unpublished data). In the Mediterranean also known from Turkey (Çınar et al. 2014) and the Adriatic Sea (Munari et al. 2017). Chambers et al. (2011) doubt the validity of the Mediterranean records and the presence of the species in the Mediterranean (mainly on the basis of its Pacific distribution) but Le Garrec et al. (2016) present evidence of a wide distribution of the species in the Bay of Biscay, indicating that *Chaetozone corona* is an established alien species along the coasts of Europe and was probably introduced via shipping from its native distribution.

#### Chaetozone gibber

Woodham & Chambers, 1994

##### Notes

Reported from Greece by Simboura et al. (2010) and Katsiaras and Simboura (2015). In the Mediterranean also known from Tunisia (Zaâbi et al. 2012), Turkey (Çınar et al. 2014) and the Adriatic (Mikac 2015); otherwise distributed around the British Isles and along the European Atlantic coasts. Chambers et al. (2011) remark that records of *Chaetozone gibber* from the Mediterranean could in fact belong to other species, as the Mediterranean environment differs significantly from conditions in the species' native distribution range.

#### Chaetozone setosa

Malmgren, 1867

##### Notes

Questionable status. Species complex. *Chaetozone setosa* is a complex of pseudo-cryptic species, with at least five species in northern Europe alone (Chambers and Woodham 2003). Several authors (e.g. Simboura 1996, Çınar and Ergen 2007, Mikac 2015) doubt the validity of Mediterranean records, as the species has likely been confused with *Chaetozone gibber* Woodham & Chambers, 1994, *Chaetozone corona* Berkeley & Berkeley, 1941 or *Chaetozone carpenteri* McIntosh, 1911 and probably does not occur in the Mediterranean.

#### Chaetozone zetlandica

McIntosh, 1911

Caulleriella zetlandica (McIntosh, 1911)

##### Notes

Chambers et al. (2011) remark that records of *Chaetozone zetlandica* might be misidentifications, as the Mediterranean environment differs significantly from conditions in the species' native distribution range in the North Atlantic.

#### Cirratulus cirratus

(O. F. Müller, 1776)

##### Notes

Species complex. Often considered cosmopolitan, but ecological, morphological and genetic differences have been found in specimens from different geographical locations (Petersen 1999, Carr et al. 2011, Weidhase et al. 2014).

#### Cirriformia tentaculata

(Montagu, 1808)

Audouinia tentaculata (Montagu, 1808) | *Cirriformia tentaculata* (Montagu, 1808)

#### Dodecaceria capensis

Day, 1961

##### Ecological interactions

###### Native status

Non-native (established)

##### Notes

In the Mediterranean only reported from Greece (e.g. Bogdanos and Satsmadjis 1983, Bogdanos and Satsmadjis 1987, Simboura 1996; full reference list in Suppl. material 2). Known from South Africa. Identification of several Greek specimens confirmed by P. H. Gibson (pers. comm. in Simboura 1996).

#### Dodecaceria concharum

Ørsted, 1843

##### Notes

Questionable status. Castelli et al. (2008) and Mikac (2015) consider the presence of the species in the Mediterranean uncertain as it may have been confused with *Dodecaceria saxicola* (Grube, 1855), *Dodecaceria sextentaculata* (Delle Chiaje, 1822), *Dodecaceria fimbriata* (Verrill, 1880) or *Dodecaceria joubini* Gravier, 1905. Several Greek specimens were examined by P.H. Gibson and differ from the northern European species; instead, different species (e.g. *Dodecaceria joubini*) could be present in Greece (P.H. Gibson, pers. comm. in Simboura 1996). The identification of *Dodecaceria* species depends on reproductive characteristics (Gibson 1978) but it is unknown which form was described by Ørsted. Thus, identity of *Dodecaceria concharum* is confused and Ørsted's original species is probably not valid due to an insufficient original description and loss of type material (Gibson 2015). Neotypes were designated by Gibson and Heppell (1995), but it is unclear whether these correspond to the original species of Ørsted.

#### Dodecaceria saxicola

(Grube, 1855)

##### Notes

Reported from Greece by Chatzigeorgiou et al. (2016). Type locality: Mediterranean (Villefranche-sur-Mer, France). As the species had long been treated as a synonym of *Dodecaceria concharum* Ørsted, 1843 it may be underreported.

#### Kirkegaardia dorsobranchialis

(Kirkegaard, 1959)

Monticellina dorsobranchialis (Kirkegaard, 1959) | *Tharyx dorsobranchialis* (Kirkegaard, 1959)

##### Notes

A species reported from worldwide locations, partly caused by inaccurate descriptions in the past; therefore Blake (2016) considers the presence of *Kirkegaardia dorsobranchialis* in the Mediterranean uncertain and possibly restricted to West and South Africa. Many Greek records of *Kirkegaardia dorsobranchialis* belong in fact to *Kirkegaardia heterochaeta* (Laubier, 1961), as Blake (1991) had synonymised *Monticellina heterochaeta* with *Monticellina dorsobranchialis*. The distinction between the two species is difficult. *Kirkegaardia heterochaeta* has a longer peristomium than *Kirkegaardia dorsobranchialis* and posesses a mid-dorsal ridge within the dorsal channel which is absent in *Kirkegaardia dorsobranchialis*. The most reliable factor for their separation is the methyl green staining pattern (Blake 2016).

#### Kirkegaardia heterochaeta

(Laubier, 1961)

Monticellina heterochaeta Laubier, 1961| *Tharyx heterochaeta* (Laubier, 1961)

##### Notes

Possibly underreported, as the species was considered a synonym of *Kirkegaardia dorsobranchialis* (Kirkegaard, 1959) by Blake (1991) and reported under the latter name until Blake (1996) revoked the synonymy. Type locality: Mediterranean (France).

#### Kirkegaardia tesselata

(Hartman, 1960)

Monticellina tesselata (Hartman, 1960)

##### Notes

Questionable status. Reported from Greece by Koulouri et al. (2015). In the Mediterranean also reported from Spain (Desbruyères et al. 1973) and Turkey (Çınar et al. 2014). Çınar (2005) reports morphological differences in chaetal features between specimens from Cyprus and the original description of *Kirkegaardia tesselata*, concluding that the material might belong to an undescribed species. Blake (2016), having examined a specimen of *Kirkegaardia* from France, refers this specimen and the literature references of *Monticellina tesselata* by Çınar et al. (2014) and Çınar (2005) to an undescribed *Kirkegaardia* species (*Kirkegaardia* sp. A). He restricts the distribution of *Kirkegaardia tesselata* to California. Greek specimens possibly do not belong to *Kirkegaardia tesselata* but the material would require re-examination to confirm its identity.

#### Protocirrineris chrysoderma

(Claparède, 1868)

Cirratulus chrysoderma Claparède, 1868

##### Notes

Questionable status; species complex. Reported from Greece by NCMR (1989). Reported from worldwide locations but the species constitutes a complex of pseudo-cryptic species (Petersen 1991). Originally described from the Gulf of Naples and the only known species of the genus in the Mediterranean until the recent description of *Protocirroneris purgamentorum* Lezzi, Çınar & Giangrande, 2016. While it is possible that the species occurs in Greece, it has so far only been reported in grey literature and has not been found in almost three decades, therefore its presence in Greece is here considered questionable.

#### Tharyx killariensis

(Southern, 1914)

Caulleriella killariensis (Southern, 1914)| *Heterocirrus killariensis* (Southern, 1914) | *Tharyx killariensis* (Southern, 1914)

#### Timarete filigera

(Delle Chiaje, 1828)

Cirriformia filigera (Delle Chiaje, 1828) | *Timarete filigera* (Delle Chiaje, 1828)

##### Notes

Species complex. *Timarete filigera* has been recorded from worldwide locations, but many of these records probably belong to different species. Specimens from West Atlantic localities identified as *Timarete filigera* were re-examined morphologically and genetically by Magalhães et al. (2014) and shown to belong to undescribed species or to species previously treated as synonyms of *Timarete filigera*. Mediterranean specimens are currently considered to belong to *Timarete filigera* sensu stricto, as the type locality of the species is in the Gulf of Naples.

#### Timarete punctata

(Grube, 1859)

##### Ecological interactions

###### Native status

Non-native (casual)

##### Notes

Species complex. *Timarete punctata*, originally described from the Caribbean, constitutes a complex of cryptic species. Molecular analyses of specimens morphologically identified as *Timarete punctata* by Seixas et al. (2017) revealed that samples from the West Atlantic and the Pacific comprise two cryptic species, one of them widely distributed and exhibiting a low genetic diversity, the other restricted to a single location. The authors hypothesise that the low genetic diversity of the widely distributed cryptic species could be attributed to a recent human-mediated introduction at these sites. In the Mediterranean reported from Greece (Maidanou et al. 2017) and Turkey (Çınar et al. 2014).

### Cossuridae Day, 1963

#### Cossura coasta

Kitamori, 1960

##### Ecological interactions

###### Native status

Non-native (questionable)

##### Notes

Questionable status. Arvanitidis (1994) questions the presence of the species in the area, as the description of the specimen found by Bogdanos and Fredj (1983) shows differences both to the species described by Kitamori (1960) and to *Cossura soyeri* Laubier, 1964. Without re-examination of the Mediterranean specimens referred to *Cossura coasta*, the status of the species in the area remains doubtful.

#### Cossura soyeri

Laubier, 1964

##### Notes

Type locality: Mediterranean (Banyuls-sur-Mer, France)

### Ctenodrilidae Kennel, 1882

#### Ctenodrilus serratus

(Schmidt, 1857)

##### Notes

Reported from Greece by Makra and Nicolaidou (2000), also found by Papageorgiou et al. (2006) (unpublished dataset). Type locality: Mediterranean.

### Dorvilleidae Chamberlin, 1919

#### Dorvillea rubrovittata

(Grube, 1855)

Dorvillea rubrovittata (Grube, 1855) | *Schistomeringos rubrovittata* (Grube, 1855) | *Staurocephalus rubrovittatus* Grube, 1855

##### Notes

Type locality: Mediterranean (Adriatic). Considered cosmopolitan. However, Jumars (1974) notes differences between populations and suggests the possibility of more than one species existing under the name of *Dorvillea rubrovittata*.

#### Dorvillea similis

(Crossland, 1924)

##### Ecological interactions

###### Native status

Non-native (casual)

##### Notes

Reported from Greece by Corsini-Foka et al. (2015). In the Mediterranean also known from Turkey (Çınar 2009), natively distributed in the Indo-Pacific Ocean and Red Sea.

#### Ophryotrocha labronica

La Greca & Bacci, 1961

##### Notes

Species complex. Reported from Greece by Åkesson (1984). Neotype from the Gulf of Naples. At least 15 sibling species have been reported under the *Ophryotrocha labronica* group worldwide (Åkesson and Paxton 2005), four of which have been recently described from areas including the Mediterranean by Paxton and Åkesson (2010): *Ophryotrocha japonica, Ophryotrocha macrovifera, Ophryotrocha robusta* and *Ophryotrocha rubra*.

#### Ophryotrocha puerilis

Claparède & Mecznikow, 1869

Ophryotrocha puerilis Claparède & Mecznikow, 1869 | *Ophryotrocha puerilis puerilis* Claparède & Mecznikow, 1869

##### Notes

Species complex. Neotype from Genoa, Italy. Simboura and Nicolaidou (2001) report *Ophryotrocha puerilis* from Greece at species level, as it is reported on other parts of the Mediterranean as well (Castelli et al. 2008, Çınar et al. 2014, Núñez et al. 2013, Mikac 2015). However, Taboada et al. (2017) provide molecular evidence of at least two cryptic species under the name *Ophryotrocha puerilis* in the Mediterranean Sea.

#### Parougia caeca

(Webster & Benedict, 1884)

Schistomeringos caeca (Webster & Benedict, 1884)

##### Notes

Reported from Greece by Eleftheriou et al. (1990) and Dando et al. (1995). In the Mediterranean also known from Italy (Gambi et al. 1998) and Turkey (Çınar et al. 2014); otherwise distributed in the North Atlantic and the North-East Pacific.

#### Pettiboneia urciensis

Campoy & San Martín, 1980

##### Notes

Type locality: Mediterranean (Águilas, Spain).

#### Protodorvillea atlantica

(McIntosh, 1885)

Protodorvillea atlantica (McIntosh, 1885) | *Schistomeringos atlanticus* (McIntosh, 1885)

##### Notes

Questionable status. Reported from Greece by Vamvakopoulou (1991). In the Mediterranean also known from Tunisia (Zaâbi et al. 2009) and the Adriatic (Mikac 2015). Originally described from deep waters off the Azores (Atlantic); as the Greek record is from a lagoonal habitat, it must be considered questionable.

#### Protodorvillea biarticulata

Day, 1963

##### Ecological interactions

###### Native status

Non-native (established)

##### Notes

In the Mediterranean reported from Greece (Bogdanos and Satsmadjis 1983, Bogdanos and Diapoulis 1984, Simboura et al. 1995b) and Egypt (Abd-Elnaby and Gab-Alla 2007), otherwise distributed in the Indo-Pacific, South Africa and Brazil.

#### Protodorvillea kefersteini

(McIntosh, 1869)

Protodorvillea kefersteini (McIntosh, 1869) | *Schistomeringos kefersteini* (McIntosh, 1869) | *Staurocephalus kefersteini* McIntosh, 1896

#### Schistomeringos neglecta

(Fauvel, 1923)

Dorvillea neglecta (Fauvel, 1932) | *Schistomeringos neglecta* (Fauvel, 1923)

#### Schistomeringos rudolphi

(Delle Chiaje, 1828)

Dorvillea rudolphi (Delle Chiaje, 1828) | *Schistomeringos rudolphi* (Delle Chiaje, 1828) | *Staurocephalus rudolphi* (Delle Chiaje, 1828)

##### Notes

Type locality: Mediterranean (Gulf of Naples).

### Eulepethidae Chamberlin, 1919

#### Grubeulepis augeneri

Pettibone, 1969

##### Notes

One specimen from Greece in the collections of the Senckenberg Museum (SMF 15256, 35°52'58.2"N, 27°42'49.2"E, 115 m depth, coll. date 2005-09-05, det. D. Fiege). Type locality: Mediterranean (Adriatic).

#### Grubeulepis katzmanni

Pettibone, 1986

##### Notes

Questionable status. Reported from Greece by Simboura (1987) and Zenetos et al. (1997). Type locality: Mediterranean (Adriatic). The original description by Pettibone (1986) is based on juveniles and needs to be completed with adult characters; *Grubeulepis katzmanni* may turn out to be a juvenile of *Grubeulepis augeneri* Pettibone, 1969 (Pettibone 1986).

### Eunicidae Berthold, 1827

#### Eunice aphroditois

(Pallas, 1788)

##### Notes

Questionable status. Fauvel (1923) recorded *Eunice roussaei* Quatrefages, 1866 in the Adriatic Sea, but the species was later synonymised with *Eunice aphroditoi*s by Hartman (1944). Subsequently, several records of large-sized eunicids in the Mediterranean were assigned to the latter. After the resurrection of *Eunice roussaei* by Fauchald (1992), many authors suggested that literature records of *Eunice aphroditois* in the Mediterranean actually belong to *Eunice roussaei* (Parapar and Harto 2001, Zanol and Bettoso 2006, Çınar et al. 2014, Mikac 2015). Two specimens of *Eunice aphroditois* from the collections of the Aristotle University of Thessaloniki were re-examined by the first author and identified as *Eunice roussaei* (Faulwetter et al. 2013); it is likely that all Greek records belong to *Eunice roussaei*.

#### Eunice floridana

(Pourtalès, 1867)

##### Ecological interactions

###### Native status

Non-native (questionable)

##### Notes

Questionable status. Reported from Greece by Kisseleva (1983). In the Mediterranean also known from the western basin (e.g. Bellan 1959, Fredj 1974), Cyprus (Argyrou et al. 1999) and the Adriatic (Casellato and Stefanon 2008). Otherwise distributed in the Greater Caribbean (Fauchald et al. 2009). Commonly occurs in coralligenous habitats (Casellato and Stefanon 2008), which are little studied in Greece. However, no clear description based on Mediterranean material exists. Fauvel (1923), considers *Leodice gunneri* (Storm, 1880) a synonym of *Eunice floridana*. Day (1967) lists *Eunice floridana* sensu Fauvel (1923) as a synonym of *Eunice norvegica* (Linnaeus, 1767) and Fauchald (1992) considers *Leodice gunneri* a synonym of the latter. Clear differentiating characters between *Eunice floridana* and *Eunice norvegica* can be found in Fauchald (1992) and Carrera-Parra and Salazar-Vallejo (1998) (which were, however, not published at the time the species was recorded from Greece).

#### Eunice norvegica

(Linnaeus, 1767)

##### Notes

Questionable status. Reported from Greece by NCMR (1989). In the Mediterranean also known from the western basin (e.g. Danovaro et al. 2010, Taviani et al. 2015), Tunisia (Ayari et al. 2009), the Italian coasts of the Ionian Sea (e.g. Tursi et al. 2004, Vertino et al. 2010) and the Adriatic (D'Onghia et al. 2015), otherwise distributed in the North Atlantic. Often associated with deep-water corals (Danovaro et al. 2010), which are little studied in Greece. The species has so far only been reported in grey literature and has not been found in almost three decades, therefore its presence in Greece is pending confirmation. See also notes under *Eunice floridana* on the confusion regarding the species concept in early works and keys.

#### Eunice pennata

(Müller, 1776)

#### Eunice purpurea

Grube, 1866

##### Notes

Reported from Greece by Arvanitidis (1994) and Kitsos (2003). Type locality: Mediterranean (Adriatic). Arvanitidis (1994) notes some differences in the individuals he examined compared to the description provided by Fauchald (1992). Fauvel (1917) regards *Eunice purpurea* as junior synonym of *Eunice roussaei* Quatrefages, 1866 and the species has since then been part of the confusion concerning large eunicids in the Mediterranean (see remarks under *Eunice aphroditois* Pallas, 1788). Fauchald (1992) considers it a valid species by investigating material from the Adriatic and Zanol and Bettoso (2006) highlight specific differences that do not occur in *Eunice roussaei*.

#### Eunice roussaei

Quatrefages, 1866

##### Notes

Reported from Greece by Kisseleva (1983). Probably underreported, as records of *Eunice aphroditois* (Pallas, 1788) from Greece likely belong to *Eunice roussaei* (see notes under *Eunice aphroditois*).

#### Eunice schizobranchia

Claparède, 1870

##### Notes

Questionable status. Reported from Greece by NCMR (1992). Type locality: Mediterranean (Gulf of Naples). Commonly occurs in coralligenous habitats (Casellato and Stefanon 2008), which are little studied in Greece. While it is possible that the species occurs in Greece, it has so far only been reported in grey literature and has not been found in over two decades, therefore its presence in Greece is here considered questionable.

#### Eunice vittata

(Delle Chiaje, 1828)

##### Notes

Type locality: Mediterranean (Gulf of Naples).

#### Leodice harassii

(Audouin & Milne Edwards, 1833)

Eunice harassii Audouin & Milne Edwards, 1833

##### Notes

Records of *Leodice harassii* could include specimens of *Eunice rubrocincta* Ehlers, 1868, a Mediterranean species which Fauvel (1923) considered synonymous with *Leodice harassii*. *Leodice rubrocincta* was resurrected by Fauchald (1992) and is included as a valid species in the key by Carrera-Parra and Salazar-Vallejo (1998).

#### Leodice torquata

(Quatrefages, 1866)

Eunice torquata Quatrefages, 1866 | *Leodice torquata* (Quatrefages, 1866)

##### Notes

The species resembles *Leodice laurillardi* (Quatrefages, 1866) in a number of diagnostic characters. The latter was synonymised with *Leodice torquat*a by Pruvot and Racovitza (1895); this was accepted by Fauvel (1923) and later authors. However, Fauchald (1992) resurrected *Leodice laurillardi* as a valid species and Arias et al. (2015) re-described the species based on type material and new specimens from the Mediterranean. They suggest that *Leodice laurillardi* could be much more widespread than previously thought but was possibly being overlooked in the Mediterranean due to the confusion with *Leodice torquata*.

#### Lysidice collaris

Grube, 1870

##### Ecological interactions

###### Native status

Non-native (questionable)

##### Notes

Questionable status. Frequently reported from Greece and other parts of the Mediterranean (Martín 1987, Sardá 1991, Giangrande et al. 2003, Mikac 2015). However, Çınar (2005) and Kurt Şahin and Çınar (2009) suggest that the species closely resembles the native species *Lysidice margaritacea* Claparède, 1868 and question the presence of *Lysidice collaris* in the Eastern Mediterranean. Type locality in the Red Sea, but reported from circumtropical locations.

#### Lysidice ninetta

Audouin & Milne Edwards, 1833

##### Notes

Species complex. Frequently reported from Greece. However, Iannotta et al. (2006) and Iannotta et al. (2009) investigated molecular markers and provided evidence of sibling species within the *Lysidice ninetta* complex. Two morphotypes (dark and light), differentiated by colour patterns in anterior body and acicular chaetae, were identified. Although the dark type matches well the original description, it is still unresolved whether one or both morphotypes are new species or belong to previously described species.

#### Lysidice unicornis

(Grube, 1840)

Lysidice unicornis (Grube, 1840) | *Nematonereis unicornis* (Grube, 1840)

##### Notes

The genus *Nematonereis* Schmarda, 1860 was synonymised with *Lysidice* Lamarck, 1818 by Zanol et al. (2013). Type locality: Mediterranean (Adriatic).

#### Marphysa adenensis

Gravier, 1900

##### Ecological interactions

###### Native status

Cryptogenic

##### Notes

In the Mediterranean only reported from Greece (Katsiaras et al. 2014) from *Posidonia oceanica* meadows, a little studied habitat in Greece. Distributed in the Indo-Pacific. The authors note that data are currently insufficient to draw conclusions on the species' native or alien distribution.

#### Marphysa bellii

(Audouin & Milne Edwards, 1833)

#### Marphysa cinari

Kurt-Şahin, 2014

##### Notes

Several Greek specimens reported by Simboura et al. (2010) as *Marphysa disjuncta* Hartman, 1961 were re-examined by Kurt Şahin (2014) and assigned to *Marphysa cinari*. Type locality: Sea of Marmara.

#### Marphysa fallax

Marion & Bobretzky, 1875

##### Notes

Type locality: Mediterranean (Marseille).

#### Marphysa kinbergi

McIntosh, 1910

#### Marphysa sanguinea

(Montagu, 1813)

##### Notes

Species complex. Considered cosmopolitan in the past, but *Marphysa sanguinea* actually comprises a complex of numerous pseudo-cryptic species (Zanol et al. 2016). Hutchings and Karageorgopoulos (2003) designated a neotype based on pectinate chaetae distribution instead of branchiae and Hutchings et al. (2012) encourage re-examination of all material outside the English Channel and North Sea. Recent studies have described several new species similar to *Marphysa sanguinea* or resurrected species from synonymy with the latter (Lewis and Karageorgopoulos 2008, Molina-Acevedo and Carrera-Parra 2015, Zanol et al. 2016, Liu et al. 2017). Wijnhoven and Dekker (2010) question the presence of *Marphysa sanguinea* in the Mediterranean Sea and encourage comparisons of specimens with the neotype.

#### Palola siciliensis

(Grube, 1840)

Eunice siciliensis Grube, 1840 | *Palola siciliensis* (Grube, 1840)

##### Notes

Type locality: Mediterranean (Palermo, Sicily, Italy).

### Euphrosinidae Williams, 1852

#### Euphrosine foliosa

Audouin & Milne Edwards, 1833

#### Euphrosine myrtosa

Savigny in Lamarck, 1818

##### Notes

Questionable status. Reported from Greece by Bogdanos and Satsmadjis (1983). In the Mediterranean also reported from the Levantine Basin (Ben-Eliahu 1995) and questionably from the Adriatic (Mikac 2015), otherwise distributed in the Indo-Pacific. It might either be an overlooked alien species in the area or a misidentification.

### Fabriciidae Rioja, 1923

#### Fabricia stellaris stellaris

(Müller, 1774)

Fabricia sabella (Ehrenberg, 1836) | *Fabricia stellaris stellaris* (O. F. Müller, 1774) |

#### Manayunkia aestuarina

(Bourne, 1883)

##### Notes

Questionable status. In the Mediterranean only reported from Greece (Kisseleva 1961, Diapoulis and Bogdanos 1983). Both Greek records are from a fully marine environment and therefore considered questionable by Arvanitidis (1994), as the species usually occurs in brackish waters. However, Harris (1970) reports the species from non-brackish environments in the British Isles and assumes that it can tolerate full salinity at least during certain stages of its life cycle. Based on the geographic range of *Manayunkia aestuarina*, the Greek records must nevertheless be considered uncertain. Undescribed species of *Manayunkia* exist in Greece (Chintiroglou et al. 2004) and the genus needs further study in the region.

#### Novafabricia posidoniae

Licciano & Giangrande, 2006

##### Notes

New record for Greece. Eleven specimens, Lesvos Island, 38°59'54''N, 26°32'26''E, 6 m depth, *Posidonia oceanica* meadow, collected in the framework of the PhD of N. Katsiaras. Literature used for identification: Licciano and Giangrande (2006). Type locality: Mediterranean (Italy).

#### Pseudofabricia aberrans

Cantone, 1972

##### Notes

Reported from Greece by Simboura and Zenetos (2005). Type locality: Mediterranean (Sicily).

#### Pseudofabriciola analis

Fitzhugh, Giangrande & Simboura, 1994

##### Notes

Specimens of *Fabricia filamentosa* Day 1963 from Greece (Simboura 1990) and the Adriatic (Giangrande and Castelli 1986) were re-examined by Fitzhugh et al. (1994) who described two new species based on this material: *Pseudofabriciola analis* from the Adriatic and *Pseudofabriciola longipyga* Fitzhugh, Giangrande & Simboura, 1994 from Greece. *Pseudofabriciola analis* was subsequently reported from Greece by Simboura (1996).

#### Pseudofabriciola longipyga

Fitzhugh, Giangrande & Simboura, 1994

##### Notes

Originally described from Greece by Fitzhugh et al. (1994). See remarks under *Pseudofabriciola analis* Fitzhugh, Giangrande & Simboura, 1994.

### Flabelligeridae de Saint-Joseph, 1894

#### Bradabyssa villosa

(Rathke, 1843)

Brada villosa (Rathke, 1843)

##### Notes

Questionable status. Species complex. Salazar-Vallejo (2017) revised the genus *Brada* and transferred a large number of species (including *Brada villosa*) to *Bradabyssa* Hartman, 1967. Previous descriptions of *Bradabyssa villosa* by e.g. Fauvel (1927), comprise a wide variety of characters and probably comprise more than one species under this name (Salazar-Vallejo 2017). Likewise, molecular analyses by Carr et al. (2011) revealed at least two putative cryptic species in Canadian waters (one in the Arctic, another in the Pacific Ocean) which were initially identified as *Bradabyssa villosa*. Salazar-Vallejo (2017) restricts *Bradabyssa villosa* sensu stricto to the North Atlantic Ocean and European and Russian Arctic and re-instates *Bradabyssa parthenopeia* (Lo Bianco, 1893) from the Mediterranean (Gulf of Naples) – a species previously considered a synonym of *Bradabyssa villosa*. It is highly likely that *Bradabyssa villosa i*s absent from the Mediterranean and specimens from this region belong to other species such as *Bradabyssa parthenopeia*.

#### Diplocirrus glaucus

(Malmgren, 1867)

#### Diplocirrus hirsutus

(Hansen, 1878)

##### Notes

Questionable status. One specimen from Greece in the collections of the Senckenberg Museum (SMF 11285, 39°09'46.8"N, 19°26'21.6"E, 1008 m depth, coll. date 1993-05-19, det. D. Fiege). In the Mediterranean also reported from the Adriatic, but records are considered doubtful by Mikac (2015) based on the Arctic and Subarctic distribution of the species.

#### Flabelligera affinis

M. Sars, 1829

##### Notes

Species complex. Consists of at least two different putative cryptic species in the Arctic Ocean (Carr et al. 2011), specimens from other locations could belong to either of these or to a different cryptic species. Salazar-Vallejo (2012) considers the distribution of *Flabelligera affinis* to be restricted to “Arctic to cold and temperate, boreal localities” and questions the identity of specimens reported under this name from warm water localities such as Africa or Panama. Whether the Mediterranean is considered here temperate or warm water and whether the species occurs in the Mediterranean is unknown; it is considered here to be present in the Greek waters pending further information.

#### Flabelligera diplochaitus

(Otto, 1820)

##### Notes

Type locality: Mediterranean.

#### Pherusa plumosa

(Müller, 1776)

Pherusa plumosa (Müller, 1776) | *Stylarioides plumosa* (O.F. Müller, 1776)

##### Notes

Questionable status. Salazar-Vallejo (2014) revised the genus *Pherusa* and re-instated a number of species, many of which were previously considered synonyms of *Pherusa plumosa*. He restricts *Pherusa plumosa* in its diagnosis and distribution; currently it is believed to occur in the Arctic and temperate Atlantic Ocean and to be absent from the Mediterranean. The three currently known species of *Pherusa* occurring in the Mediterranean Sea – previously all grouped under the name *Pherusa plumosa* – are *Pherusa incrustans* Quatrefages, 1866, *Pherusa mikacae* Salazar-Vallejo, 2014 and *Pherusa obscura* Quatrefages, 1849. As the description by Fauvel (1927) is very general and includes a large range of characters which correspond to *Pherusa plumosa* sensu stricto, *Pherusa obscura* and *Pherusa incrustans* (Salazar-Vallejo 2014), all specimens identified using Fauvel’s key are questionable.

#### Piromis eruca

(Claparède, 1869)

Pherusa eruca (Claparède, 1869) | *Piromis eruca* (Claparède, 1869) | *Stylarioides eruca* (Claparède, 1869)

##### Notes

Type locality: Mediterranean (Gulf of Naples)

#### Stylarioides monilifer

Delle Chiaje, 1841

Pherusa monilifera (Delle Chiaje, 1841)

##### Notes

Salazar-Vallejo (2011) revised the genus *Stylarioides* and reinstated *Stylarioides hirsutus* Lo Bianco, 1893, which Fauvel (1927) had synonymised with *Stylarioides monilifer*. Thus, specimens identified using the publication by Fauvel (1927) or works based on it (e.g. Castelli 1990) could belong to either of the two species. Type locality: Mediterranean (Gulf of Naples).

#### Therochaeta flabellata

(Sars in Sars, 1872)

Pherusa flabellata (M. Sars in G.O. Sars, 1872) | *Therochaeta flabellata* (M. Sars in G.O. Sars, 1872)

##### Notes

Questionable status. Salazar-Vallejo (2013) restricts *Therochaeta flabellata* to the North-East Atlantic Ocean and Arctic waters and assigns Mediterranean material, including the description by Fauvel (1927) to an undescribed species (informally named Therochaeta cf. flabellata “Mediterranean”, formal description pending). The Mediterranean form differs from the original description mainly in the size of the much larger sediment particles and in the lack of fusion of the first two chaetigers.

### Glyceridae Grube, 1850

#### Glycera alba

(O.F. Müller, 1776)

Glycera alba (O.F. Müller, 1776) | *Glycera minuta* (Bobretzky, 1870)

##### Notes

Böggemann (2002) distinguishes the species from *Glycera tridactyla* Schmarda, 1861, mainly on the basis of the shape of the proboscidial papillae and ailerons; although the separation was judged problematic by Worsfold (2007). Parapar and Moreira (2015) agree in separating the two species on the basis of the above two characters.

#### Glycera capitata

Ørsted, 1843

##### Notes

Frequently reported from the Mediterranean (e.g. Castelli et al. 2008, Ayari et al. 2009, Çınar et al. 2014, Mikac 2015), but Böggemann (2002) and Böggemann (2015) restricts its distribution to Arctic, Antarctic, cold temperate areas and deep sea down to 5655m (Böggemann 2015). The Mediterranean material could possibly be *Glycera lapidum* Quatrefages, 1866 or *Glycera noelae* Böggemann, Bienhold & Gaudron, 2012 (Böggemann, pers. comm.)

#### Glycera celtica

O'Connor, 1987

##### Notes

Böggemann (2002) synonymised *Glycera dayi* O' Connor, 1987 with *Glycera celtica* but this synonymy has not been accepted unanimously (Worsfold 2007, Parapar and Moreira 2015).

#### Glycera fallax

Quatrefages, 1850

Glycera gigantea Quatrefages, 1866

#### Glycera lapidum

Quatrefages, 1866

##### Notes

O'Connor (1987) reports at least four varieties amongst the examined material from the North-East Atlantic, although these correspond in fact to different species (Böggemann, pers. comm.).

#### Glycera rouxii

Audouin & Milne Edwards, 1833

##### Notes

Böggemann (2002) considers *Glycera rouxii* a synonym of *Glycera unicornis* Lamarck, 1818 (based on comparisons with original description as the holotype is lost). He considers the shape of the retractable branchiae (reported as bifid in *Glycera unicornis*) not to be a diagnostic character, since variations were found in the same populations (Böggeman, pers. comm.). However, Worsfold (2007) and Parapar and Moreira (2015) debate this synonymy and highlight differences in parapodial lobes besides those of the branchiae, although parapodial lobes were found to change between juvenile and adult specimens by Böggemann (2002). They propose that the branchial shape remains a diagnostic character, which should however be used with caution due to retractability. Despite the available evidence for the synonymy, two recent works consider both species as valid. Therefore, the past records of *Glycera rouxii* in Greece (see Supplementary material) are currently retained as separate, until a response to the arguments of Parapar and Moreira (2015) is available. Type locality: Mediterranean (Marseille).

#### Glycera tesselata

Grube, 1863

##### Notes

Type locality: Mediterranean (Croatia).

#### Glycera tridactyla

Schmarda, 1861

Glycera convoluta Keferstein, 1862 | *Glycera tridactyla* Schmarda, 1861

##### Notes

Böggemann (2002) considers *Glycera convoluta* a synonym of *Glycera tridactyla* based on comparisons with the original description and illustrations. A number of authors support this synonymy (e.g. Worsfold 2007, Parapar and Moreira 2015).

#### Glycera unicornis

Lamarck, 1818

##### Notes

See notes under *Glycera rouxii* Audouin & Milne Edwards, 1833.

### Goniadidae Kinberg, 1865

#### Glycinde bonhourei

Gravier, 1904

##### Ecological interactions

###### Native status

Non-native (casual)

##### Notes

Reported from Greece by Simboura (2008). In the Mediterranean also known from Israel (Böggemann 2005), Egypt (Ben-Eliahu 1972a) and Turkey (Çınar et al. 2014). Originally described from Bonhoure (Djibuti).

#### Glycinde nordmanni

(Malmgren, 1866)

Eone nordmanni Malmgren, 1866 | *Glycinde nordmanni* (Malmgren, 1866)

#### Goniada emerita

Audouin & Milne Edwards, 1833

##### Notes

Type locality: Mediterranean (Nice, France).

#### Goniada gigantea

(Verrill, 1885)

##### Notes

Reported from Greece by Böggemann (2005) from material collected by E. Marenzeller in 1894 on the Cyclades plateau (as *Goniada emerita* Audouin & Milne Edwards, 1833). In the Mediterranean also known from Spain and France (Böggemann 2005). Otherwise reported from the West and East Atlantic and North-East Pacific (Böggemann 2005).

#### Goniada hexadentes

Böggemann & Eibye-Jacobsen, 2002

##### Notes

Reported from Greece by Böggemann (2005) from the abyssal zone (3848 m) off the Peloponnese. In the Mediterranean also known from Spain, Italy and Israel (Böggemann 2005), otherwise reported from the Eastern Atlantic and the Indian Ocean (Böggemann 2005).

#### Goniada maculata

Ørsted, 1843

#### Goniada norvegica

Ørsted, 1845

#### Goniadella bobrezkii

(Annenkova, 1929)

##### Notes

In the Mediterranean only reported from Greece (Arvanitidis 2000a). Commonly distributed in the Black Sea (Annenkova 1929) and around the North-East Atlantic (Wolff and Stegenga 1975, Kirkegaard 2001).

#### Goniadella gracilis

(Verrill, 1873)

##### Notes

Reported from Greece by Bogdanos and Satsmadjis (1983) and Bogdanos and Satsmadjis (1987). In the Mediterranean also known from Israel (Böggemann 2005), otherwise reported from the North-West Atlantic, East Atlantic and South-West Indian Ocean (Böggemann 2005).

### Hesionidae Grube, 1850

#### Gyptis propinqua

Marion & Bobretzky, 1875

Gyptis propinqua Marion & Bobretzky, 1875 | *Oxydromus propinquus* (Marion & Bobretzky, 1875)

##### Notes

Type locality: Mediterranean (France).

#### Hesione splendida

Savigny in Lamarck, 1818

Hesione pantherina Risso, 1826 | *Hesione splendida* Savigny in Lamarck, 1818

##### Notes

*Hesione* species display a high colour variability and it is currently unknown whether these are colour morphs of the same species or belong to different species (Mikac 2015, Jimi et al. 2017). For this reason, the status of *Hesione pantherina, Hesione splendida* and similar species is still unclear; here we follow the opinion of F. Pleijel (pers. comm. to Mikac 2015) and list occurrences of *Hesione pantherina* under *Hesione splendida*.

#### Hesionides arenaria

Friedrich, 1937

##### Notes

Schmidt and Westheide (2000) assess the genetic differentiation of worldwide *Hesionides arenaria* populations. Specimens from Crete group with those from near the type locality in the North Sea, indicating the presence of *Hesionides arenaria* sensu stricto in Greece.

#### Hesionides gohari

Hartmann-Schröder, 1960

##### Notes

Species complex. Schmidt and Westheide (1999) recover three genetic lineages from worldwide localities which could correspond to cryptic species. Specimens from Crete group with those from near the type locality in the Red Sea, indicating the presence of *Hesionides gohari* sensu stricto in Greece.

#### Hesiospina aurantiaca

(M. Sars, 1862)

Hesiospina aurantiaca (M. Sars, 1862) | *Hesiospina similis* (Hessle, 1925)

##### Notes

Frequently reported from Greece, considered cosmopolitan (Pleijel 2004). Simboura (1996) notes morphological differences between specimens of *Hesiospina similis* from the Mediterranean and from the native range (Japan). Specifically, Mediterranean material exhibits a stronger serration of the blades of the compound chaetae and neuropodial aciculae with rounded instead of tapering tips.

#### Leocrates atlanticus

(McIntosh, 1885)

##### Notes

Reported by von Marenzeller (1902) from deep waters (808 m) off Milos. Marenzeller's two specimens (Natural History Museum Vienna, Inv. No. 599, Acq. No. 15503) were examined by S. Faulwetter. One specimen was confirmed as *Leocrates atlanticus*; in the second specimen the jaws could not be observed without dissection and its identity is not confirmed.

#### Leocrates claparedii

(Costa in Claparède, 1868)

##### Notes

Synonymised by Hartman (1965a) with *Leocrates chinensis* Kinberg, 1866, *Leocrates claparedii* was reported under the former name for several years (see also remarks in Table 1), therefore listed in inventories of non-native species in the Mediterranean (e.g. Zenetos et al. 2010). Pleijel (1998), in his revision of the Hesionidae, considers the two species distinct. Type locality: Mediterranean (Gulf of Naples).

#### Microphthalmus aberrans

(Webster & Benedict, 1887)

##### Notes

Questionable status. Reported from Greece by Akoumianaki and Hughes (1997), also found by Papageorgiou et al. (2006) (unpublished data), but *Microphthalmus aberrans* is a taxonomically confused species. Riser (2000) revised the species and found the slides of the type material to be comprised of two different species. He assigned specimens previously assigned to *Microphthalmus aberrans* to three species: *Microphthalmus aberrans, Microphthalmus pettiboneae* Riser, 2000 and *Microphthalmus aggregatus* Riser, 2000. However, the species re-described as *Microphthalmus aberrans* by Riser does not resemble previously available descriptions of the species, as those were mostly copied from a re-description by Southern (1914), which was based on the wrong microscope slide. The specimen on this slide was re-described as *Microphthalmus pettiboneae* by Riser, causing confusion in the application of the name *Microphthalmus aberrans*. All specimens reported under this name would need to be re-investigated to clarify their identity.

#### Microphthalmus fragilis

Bobretzky, 1870

##### Notes

Reported from Greece by Koukouras (1979), also found by Papageorgiou et al. (2006) (unpublished data). In the Mediterranean also known from Italy (Castelli et al. 2008), otherwise distributed in the Black and North Seas.

#### Microphthalmus pseudoaberrans

Campoy & Vieitez, 1982

##### Notes

Reported from Greece by NCMR (1995) and NCMR (2000a). In the Mediterranean also known from Spain (Capaccioni-Azzati and Torres-Gavila 1989), otherwise distributed along the Atlantic coast of the Iberian Peninsula.

#### Microphthalmus sczelkowii

Metschnikow, 1865

##### Notes

Reported from Greece by Dando et al. (1995). In the Mediterranean also known from Egypt (Dorgham et al. 2013), otherwise distributed in the Black Sea, North Sea and East coast of North America.

#### Microphthalmus tyrrhenicus

Zunareli-Vandini, 1967

##### Notes

New record for Greece. One specimen, Elafonisi, Crete, 35°16'20.7"N, 23°32'15.9"E, 1 m depth, fine sand, collected in the framework of the MEDCORE project (unpublished data from Papageorgiou et al. 2006). Literature used for identification: Zunarelli (1967), Sordino (1990). Type locality: Mediterranean (Leghorn coast, Italy).

#### Neogyptis mediterranea

(Pleijel, 1993)

Gyptis mediterranea Pleijel, 1993

##### Notes

Reported from Greece by Chatzigeorgiou et al. (2016). Type locality: Mediterranean (Banyuls-sur-Mer, France).

#### Nereimyra punctata

(Müller, 1788)

##### Notes

Questionable status. Pleijel et al. (2012) consider the status of the species in the Mediterranean doubtful, as no specimens could be recovered from typical habitats despite intense sampling. Based on examination of other specimens, the authors conclude that the species is probably restricted to boreal regions. Records from the Adriatic and the Sea of Marmara are likewise considered questionable by Mikac (2015) and Çınar et al. (2014) respectively.

#### Oxydromus agilis

(Ehlers, 1864)

Ophiodromus agilis (Ehlers, 1864)

##### Notes

Questionable status. Reported from Greece by Nicolaidou et al. (1990). Type locality: Mediterranean (Martinscica, Adriatic). While it is possible that the species occurs in Greece, it has so far only been reported in grey literature and has not been found in over three decades, therefore its presence in Greece is here considered questionable.

#### Oxydromus flexuosus

(Delle Chiaje, 1827)

Ophiodromus flexuosus (Delle Chiaje, 1827)

##### Notes

Type locality: Mediterranean (Gulf of Naples).

#### Oxydromus pallidus

Claparède, 1864

Ophiodromus pallidus (Claparède, 1864) | *Podarke pallida* (Claparède, 1864)

##### Notes

Type locality: Mediterranean (Port-Vendres, France).

#### Podarkeopsis capensis

(Day, 1963)

Gyptis capensis (Day, 1963) | *Podarkeopsis capensis* (Day, 1963)

##### Notes

May have been confused with *Podarkeopsis galangaui* Laubier 1961 or *Podarkeopsis arenicolus* (La Greca, 1946), both native to the area but not included in any keys for the region and therefore probably underreported (Pleijel 2005). Reported from the Mediterranean, European Atlantic coasts and South Africa, considered questionable in Turkey by Çınar et al. (2014).

#### Podarkeopsis galangaui

Laubier, 1961

##### Notes

Reported from Greece by Arvanitidis (1994) based on a single specimen. Type locality: Mediterranean (Banyuls-sur-Mer, France). Pleijel (1998) considers the species a junior synonym of *Podarkeopsis arenicolus* (La Greca, 1946) but does not provide details and does not formalise the synonymy. As Rizzo and Salazar-Vallejo (2014) list both species in their key and recognise difference in the size of the eyes, length of the median antenna and chaetal details, the species is therefore treated as valid here, pending further information.

#### Psamathe fusca

Johnston, 1836

Kefersteinia cirrhata (Keferstein, 1862)

#### Syllidia armata

Quatrefages, 1866

Magalia perarmata Marion & Bobretzky, 1874 | *Syllidia armata* Quatrefages, 1866

### Iospilidae Bergström, 1914

#### Phalacrophorus pictus

Greeff, 1879

##### Notes

Questionable status. Reported from Greece by Antoniadou and Chintiroglou (2005) from a benthic sample of hard substrates at 3–15 m depth collected via SCUBA diving. No description or further information was provided for the taxon. Iospilidae are holopelagic and easily confused with benthic juvenile forms of other species (Jiméz-Cueto et al. 2006). Thus the record is considered doubtful here.

### Lacydoniidae Bergström, 1914

#### Lacydonia laureci

Laubier, 1975

##### Notes

Originally described from the Hellenic Trench (Matapan deep; 35°49'48"N, 22°20'42"E; 4690 m depth), no other records from Greece.

#### Lacydonia miranda

Marion & Bobretzky, 1875

##### Notes

Type locality: Mediterranean (Gulf of Marseille).

### Longosomatidae Hartman, 1944

#### Heterospio mediterranea

Laubier, Picard & Ramos, 1973

##### Notes

Type locality: Mediterranean (Algiers Bay).

### Lopadorrhynchidae Claparède, 1870

#### Lopadorrhynchus appendiculatus

Southern, 1909

##### Notes

Reported from Greece by Wesenberg-Lund (1939a). Widely distributed in the Mediterranean (Wesenberg-Lund 1939a).

#### Lopadorrhynchus brevis

Grube, 1855

##### Notes

Reported from Greece by Wesenberg-Lund (1939a); three additional specimens in the Senckenberg collection management system (Cat. No. 11278, 34°26'41.4"N, 26°06'34.2"E, 4255–4308 m; Cat. No. 11279, 35°48'57.6"N, 25°15'46.8"E, 1875–1877 m, Cat. No. 11280, 35°50'23.4"N, 25°16'08.4"E, 1876 m, all det. D. Fiege). Type locality: Mediterranean.

#### Lopadorrhynchus krohnii

(Claparède, 1870)

##### Notes

Reported from Greece by Wesenberg-Lund (1939a); one additional specimen from Greece in the collections of the Natural History Museum London (NHM 1968.52, 37°49'02"N, 19°45'06"E, 0-500 m depth, det. N. Tebble). Type locality: Mediterranean (Gulf of Naples).

#### Lopadorrhynchus nationalis

Reibisch, 1893

##### Notes

Reported from Greece by Wesenberg-Lund (1939a). *Lopadorrhynchus nationalis* is often listed in the literature as a synonym of *Lopadorrhynchus brevis* Grube, 1855, but Dales and Peter (1972) point out differences between the two species and consider them distinct. Widely distributed in the Mediterranean (Wesenberg-Lund 1939a).

#### Lopadorrhynchus uncinatus

Fauvel, 1915

##### Notes

Reported from Greece by Wesenberg-Lund (1939a). In the Mediterranean also known from Italy (Castelli et al. 2008) and the Adriatic (Mikac 2015), otherwise cosmopolitan in pelagic waters (Dales and Peter 1972).

#### Maupasia coeca

Viguier, 1886

##### Notes

Reported from Greece by NCMR (1989). Type locality: Mediterranean (Bay of Algiers).

#### Pelagobia longicirrata

Greeff, 1879

##### Notes

One specimen from Greece in the collections of the Senckenberg Museum (SMF 11264, 35°50'40.8"N, 22°19'49.8"E, 4754–4766 m, coll. date 1993-05-25, det. D. Fiege). In the Mediterranean also known from Italy (Castelli et al. 2008) and the Adriatic (Mikac 2015). Present in the adjacent Sea of Marmara (Çınar et al. 2014). Otherwise cosmopolitan in pelagic waters (Dales and Peter 1972).

### Lumbrineridae Schmarda, 1861

#### Gallardoneris iberica

Martins, Carrera-Parra, Quintino & Rodrigues, 2012

##### Notes

Reported from Greece by Katsiaras et al. (2017). In the Mediterranean also known from the central and western basins (Bertasi et al. 2014, Gómez et al. 2015, Mikac 2015, D’Alessandro et al. 2016). Its presence in the region was probably overlooked until its description.

#### Hilbigneris gracilis

(Ehlers, 1868)

Hilbigneris gracilis (Ehlers, 1868) | *Lumbriconereis gracilis* Ehlers, 1868 | *Lumbrineris gracilis* (Ehlers, 1868)

##### Notes

Type locality: Mediterranean (Adriatic).

#### Lumbricalus adriatica

(Fauvel, 1940)

Lumbricalus adriatica (Fauvel, 1940) | *Lumbrineris adriatica* (Fauvel, 1940)

##### Notes

Type locality: Mediterranean (Adriatic). Carrera-Parra (2004) redefined the genus, including *Lumbricalus adriatica*, using the maxillary apparatus in combination with chaetal types for differentiating species.

#### Lumbrinerides acuta

(Verrill, 1875) sensu Ramos, 1976

Lumbrineris acuta Verrill, 1875 | *Lumbrinerides acuta* (Verrill, 1875)

##### Notes

Questionable status. Reported from Greece by Dounas (1986). The record refers to *Lumbrinerides acuta* sensu Ramos 1976. The original description by Verrill (1875) is not detailed, but the redescription by Perkins (1979) based on specimens collected in the type locality (Rhode Island, USA) includes an accessory tooth on maxillary pair MI. The absence of an accessory tooth, as it is described in Ramos (1976), may indicate an undescribed species (Gómez et al. 2015). In the Mediterranean also known from Spain (Sardá et al. 1999), Italy (Castelli et al. 2008), Turkey (Çınar et al. 2014) and the Adriatic (Mikac 2015), but it is unclear whether these records refer to specimens having an accessory tooth or not. While it is possible that the species occurs in Greece, it has so far only been reported in grey literature and has not been found in over three decades, therefore its presence in Greece is here considered questionable.

#### Lumbrinerides amoureuxi

Miura, 1980

##### Notes

In the Mediterranean known from Greece, Turkey (Çınar et al. 2014) and Cyprus (Çınar 2005), otherwise distributed along the Atlantic coast of Europe.

#### Lumbrinerides carpinei

(Ramos, 1976)

##### Notes

New record for Greece. Two specimens, Pachia Ammos, Crete, 35°06'39.6"N, 25°48'32.4"E, 1–5 m depth, fine to coarse sand, collected in the framework of the MEDCORE project (unpublished data from Papageorgiou et al. 2006). Literature used for identification: Ramos (1976). Type locality: Western Mediterranean.

#### Lumbrinerides laubieri

Miura, 1980

##### Notes

In the Mediterranean only reported from Greece (Simboura and Zenetos 2005). Known from deep waters off the Atlantic coast of Europe.

#### Lumbrinerides neogesae

Miura, 1981

##### Ecological interactions

###### Native status

Non-native (casual)

##### Notes

New record for Greece. One specimen, Elafonisi, Crete, 35°16'20.7"N, 23°32'15.9"E, 1 m depth, fine sand, collected in the framework of the MEDCORE project (unpublished data from Papageorgiou et al. 2006). Literature used for identification: Miura (1980), Gravina and Cantone (1991). In the Mediterranean also known from Italy (Gravina and Cantone 1991). Originally described from South Africa.

#### Lumbrineriopsis paradoxa

(Saint-Joseph, 1888)

Lumbriconereis paradoxa Saint-Joseph, 1888 | *Lumbrineriopsis paradoxa* (Saint-Joseph, 1888) | *Lumbrineris paradoxa* Saint-Joseph, 1888

#### Lumbrineris coccinea

(Renier, 1804)

Lumbriconereis coccinea (Renier, 1804)| *Lumbrineris coccinea* (Renier, 1804)

##### Notes

Type locality: Mediterranean. Carrera-Parra (2006) considers records from outside the Mediterranean Sea doubtful.

#### Lumbrineris inflata

Moore, 1911

##### Notes

Questionable status. Reported from Greece by Arvanitidis (2000a) and Kitsos (2003); the first Mediterranean record was by Giangrande et al. (1981). Arvanitidis (2000a) argues that the species may have been underreported in the Mediterranean due to its similarity with *Lumbrineris coccinea* (Renier, 1804). Carrera-Parra (2006) and D’Alessandro et al. (2016) consider Mediterranean records of the species doubtful based on its otherwise East Pacific distribution. Çınar (2009) argues that the description and illustrations of *Lumbrineris inflata* by Giangrande et al. (1981) fit the characters of *Lumbrineris perkinsi* Carrera-Parra, 2001. *Lumbrineris inflata* differs from *Lumbrineris perkinsi* mainly in the number of teeth of maxilla III (M III). Giangrande et al. (1981), as well as Arvanitidis (2000a) for Greece, report a maxillary formula which could correspond to *Lumbrineris perkinsi* ("M III with 3–4 teeth"). Although published descriptions and records from the Mediterranean indicate the presence of *Lumbrineris perkinsi*, re-examination of all the material is needed to draw conclusions about the identity of the Greek records.

#### Lumbrineris latreilli

Audouin & Milne Edwards, 1834

Lumbriconereis latreilli Audouin & Milne Edwards, 1834 | *Lumbrineris latreilli* Audouin & Milne Edwards, 1834

##### Notes

Frequently reported from Greece, however, mainly on the basis of external and unreliable diagnostic characters. Species descriptions focusing on the maxillary apparatus and other unambiguous and size-independent characters have recently become available (Carrera-Parra 2006, Martins et al. 2012, D’Alessandro et al. 2016). Therefore, re-examination of material could reduce the frequency of records and even reveal the presence of overlooked species. In the Mediterranean, the species is widely recorded (e.g. Campoy 1982, Çınar 2005, Castelli et al. 2008, Ayari et al. 2009, Çınar et al. 2014, Mikac 2015). Otherwise distributed in the North-East Atlantic; its presumed cosmopolitan distribution is questionable (Carrera-Parra 2006).

#### Lumbrineris luciliae

Martins, Carrera-Parra, Quintino & Rodrigues, 2012

##### Notes

New record for Greece. Specimens previously reported as as *Lumbrineris cingulata* Ehlers, 1897 by Chatzigeorgiou et al. (2016) from rocky shores in northern Crete were re-examined and found to belong to *Lumbrineris luciliae* as described by Martins et al. (2012), although the reported slight distal curvature of the aciculae could not be observed. In the Mediterranean also known from Italy (D’Alessandro et al. 2016). Otherwise known from the Atlantic coast of the Iberian Peninsula (Martins et al. 2012).

#### Lumbrineris nonatoi

Ramos, 1976

##### Notes

Type locality: Mediterranean (Baie de Rosas, Spain). *Lumbrineris nonatoi* has several diagnostic characters in common with the – later described – *Gallardoneris iberica* Martins, Carrera-Parra, Quintino & Rodrigues, 2012. Due to this confusion, Bertasi et al. (2014) and Katsiaras et al. (2017) suggest that several literature records of *Lumbrineris nonatoi* could belong to the overlooked *Gallardoneris iberica*, although the overall presence of the former in the region has not been doubted.

#### Ninoe armoricana

Glémarec, 1968

#### Ninoe nigripes

Verrill, 1873

Ninoe kinbergi Ehlers, 1887 | *Ninoe nigripes* Verrill, 1873

##### Notes

Questionable status. Reported from Greece as *Ninoe kinbergi* by Vamvakas (1970) and Vamvakas (1971). Arvanitidis (2000a) lists these records under the name *Ninoe nigripes*, following the synonymy established by Pettibone (1963). In the Mediterranean reported from the central basin as *Ninoe kinbergi* (Castelli et al. 2008) and as *Ninoe nigripes* by Mikac (2015) from the Adriatic Sea. Otherwise distributed in the West Atlantic.

#### Scoletoma emandibulata

(Pillai, 1961)

Lumbrineris emandibulata Pillai, 1961

##### Notes

Questionable status. Reported from Greece by Dando et al. (1995) and Akoumianaki (2004). The record by Akoumianaki (2004) actually refers to *Scoletoma emandibulata mabiti*, as Ramos 1976 is given as the authority; it remains unclear whether the same holds true for the specimen of Dando et al. (1995). Originally described from the Indian Ocean.

#### Scoletoma emandibulata mabiti

(Ramos, 1976)

Lumbrineris emandibulata mabiti Ramos, 1976

##### Notes

Type locality: Mediterranean (Catalan Coast).

#### Scoletoma fragilis

(O.F. Müller, 1776)

Lumbriconereis fragilis (O.F. Müller, 1776) | *Lumbrineris fragilis* (O.F. Müller, 1776) | *Scoletoma fragilis* (O.F. Müller, 1776)

##### Notes

Species complex. Frequently reported from Greece and other parts of the Mediterranean (Castelli et al. 2008, Ayari et al. 2009, Çınar et al. 2014, Mikac 2015). Otherwise distributed in the Atlantic Ocean, Arctic Ocean and North Sea. However, molecular analyses revealed the existence of at least two putative cryptic species, one in the Atlantic and one in the Arctic Ocean (Carr et al. 2011).

#### Scoletoma funchalensis

(Kinberg, 1865)

Lumbriconereis funchalensis Kinberg, 1865 | *Lumbrineris funchalensis* (Kinberg, 1865) | *Scoletoma funchalensis* (Kinberg, 1865)

##### Notes

In the Mediterranean also known from the western and central basins (Campoy 1982, Castelli et al. 2008), Tunisia (Ayari et al. 2009), Turkey (Çınar et al. 2014) and the Adriatic (Mikac 2015). Otherwise distributed in the North-East Atlantic. However, Oug (2011) noted that *Scoletoma funchalensis* is poorly known and the described characters by Fauvel (1923) could be confused with juveniles of several species.

#### Scoletoma impatiens

(Claparède, 1868)

Lumbriconereis impatiens (Claparède, 1868) | *Lumbrineris impatiens* Claparède, 1868 | *Scoletoma impatiens* (Claparède, 1868)

##### Notes

Type locality: Mediterranean (Gulf of Naples). George and Hartmann-Schröder (1985) synonymised *Scoletoma impatiens* with the South African *Scoletoma tetraura* (Schmarda, 1861), but several authors do not accept this synonymy (see also notes under *Scoletoma tetraura*).

#### Scoletoma rovignensis

(Fauvel, 1940)

Lumbrineris rovignensis Fauvel, 1940 | *Scoletoma rovignensis* (Fauvel, 1940)

##### Notes

Questionable status. Reported from Greece by Dounas (1986). Type locality: Mediterranean (Adriatic). While it is possible that the species occurs in Greece, it has so far only been reported in grey literature and has not been found in over three decades, therefore its presence in Greece is here considered questionable.

#### Scoletoma tetraura

(Schmarda, 1861)

Lumbrineris tetraura (Schmarda, 1861) | *Scoletoma tetraura* (Schmarda, 1861)

##### Notes

Questionable status. Reported from Greece by Koukouras and Rousso (1991). In the Mediterranean also known from Turkey (Doğan et al. 2005; but considered questionable by Çınar et al. 2014), Cyprus (Çınar 2005), Italy (Castelli et al. 2008), Albania (Marzano et al. 2010) and the Adriatic (listed under *Scoletoma impatiens* (Claparède, 1968) by Mikac (2015)). Originally described from South Africa. George and Hartmann-Schröder (1985) synonymised the European species *Scoletoma impatiens* with *Scoletoma tetraura*. However, several authors question the presence of the latter in Europe and suggest using the name *Scoletoma impatiens* for specimens from the area until a more detailed revision becomes available (Martins et al. 2012, Çınar et al. 2014, Gómez et al. 2015, Mikac 2015, D’Alessandro et al. 2016). The main diagnostic character of the genus can be problematic, since several juvenile specimens of *Lumbrineris* and *Hilbigneris* species may lack composite hooded hooks and can be key out as *Scoletoma* (Oug 2011).

### Magelonidae Cunningham & Ramage, 1888

#### Magelona alleni

Wilson, 1958

##### Notes

Reported from Greece by Dounas (1986) and Arvanitidis (1994). In the Mediterranean also known from France (Fiege et al. 2000b), Italy (Castelli et al. 2008), Turkey (Çınar et al. 2014) and the Adriatic (Mikac 2015). Probably underreported, since older records of *Magelona cincta* Ehlers, 1908 may belong to *Magelona alleni* (Wilson 1958; see also notes under *Magelona cincta*).

#### Magelona cincta

Ehlers, 1908

##### Notes

Questionable status. Reported from Greece by Zarkanellas (1980) and Makra and Nicolaidou (2000), but specimens probably belong to *Magelona alleni* Wilson, 1958. Wilson (1958) described *Magelona alleni* from material misidentified as *Magelona cincta* and concluded that the latter does not occur in European coasts.

#### Magelona equilamellae

Harmelin, 1964

##### Notes

Type locality: Mediterranean (Villefranche-sur-Mer, France).

#### Magelona filiformis

Wilson, 1959

#### Magelona minuta

Eliason, 1962

#### Magelona mirabilis

(Johnston, 1865)

##### Notes

Probably an underreported species, since older records of *Magelona papillicornis* F. Müller, 1858 may belong to *Magelona mirabilis* (Fiege et al. 2000b; see also notes under *Magelona papillicornis*)

#### Magelona papillicornis

F. Müller, 1858

##### Notes

Questionable status. Until 1977, all European magelonids with mucronate chaetae on chaetiger 9 were assigned to *Magelona papillicornis*, originally described from Brazil. However, Jones (1977) re-described *Magelona papillicornis* and clarified that it actually lacks this character and therefore questioned its presence in European waters. Fiege et al. (2000b) demonstrated the presence of two species bearing mucronate chaetae on chaetiger 9 in Europe; *Magelona mirabilis* (Johnston, 1865) and *Magelona johnstoni* Fiege, Licher & Mackie, 2000. Greek records of *Magelona papillicornis* could belong to either of these, although only *Magelona mirabilis* has been recorded up to now.

#### Magelona wilsoni

Glémarec, 1966

##### Notes

One specimen from Greece in the collections of the Senckenberg Museum (SMF 11286, 39°15'0.6"N, 23°42'32.4"E, 1243 m, coll. date 1998-01-01, det. D. Fiege). In the Mediterranean also known from the coasts of France and Spain (Labrune et al. 2007, Serrano-Samaniego 2012) and the Adriatic (Mikac 2015).

### Maldanidae Malmgren, 1867

#### Axiothella constricta

(Claparède, 1869)

##### Notes

Type locality: Mediterranean (Gulf of Naples).

#### Axiothella rubrocincta

(Johnson, 1901)

##### Notes

Questionable status. Species complex. In the Mediterranean only reported from Greece (Dando et al. 1995). Distributed along the Pacific coast of North America where at least two morphologically indistinguishable forms with different life histories occur (Wilson 1983).

#### Chirimia biceps

(M. Sars, 1861)

Asychis biceps (M. Sars, 1861) | *Chirimia biceps* (M. Sars, 1861) | *Chirimia biceps biceps* (Sars, 1861)

#### Clymenella cf. koellikeri

(McIntosh, 1885)

##### Notes

In the Mediterranean only reported from Greece (Simboura 1996). The specimens differ from *Clymenella torquata* (Leidy 1855) (originally from the East coast of North America but occurring non-natively on the Atlantic coast of Europe) in the shape and number of acicular chaetae and from *Clymenella cincta* (Saint-Joseph, 1894) (occurring in the Mediterranean) in the shape of the collar and of the nuchal organs. The Greek specimens are most similar to the Pacific species *Clymenella koellikeri*, from which they differ in the following characters: Nuchal slits of *Clymenella koellikeri* reach up to the middle of the cephalic plate (until the lateral notches in the Greek material) and the rim or the collar of the 4^th^ chaetiger is smooth or slightly wavy in *Clymenella koellikeri*, whereas one Greek specimen shows a small lateral recess at the rim of the collar (Simboura 1996). The species is found regularly in Greece but is usually reported at genus level due to its uncertain identity. It may be an overlooked or cryptic species.

#### Euclymene collaris

(Claparède, 1869)

Clymene collaris (Claparède, 1869) | *Euclymene collaris* (Claparède, 1869)

##### Notes

Type locality: Mediterranean (Gulf of Naples).

#### Euclymene lombricoides

(Quatrefages, 1866)

Clymene lombricoides Quatrefages, 1866 | *Euclymene lombricoides* (Quatrefages, 1866)

#### Euclymene oerstedii

(Claparède, 1863)

Clymene oerstedii Claparède, 1863 | *Euclymene oerstedii* (Claparède, 1863)

#### Euclymene palermitana

(Grube, 1840)

Clymene palermitana Grube, 1840 | *Euclymene palermitana* (Grube, 1840) | *Praxillella palermitana* (Grube, 1840)

##### Notes

Type locality: Mediterranean (Palermo, Sicily, Italy).

#### Heteroclymene robusta

Arwidsson, 1906

Clymene robusta (Arwidsson, 1906) | *Euclymene robusta* (Arwidsson, 1906) | *Heteroclymene robusta* Arwidsson, 1906

#### Johnstonia clymenoides

Quatrefages, 1866

##### Notes

Reported from Greece by Bogdanos and Satsmadjis (1983) and Xenopoulou (1987). In the Mediterranean also known from France, Spain and Israel (Mackie and Gobin 1993), Egypt (Abd-Elnaby 2008), Turkey (Çınar et al. 2014) and the Adriatic (Mikac 2015), otherwise distributed along the European and North African Atlantic coasts (Mackie and Gobin 1993).

#### Leiochone leiopygos

(Grube, 1860)

Clymenura clypeata (Saint-Joseph, 1894) | *Leiochone clypeata* Saint-Joseph, 1894 | *Leiochone leiopygos* (Grube, 1860)

#### Lumbriclymene minor

Arwidsson, 1906

#### Macroclymene santandarensis

(Rioja, 1917)

Clymene santandarensis Rioja, 1917 | *Euclymene santandarensis* (Rioja, 1917) | *Macroclymene santandarensis* (Rioja, 1917)

#### Maldane capensis

(Day, 1961)

Asychis capensis Day, 1961 | *Maldane capensis* (Day, 1961)

##### Notes

Questionable status. In the Mediterranean only reported from Greece (Papadopoulos 1986). Known from South Africa.

#### Maldane glebifex

Grube, 1860

#### Maldane sarsi

Malmgren, 1865

#### Maldanella harai

(Izuka, 1902)

##### Notes

Questionable status. In the Mediterranean only reported from Greece (Bogdanos and Nicolaidou 1985, Bogdanos and Satsmadjis 1987, Nicolaidou and Papadopoulou 1989, Koulouri et al. 2015). Outside the Mediterranean reported from worldwide locations, mostly from bathyal and abyssal depths.

#### Metasychis gotoi

(Izuka, 1902)

Asychis gotoi (Izuka, 1902) | *Metasychis gotoi* (Izuka, 1902)

##### Ecological interactions

###### Native status

Non-native (established)

##### Notes

Originally distributed in the Red Sea, the Indian Ocean and the North Pacific; commonly reported from locations throughout the Mediterranean (Simboura and Zenetos 2005). The first Mediterranean record of *Metasychis gotoi* is from the Northern Adriatic (Fauvel 1940), constituting one of the earliest records of non-native species in the basin. As *Metasychis gotoi* is reported from worldwide distributions, it is likely that the name refers to a complex of cryptic species. The Mediterranean material could belong to an overlooked native species (J. Langeneck, pers. comm.).

#### Micromaldane ornithochaeta

Mesnil, 1897

##### Notes

Reported from Greece by Eleftheriou et al. (1990) and NCMR (2000a). In the Mediterranean also known from Israel (Ben-Eliahu 1976b), Spain (Alós 1990), Italy (Gherardi et al. 2002), Egypt (Abd-Elnaby 2008) and the Adriatic (Mikac 2015). Present in the adjacent Sea of Marmara (Çınar et al. 2014). Otherwise distributed along the European Atlantic coasts (Gherardi et al. 2002).

#### Nicomache lumbricalis

(Fabricius, 1780)

##### Notes

Questionable status. Frequently reported from Greece and other Eastern Mediterranean locations; however, de Assis et al. (2007) in their review of the genus restrict its distribution to boreal regions.

#### Nicomache maculata

Arwidsson, 1911

##### Notes

Questionable status. In the Mediterranean only reported from Greece (e.g. Bogdanos and Satsmadjis 1983, Eleftheriou et al. 1990, Tselepides 1992, Simboura 1996; full reference list in Suppl. material 2), de Assis et al. (2007) restrict its distribution to the Shetland Islands.

#### Nicomache trispinata

Arwidsson, 1906

##### Notes

Reported from Greece by Tselepides (1992). In the Mediterranean also known from France (Clausade 1969), Israel (Ben-Eliahu 1995) and Italy (Castelli et al. 2008), otherwise distributed along the Atlantic coasts of Europe and in the North Sea.

#### Petaloproctus terricolus

Quatrefages, 1866

#### Praxillella affinis

(M. Sars in G.O. Sars, 1872)

Clymene affinis M. Sars in G.O. Sars, 1872 | *Praxillella affinis* (M. Sars in G.O. Sars, 1872)

#### Praxillella gracilis

(M. Sars, 1861)

Clymene gracilis Sars, 1861| *Praxillella gracilis* (M. Sars, 1861)

#### Praxillella lophoseta

(Orlandi, 1898)

Clymene lophosetosa Orlandi, 1898 | *Praxillella lophoseta* (Orlandi, 1898)

##### Notes

Type locality: Mediterranean (Gulf of Naples).

#### Praxillella praetermissa

(Malmgren, 1865)

##### Notes

Species complex. At least three putative cryptic species exist, one each in the Arctic and West Atlantic (Carr et al. 2011) and another in Portugal (Lobo et al. 2016).

#### Praxillura longissima

Arwidsson, 1906

##### Notes

Questionable status. In the Mediterranean only reported from Greece (Eleftheriou et al. 1990). Commonly distributed in the North-East Atlantic.

#### Rhodine gracilior

Tauber, 1879

#### Rhodine loveni

Malmgren, 1865

### Myzostomatidae Benham, 1896

#### Myzostoma cirriferum

Leuckart, 1836

##### Notes

Reported from Greece by Chatzigeorgiou et al. (2016) based on a single specimen. In the Mediterranean also known from Tunisia (Wesenberg-Lund 1939b), France (Laubier and Paris 1962), Italy (Castelli et al. 2008) and the Adriatic (Mikac 2015), otherwise distributed along the Atlantic coasts of Europe (Nygren and Pleijel 2015).

### Nephtyidae Grube, 1850

#### Aglaophamus agilis

(Langerhans, 1880)

Aglaophamus rubella (Michaelsen, 1897) | *Nephthys agilis* Langerhans, 1880 | *Nephthys rubella* Michaelsen, 1897

#### Aglaophamus malmgreni

(Théel, 1879)

Aglaophamus malmgreni (Théel, 1879) | *Nephthys malmgreni* Théel, 1879

##### Notes

Questionable status. Reported from Greece by von Marenzeller (1902). Marenzeller's description differs from the current concept of *Aglaophamus malmgreni* in the first branchial chaetiger and the pharyngeal papillation. Marenzeller describes his specimens with branchiae starting at chaetigers 6–7 (in one specimen at chaetiger 10) and states that his specimens correspond to the description of *Nephtys malmgreni* by McIntosh (1900) (listed by McIntosh under the name *Nephtys longisetosa*) in the number of papillae. McIntosh describes 15 rows of 11–15 papillae. Branchial and papillation characters correspond to *Aglaophamus pulcher* (Rainer, 1991), but Marenzeller's material should be examined for confirmation. In the Mediterranean, *Aglaophamus malmgreni* has also been reported from the Adriatic Sea (Mikac 2015), otherwise it is distributed in circumpolar regions and records from southern Europe require confirmation (Ravara et al. 2010).

#### Aglaophamus pulcher

(Rainer, 1991)

Nephtys pulchra Rainer, 1991

##### Notes

Reported from Greece by Arvanitidis (2000a) but specimens of *Aglaophamus malmgreni* (Théel, 1897) by von Marenzeller (1902) could belong to *Aglaophamus pulcher* (see notes there). In the Mediterranean also known from abyssal canyons and plains in the western basin (Ravara et al. 2010), otherwise distributed in the North East Atlantic. May have been confused in the past with *Nephtys hystricis* McIntosh, 1900 and *Nephtys incisa* Malmgren 1865 (Rainer 1991) and therefore been overlooked in the area.

#### Inermonephtys inermis

(Ehlers, 1887)

Aglaophamus inermis (Ehlers, 1887) | *Inermonephtys inermis* (Ehlers, 1887)| *Nephtys inermis* Ehlers, 1887

##### Notes

Questionable status. Ravara et al. (2010) found morphological differences between European specimens identified as *Inermonephtys inermis* and the holotype from Florida and established a new species, *Inermonephtys foretmontardoi* Ravara, Cunha & Pleijel, 2010 for the European populations. *Inermonephtys inermis* is restricted to the West Atlantic. It is highly likely that Greek specimens of *Inermonephtys inermis* belong to *Inermonephtys foretmontardoi*, but no specimens have been re-examined yet for confirmation.

#### Micronephthys sphaerocirrata

(Wesenberg-Lund, 1949)

Micronephthys sphaerocirrata (Wesenberg-Lund, 1949)| *Nephthys sphaerocirrata* Wesenberg-Lund, 1949

##### Notes

Ravara et al. (2010) regard the presence of the species in the Mediterranean as doubtful due to its otherwise Indo-Pacific distribution. Greek specimens may also have been confused with *Micronephthys stammeri* (Augener, 1932) in the past.

#### Micronephthys stammeri

(Augener, 1932)

Micronephthys maryae San Martín, 1982| *Micronephthys stammeri* (Augener, 1932)

##### Notes

Type locality: Mediterranean (*Micronephthys maryae*: Ibiza, *Micronephthys stammeri*: Adriatic).

#### Nephtys assimilis

Ørsted, 1843

##### Notes

Rarely reported from the Mediterranean, but was considered a synonym of *Nephtys hombergii* Savigny in Lamarck, 1818 until its resurrection and redescription by Rainer (1989) and could therefore be underreported.

#### Nephtys caeca

(Fabricius, 1780)

##### Notes

Ravara et al. (2010) re-examined material from the Mediterranean and identified it as *Nephtys caeca*, concluding that, although the species is commonly distributed in Arctic waters, it occasionally occurs in warmer waters of the Mediterranean as far as the Black Sea.

#### Nephtys ciliata

(Müller, 1788)

##### Notes

Questionable status. Reported from Greece by Vamvakopoulou (1991). In the Mediterranean also reported from the western basin (Fredj 1974) and the Adriatic (Mikac 2015). Commonly distributed in boreal areas; Mediterranean records require confirmation (Ravara et al. 2010).

#### Nephtys cirrosa

Ehlers, 1868

#### Nephtys hombergii

Savigny in Lamarck, 1818

##### Notes

Type locality: Mediterranean (Gulf of Naples).

#### Nephtys hystricis

McIntosh, 1900

##### Notes

The description of *Nephtys hystricis* by Fauvel (1923) actually refers to *Nephtys incisa* Malmgren, 1865 and vice versa (Rainer 1990). Thus, specimens identified as *Nephtys hystricis* using Fauvel's key and description probably belong to *Nephtys incisa*. Both *Nephtys incisa* and *Nephtys hystricis* have been reported from Greece, thus they can both be considered as present, despite this confusion.

#### Nephtys incisa

Malmgren, 1865

##### Notes

See notes under *Nephtys hystricis* McIntosh, 1900.

### Nereididae Blainville, 1818

#### Alitta succinea

(Leuckart, 1847)

Neanthes succinea (Leuckart, 1847) | *Nereis succinea* Leuckart, 1847

##### Notes

Fauvel (1923) considered *Nereis lamellosa* Ehlers 1868 a synonym of *Nereis succinea* but revoked this synonymy in a subsequent publication, describing the differentiating characters (Fauvel 1936b). Later authors (e.g. Day 1967, Campoy 1982) continued to treat the species as synonymous. Thus, many reports of *Alitta succinea* may actually contain specimens of *Nereis lamellosa*, which in turn is probably underreported in the Mediterranean (Arvanitidis 2000a).

#### Alitta virens

(M. Sars, 1835)

Neanthes virens (M. Sars, 1835)

##### Notes

Questionable status. Species complex. In the Mediterranean only reported from Greece (Christomanos and Giannitsis 1962). Distributed in the North Atlantic and North Pacific and comprising at least two cryptic species with genetic and morphological differences in these two areas (Carr et al. 2011).

#### Ceratonereis (Composetia) costae

(Grube, 1840)

Ceratonereis (Composetia) costae (Grube, 1840) | *Ceratonereis costae* (Grube, 1840) | *Nereis costae* Grube, 1840 | Nereis (Ceratonereis) costae Grube, 1840

##### Notes

Type locality: Mediterranean.

#### Ceratonereis (Composetia) hircinicola

(Eisig, 1870)

Ceratonereis hircinicola (Eisig, 1870) | *Nereis hircinicola* (Eisig, 1870)

##### Notes

Type locality: Mediterranean.

#### Ceratonereis (Composetia) vittata

Langerhans, 1884

##### Notes

In the Mediterranean only reported from Greece (Maidanou et al. 2017). Originally described from Madeira; possibly underreported due to confusion with *Nereis rava* (Ehlers, 1864) or *Ceratonereis hircinicola* (Eisig, 1870) (Núñez and Brito 2002).

#### Eunereis longissima

(Johnston, 1840)

##### Notes

Reported from Greece by Arvanitidis (2000a) and Chatzigeorgiou et al. (2016). In the Mediterranean also known from France (Picard 1965), Spain (Núñez 2004), Italy (Castelli et al. 2008), Turkey (Çınar et al. 2014) and the Adriatic (Mikac 2015). Otherwise distributed along the Atlantic coasts of Europe.

#### Hediste diversicolor

(O.F. Müller, 1776)

Hediste diversicolor (O.F. Müller, 1776) | Nereis (Hediste) diversicolor Müller, 1776 | *Nereis diversicolor* Müller, 1776

##### Notes

Species complex. Multiple genetic lineages exist in Europe (Nygren 2014, Lobo et al. 2016) which could correspond to cryptic species. Vasileiadou et al. (2016) found genetically highly isolated populations with unique haplotypes in Greek lagoons.

#### Leonnates persicus

Wesenberg-Lund, 1949

##### Ecological interactions

###### Native status

Non-native (casual)

##### Notes

New record for Greece. Two specimens, Argolikos Gulf, 37°33'34.9''N, 22°47'06''E, 16 m depth, sandy silt with biogenic detritus, collected during monitoring for the Water Framework Directive. Literature used for identification: Çınar et al. (2002). An Indo-Pacific species, in the Mediterranean also known from Israel (Ben-Eliahu 1991b) and Turkey (Çınar et al. 2002).

#### Micronereis variegata

Claparède, 1863

#### Neanthes acuminata

(Ehlers, 1868)

Neanthes caudata (sensu Delle Chiaje, 1827) | *Nereis caudata* (sensu Delle Chiaje, 1827)

##### Notes

Species complex. Reish et al. (2014) recovered four different putative cryptic species of *Neanthes acuminata* from worldwide locations (based on specimens traditionally referred to the species *Neanthes acuminata*, *Neanthes caudata*, and *Neanthes arenaceodentata* Moore, 1903, all of which are morphologically indistinguishable but could not be recovered by the analyses). From Europe, only specimens from Portugal were included in the analyses and it is currently unknown whether more cryptic species exist. Concerning nomenclature, *Neanthes acuminata* sensu lato should be used to refer to the species complex (Reish et al. 2014). While the name *Neanthes caudata* is often found in literature for the European species, it is not an available name. Delle Chiaje (1825) wrongly applied the name *Spio caudatus* Lamarck, 1818 to a nereidid, thus the name is based on a misidentification. The next available name is *Neanthes acuminata* (see detailed notes by G. Read for *S. caudatus* Lamarck, 1818, *Spio caudatus* sensu Delle Chiaje, 1827 and *N. caudata* (sensu Delle Chiaje, 1827)), a species described from the Gulf of Naples.

#### Neanthes agulhana

(Day, 1963)

##### Ecological interactions

###### Native status

Non-native (casual)

##### Notes

Reported from Greece by Chatzigeorgiou et al. (2016). Originally from South Africa, in the Mediterranean also reported from Spain (Núñez 2004) and Italy (Occhipinti-Ambrogi et al. 2011).

#### Neanthes fucata

(Savigny in Lamarck, 1818)

##### Notes

Reported from Greece by Keklikoglou et al. (2013). Type locality: Eastern Mediterranean.

#### Neanthes kerguelensis

(McIntosh, 1885)

Neanthes kerguelensis (McIntosh, 1885) | *Nereis kerguelensis* McIntosh, 1885

#### Neanthes nubila

(Savigny, 1822)

Neanthes irrorata (Malmgren, 1867) | *Neanthes nubila* (Savigny, 1822) | *Nereis irrorata* (Malmgren, 1867)

#### Nereis falsa

Quatrefages, 1866

Nereis falsa Quatrefages, 1866 | *Nereis splendida* Grube, 1840

##### Notes

*Nereis falsa* is considered a synonym of *Nereis splendida* Grube, 1840 by Núñez (2004). However, a *Nereis splendida* Blainville, 1825 also exists in literature and in online databases (e.g. WoRMS) which should take priority over Grube's species and render the latter a junior homonym. Gravina et al. (2015) therefore use *Nereis falsa* as the valid name, as *Nereis splendida* Grube, although having priority over *Nereis falsa* Quatrefages, is pre-occupied. However, the identity of the species described by Blainville (1825) species is confused and early authors (e.g. Quatrefages 1866:434, Rathke 1843:172) consider it indeterminable and probably belonging to *Nephtys*. In addition, Blainville (1825) creates a confusing situation of synonyms by applying the name *Nereis splendida* to a specimen he had received from Dr. Leach under the (probably unpublished) name *Nereis clava* (p. 439) and in the same publication transfers *Hesione splendida* Savigny 1818 to *Nereis*, creating an unresolved synonymy with *Nereis splendida* (p. 443). Until this confusion is resolved, the name *Nereis falsa* is used here, following Gravina et al. (2015). See also Salazar-Vallejo et al. (2017) and discussions on the Annelida mailing list for extensive clarifications on the identity and nomenclatural problems associated with *Nereis falsa* and *Nereis splendida*.

#### Nereis lamellosa

Ehlers, 1864

##### Notes

Reported from Greece by Arvanitidis (2000a) and Keklikoglou et al. (2013). Type locality: Mediterranean (Adriatic). Probably underreported, see remarks under *Alitta succinea* (Leuckart, 1947).

#### Nereis pelagica

Linnaeus, 1758

##### Notes

Species complex. At least three putative cryptic species exist in the Arctic, the Atlantic and Portugal (Lobo et al. 2016).

#### Nereis perivisceralis

Claparède, 1868

##### Notes

Reported from Greece by Keklikoglou et al. (2013) and Chatzigeorgiou et al. (2016). Type locality: Mediterranean (Gulf of Naples). Considered a synonym of *Nereis falsa* Quatrefages 1866 by Fauvel (1923), thus it may be underreported and records of *Nereis falsa* from Greece may include specimens of *Nereis perivisceralis*.

#### Nereis pulsatoria

(Savigny, 1822)

Nereis pulsatoria (Savigny, 1822) | *Nereis zonata* Malmgren, 1867

##### Notes

The taxonomy and nomenclature of *Nereis pulsatoria* and *Nereis zonata* are confused. In literature, both *Nereis pulsatoria* (Savigny, 1822) and *Nereis pulsatoria* Audouin & Milne Edwards 1834 are used (the latter not as a new name but as a reference to Savigny's species, thus not a homonym). It is currently unclear whether these two actually refer to the same species. Fauvel (1923) considers *Nereis pulsatoria* Audouin & Milne Edwards 1834 a synonym of *Nereis zonata* but does not mention Savigny's species. Núñez (2004) synonymises *Nereis zonata* Malmgren 1867 and *Nereis pulsatoria* Audouin & Milne Edwards, 1834 with *Nereis pulsatoria* (Savigny 1822), but without any mention of studied material or reference to the original description. Gravina et al. (2015) point out that the description of *Nereis pulsatoria* by Núñez (2004) is inconsistent when compared to the corresponding illustrations and that the described species differs from *Nereis zonata* in chaetal and paragnath arrangement. Gravina et al. (2015) consider only Savigny's species valid and restrict it to the Atlantic, but the authors base this statement on the respective species entries in WoRMS, which have not been reviewed recently, contain partly incorrect information and may constitute database inconsistencies. If the species are indeed synonymous, *Nereis pulsatoria*, being the older name, should have priority over *Nereis zonata*. Until this confusion is resolved, the name *Nereis pulsatoria* is kept here and *Nereis zonata* considered its synonym. Specimens from Greece have both been reported under the name *Nereis zonata* and *Nereis pulsatoria* sensu Núñez (2004).

#### Nereis rava

Ehlers, 1864

##### Notes

Type locality: Mediterranean (Adriatic).

#### Perinereis cultrifera

(Grube, 1840)

##### Notes

Species complex. Multiple records from Greece exist, but *Perinereis cultrifera* constitutes a complex of species in the Mediterranean (Scaps et al. 2000, Maltagliati et al. 2001). Specimens from Greece could belong to any of these or another cryptic species. Type locality: Mediterranean (Gulf of Naples).

#### Perinereis oliveirae

(Horst, 1889)

##### Notes

Reported from Greece by Koukouras et al. (1985) and Simboura (1987). In the Mediterranean also known from France (Bellan 1964a), Spain (Núñez 2004) and Italy (Castelli et al. 2008), otherwise distributed along the Atlantic coasts of Europe and North Africa. Italian records are, however, considered questionable by Castelli et al. (2008) as the species is easily confused with *Perinereis rullieri* Pilato, 1974.

#### Perinereis tenuisetis

(Fauvel, 1915)

##### Notes

One specimen from Greece in the collections of the Senckenberg Museum (SMF 5609, 36°36'N, 21°34'E, 3848 m depth, coll. date 1993-05-22 / 1993-05-23 det. M.N. Ben-Eliahu). Mediterranean species, type locality Sicily.

#### Platynereis coccinea

(Delle Chiaje, 1822)

##### Notes

Reported from Greece by Chatzigeorgiou et al. (2016). Type locality: Mediterranean (Italy). Previous reports of the species by Arvanitidis (2000a) and Simboura and Nicolaidou (2001) are based on a record by Ergen (1987) from the Turkish Aegean coast.

#### Platynereis dumerilii

(Audouin & Milne Edwards, 1834)

##### Notes

Lobo et al. (2016) show that specimens from Portugal and Italy genetically diverge, indicating potentially more than one cryptic species in Europe (pending further corroboration through analysis of more specimens). One of the sibling species of *Platynereis dumerilii* occurring near CO_2_ vents in Ischia, Italy, was recently shown to correspond to *Platynereis massiliensis* (Moquin-Tandon, 1869) (Valvassori et al. 2015).

#### Pseudonereis anomala

Gravier, 1900

Nereis anomala (Gravier, 1900)| *Pseudonereis anomala* Gravier, 1900

##### Ecological interactions

###### Native status

Non-native (established)

##### Notes

Frequently reported from Greece. Originally from the Red Sea, in the Mediterranean also known from Egypt (Fauvel 1937), Israel (Ben-Eliahu 1989), Turkey (Çınar and Ergen 2005) and Italy (D’Alessandro et al. 2015).

#### Websterinereis glauca

(Claparède, 1870)

Leptonereis glauca (Claparède, 1870)| *Websterinereis glauca* (Claparède, 1870)

##### Notes

Type locality: Mediterranean (Gulf of Naples).

### Oenonidae Kinberg, 1865

#### Arabella coeca

Fauvel, 1940

##### Notes

Reported from Greece by Chatzigeorgiou et al. (2016) based on a single specimen. Type locality: Mediterranean (Adriatic).

#### Arabella geniculata

(Claparède, 1868)

##### Notes

Type locality: Mediterranean (Gulf of Naples).

#### Arabella iricolor

(Montagu, 1804)

##### Notes

Species complex. Frequently reported from Greece and other parts of the Mediterranean (Çınar 2005, Castelli et al. 2008, Ayari et al. 2009, Çınar et al. 2014, Mikac 2015) and considered cosmopolitan. May however constitute a species complex comprising two cryptic species or populations along the East Coast of North America, differing in the maxillary formula (Colbath 1989, Zanol and Ruta 2015).

#### Drilonereis filum

(Claparède, 1868)

Drilonereis filum (Claparède, 1868) | *Drilonereis macrocephala* Saint-Joseph, 1888

##### Notes

Type locality: Mediterranean (Gulf of Naples). Often considered cosmopolitan, but Helgason et al. (1990) note the possibility of more than one species being reported under *Drilonereis filum*, since a wide variation of characters is found in literature.

#### Notocirrus scoticus

McIntosh, 1869

#### Oligognathus bonelliae

Spengel, 1882

##### Ecological interactions

###### Parasite of

*Bonellia viridis* Rolando, 1822

##### Notes

*Oligognathus bonelliae* can be found in host bodies of the echiurid *Bonellia viridis*. Type locality: Mediterranean (Gulf of Naples).

### Onuphidae Kinberg, 1865

#### Aponuphis bilineata

(Baird, 1870)

Aponuphis bilineata (Baird, 1870) | *Hyalinoecia bilineata* Baird, 1870

##### Notes

Species complex. Two colour morphs exist (Arias and Paxton 2015a) and molecular evidence of sibling species within *Aponuphis bilineata* are provided by Borisova et al. (submitted).

#### Aponuphis brementi

(Fauvel, 1916)

Aponuphis brementi (Fauvel, 1916) | *Aponuphis fauveli* (Rioja, 1918) | *Hyalinoecia brementi* Fauvel, 1916

##### Notes

Species complex. Arias and Paxton (2015a) consider *Aponuphis fauveli* a junior synonym of *A. brementi*, thus literature records of *Aponuphis fauveli* are here assigned to *Aponuphis brementi*. However, molecular evidence of sibling species within *Aponuphis brementi* were found by Borisova et al. (submitted). Type locality: Mediterranean (Bay of Roquebrun, France).

#### Diopatra neapolitana

Delle Chiaje, 1841

Diopatra neapolitana Delle Chiaje, 1841 | *Diopatra neapolitana neapolitana* Delle Chiaje, 1841

##### Notes

*Diopatra neapolitana*, originally described from the Gulf of Naples, used to be the only *Diopatra* species recorded along European coasts. However, several recent publications have demonstrated that more *Diopatra* species (*Diopatra marocensis* Paxton, Fadlaoui & Lechapt, 1995, *Diopatra micrura* Pires, Paxton, Quintino & Rodrigues, 2010, *Diopatra biscayensis* Fauchald, Berke & Woodin, 2012) are present along European coasts (e.g. Fauchald et al. 2012, Arias and Paxton 2013), including the Turkish Aegean (Çınar et al. 2014). Therefore, at least some of the Greek specimens of *Diopatra neapolitana* could belong to other *Diopatra* species.

#### Hyalinoecia tubicola

(O.F. Müller, 1776)

##### Notes

Species complex. Zaâbi et al. (2015) present evidence of possible cryptic species within *Hyalinoecia tubicola* in Tunisia.

#### Nothria conchylega

(Sars, 1835)

Nothria conchylega (Sars, 1835) | *Onuphis conchylega* Sars, 1835

##### Notes

Species complex. Frequently reported from Greece and other parts of the Mediterranean (e.g. Çınar 2005, Castelli et al. 2008, Ayari et al. 2009, Çınar et al. 2014, Mikac 2015). Otherwise distributed in the Arctic, Atlantic, Indian and Pacific Oceans. However, many of these records could belong to other species. Budaeva and Paxton (2013) note that morphological variations of *Nothria* Malmgren, 1866 have been underestimated in earlier species descriptions, including those of *Nothria conchylega*. In addition, two putative cryptic species of *Nothria conchylega* were discovered in the Arctic and Pacific Ocean (Carr et al. 2011).

#### Onuphis eremita

Audouin & Milne Edwards, 1833

##### Notes

Species complex. Frequently reported from Greece and other parts of the Mediterranean (e.g. Campoy 1982, Castelli et al. 2008, Ayari et al. 2009, Çınar et al. 2014, Mikac 2015) and considered cosmopolitan. However, the taxonomic history of the species includes doubtful synonymies, vague descriptions and redescriptions based on material from worldwide locations (Arias and Paxton 2014). The latter authors redefine the diagnostic characters, designate a neotype and confine its distribution to the East Atlantic and the Western and Central Mediterranean. In addition, they re-instate and re-describe *Onuphis pancerii* Claparède, 1868 from the *Onuphis eremita* complex and designate a neotype from Italy. Re-examination of specimens is required to determine the status of records of *Onuphis eremita* and possible other species of the complex in Greece.

#### Paradiopatra calliopae

Arvanitidis & Koukouras, 1997

##### Notes

Originally described from Greece by Arvanitidis and Koukouras (1997), mainly based on having equal antennae instead of a longer median one in *Paradiopatra bihanica* Intes & Le Loeuff, 1975. Arvanitidis and Koukouras (1997) examined the holotype of *Paradiopatra bihanica* but found it to be dried out and the tips of the antennae broken off. Budaeva and Fauchald (2011) considered the holotype to be in good condition and with equal antennae, placing *Paradiopatra calliopae* into synonymy with *Paradiopatra bihanica*. However, Paxton and Arias (2016), after re-examining the holotype of *Paradiopatra bihanica* and specimens of *Paradiopatra calliopae*, re-instated the latter as a valid species and doubt the presence of *Paradiopatra bihanica* in the Mediterranean. See also notes under *Paradiopatra quadricuspis* (M. Sars in G.O. Sars, 1872).

#### Paradiopatra fragosa

(Ehlers, 1887)

Sarsonuphis fragosa (Ehlers, 1887)

##### Notes

Questionable status. Reported twice from Greece as Sarsonuphis cf. fragosa (Karakassis 1991, Tselepides 1992) and once as *Sarsonuphis fragosa* (Eleftheriou and Smith 2000). No other Mediterranean records, commonly distributed in the Caribbean Sea. The species is not included in any studies of *Paradiopatra* from southern Europe (e.g. Arvanitidis and Koukouras 1997, Arias and Paxton 2015b) and it is probably absent from the area. Literature records could belong to *Paradiopatra calliopae* Arvanitidis & Koukouras, 1997.

#### Paradiopatra lepta

(Chamberlin, 1919)

Onuphis lepta Chamberlin, 1919

##### Notes

Questionable status. Reported from Greece by Vamvakas (1971). Arias and Paxton (2015b) doubt the validity of records from European coasts and suggest that this species has been confused with *Paradiopatra bihanica*, Intes & Le Loeuff, 1975 (Mediterranean records now accepted as *Paradiopatra calliopae* Arvanitidis & Koukouras, 1997) or *Nothria maremontana* Andre & Pleijel, 1989. Arvanitidis and Koukouras (1997) note that, where descriptions are available for onuphids recorded in the Mediterranean under the name *Onuphis lepta*, these differ from the original *Onuphis lepta* and are much closer to *Paradiopatra calliopae*. Although re-examination of material is required, the distribution in the region is considered doubtful.

#### Paradiopatra quadricuspis

(M. Sars in G.O. Sars, 1872)

Paradiopatra quadricuspis (M. Sars in G.O. Sars, 1872) | *Sarsonuphis quadricuspis* (M. Sars in G.O. Sars, 1872)

##### Notes

Questionable status. Budaeva and Fauchald (2011) doubt the presence of the species in the Mediterranean and suggest that Greek records by Arvanitidis and Koukouras (1997) may represent a new species. Their argument of a limited distribution from Norway to Iceland has been accepted also by other authors (Mikac 2015, Arias and Paxton 2015b, but see Çınar et al. 2014). Simboura (1996) found specimens of *Sarsonuphis quadricuspis* from Greece to differ from the type material but also from French material described by Bellan (1963). She considered it an undescribed species designated as *Sarsonuphis* sp.; but later found it to match the description of *Paradiopatra calliopae* Arvanitidis & Koukouras, 1997. Re-examination of material is required to draw conclusions about the identity of the Greek records but the species' distribution in the region is doubtful.

#### Rhamphobrachium brevibrachiatum

(Ehlers, 1874)

Onuphis brevibranchiata (Ehlers, 1874) | *Rhamphobrachium brevibrachiatum* (Ehlers, 1874)

##### Notes

Reported from Greece by Simboura (1996). In the Mediterranean also known from the central and western basins (Bellan 1964a, Castelli et al. 2008) and the Adriatic (Mikac 2015); otherwise distributed in the North Atlantic and Japan.

### Opheliidae Malmgren, 1867

#### Armandia cirrhosa

Filippi, 1861

##### Notes

Type locality: Mediterranean (Sardinia).

#### Armandia polyophthalma

Kükenthal, 1887

##### Notes

Type locality: Mediterranean (Gulf of Naples).

#### Ophelia barquii

Fauvel, 1927

##### Notes

Reported from Greece by Papageorgiou et al. (2006). Type locality: Mediterranean (Agay, France). *Ophelia barquii* was initially described as a subspecies of *Ophelia radiata* (Delle Chiaje, 1828) by Fauvel (1927), who distinguished the European species of *Ophelia* (*Ophelia neglecta* Schneider, 1892, Savigny, 1822, *Ophelia radiata* (Delle Chiaje, 1828) and *Ophelia barquii*) on the basis of the number of gill pairs. This was regarded as an unreliable character by subsequent authors (e.g. Bellan 1964a) who considered *Ophelia radiata barquii* and *Ophelia radiata* synonyms of *Ophelia bicornis*. However, molecular and morphological analyses by Maltagliati et al. (2004) and Maltagliati et al. (2005) clearly differentiate *Ophelia barquii* from *Ophelia bicornis*, the constant morphological difference between the two species being the number of nephridiopore pairs (five in *Ophelia barquii*, six in *Ophelia bicornis*). See also comments under *Ophelia bicornis*.

#### Ophelia bicornis

Savigny in Lamarck, 1818

Ophelia bicornis Savigny in Lamarck, 1818 | *Ophelia radiata* (Delle Chiaje, 1828)

##### Notes

Species complex with a confused taxonomic history. Originally described from the Gulf of Naples. Fauvel (1927) considered *Ophelia bicornis* and *Ophelia radiata* as distinct based on the number of gill pairs (*Ophelia radiata*: 14 pairs; *Ophelia bicornis*: 15 pairs) and geographic distribution. However, both species and intermediate forms (with 14 gill pairs on one side and 15 on the other) have been found to co-occur by Britton-Davidian and Amoureux (1982), who also found genetic differences between the two forms in an Atlantic population, with asymmetric specimens belonging to either of the two genetic variants. Based on these findings, the authors consider the species distinct. However, they were not able to assign Mediterranean specimens to either of the two Atlantic forms. Maltagliati et al. (2005) consider the results inconclusive and refer to the complex as *Ophelia bicornis* sensu lato. Parapar (2012) studied individuals of both *Ophelia radiata* and *Ophelia bicornis* from the collections of the Natural History Museums in Copenhagen and Madrid and could not find any differences apart from the number of gill pairs — a character which he considered too unreliable for discrimination.

#### Ophelia limacina

(Rathke, 1843)

Ophelia borealis Quatrefages, 1866

##### Notes

Reported from Greece by Maidanou et al. (2017). In the Mediterranean also known from Israel (Ben-Eliahu 1995), Italy (Castelli et al. 2008), Turkey (Çınar et al. 2014) and the Adriatic (Mikac 2015), otherwise distributed in the boreal regions of Europe.

#### Ophelia roscoffensis

Augener, 1910

##### Notes

Reported from Greece by Simboura (1996), Zenetos et al. (1997) (based on the same specimens); Identification confirmed by G. Bellan (pers. comm. in Simboura 1996). In the Mediterranean also known from France (Bellan 1964a) and Turkey (Çınar et al. 2014), otherwise known from the Atlantic coasts of France, Spain and from the British Isles. The Greek specimens differ from Atlantic ones by a reduced number of branchial pairs (Simboura 1996).

#### Ophelina acuminata

Ørsted, 1843

Ammotrypane aulogaster Rathke, 1843 | *Ophelina acuminata* Ørsted, 1843 | *Ophelina aulogaster* (Rathke, 1843)

##### Notes

Species complex. Comprises at least two putative cryptic species, one in the Atlantic, one in the Arctic Ocean (Carr et al. 2011).

#### Ophelina cylindricaudata

(Hansen, 1879)

Ammotrypane cylindricaudatus Hansen, 1879 | *Ophelina cylindricaudata* (Hansen, 1879)

#### Polyophthalmus pictus

(Dujardin, 1839)

#### Tachytrypane jeffreysii

McIntosh, 1879

### Orbiniidae Hartman, 1942

#### Leitoscoloplos kerguelensis

(McIntosh, 1885)

##### Notes

Questionable status. Reported from Greece by Akoumianaki (2004). In the Mediterranean also reported (questionably) from Spain / France (Ramos 1976b). *Leitoscoloplos kerguelensis* is a widely reported but much confused species with several incorrect re-descriptions and many wrongly identified specimens (Mackie 1987). The species is probably restricted to the Kerguelen Islands area and the Mediterranean material belongs to *Leitoscoloplos kerguelensis* sensu Ramos, 1976 which constitutes an undescribed species (Blake 2017).

#### Naineris laevigata

(Grube, 1855)

##### Notes

Type locality: Mediterranean.

#### Naineris quadraticeps

Day, 1965

##### Ecological interactions

###### Native status

Non-native (questionable)

##### Notes

Questionable status. In the Mediterranean only reported from Greece (Harmelin 1969). Distributed in the Indo-Pacific; its occurrence in the Mediterranean is considered questionable (Zenetos et al. 2010).

#### Orbinia latreillii

(Audouin & Milne Edwards, 1833)

Orbinia latreillii (Audouin & Milne Edwards, 1833) | *Phylo latreillii* (Audouin & Milne Edwards, 1833)

#### Orbinia sertulata

(Savigny, 1822)

Orbinia cuvierii (Audouin & Milne Edwards, 1833)

#### Phylo foetida

(Claparède, 1869)

Aricia foetida Claparède, 1869 | *Phylo foetida* (Claparède, 1869) |*Phylo foetida foetida* (Claparède, 1870)

##### Notes

Species complex. Bleidorn et al. (2009) recover two putative cryptic species in the Atlantic and the Mediterranean (Sicily). Originally described from the Gulf of Naples, it is unknown whether more cryptic species exist in the Mediterranean and whether the Greek specimens belong to *Phylo foetida* sensu stricto or to a different species.

#### Phylo grubei

(McIntosh, 1910)

Aricia grubei McIntosh, 1919 | *Phylo grubei* (McIntosh, 1910)

#### Phylo kupfferi

(Ehlers, 1874)

Aricia kupfferi Ehlers, 1874 | *Phylo kupfferi* (Ehlers, 1874)

#### Phylo norvegica

(M. Sars in G.O. Sars, 1872)

##### Notes

Reported from Greece by Papadopoulos (1986); two additional specimens from Greece in the collections of the Senckenberg Museum (SMF 15218, 35°31'37.2"N, 27°13'28.2"E, 122 m depth, coll. date 2005-08-30, det: D. Fiege; SMF 15227, 35°31'33.6"N, 27°13'24''E, 126–142 m depth, coll. date 2005-08-30, det: D. Fiege). Records of *Phylo norvegica* from the Adriatic are doubtful and probably refer to *Phylo foetida* (Mikac 2015).

#### Protoaricia oerstedii

(Claparède, 1864)

Protoaricia oerstedii (Claparède, 1864)| *Theostoma oerstedii* (Claparède, 1864)

##### Notes

Type locality: Mediterranean (Port-Vendres, France).

#### Scoloplos armiger

(Müller, 1776)

##### Notes

Species complex. In addition to forming a complex of at least three putative cryptic species in northern Europe alone (Bleidorn et al. 2006), *Scoloplos armiger* is also easily confused with congeners (e.g. the two Mediterranean species *Scoloplos haasi* (Monro, 1937) and *Scoloplos typicus* (Eisig, 1914)).

#### Scoloplos chevalieri candiensis

Harmelin, 1969

##### Notes

Originally described from the south coast of Crete (Kaloi Limenes, 7–11 m depth; meadows of *Halophila stipulacea* and *Cymodocea nodosa*).

#### Scoloplos haasi

(Monro, 1937)

Scolaricia haasi Monro, 1937 | *Scoloplos haasi* (Monro, 1937)

##### Notes

Questionable status. Reported from Greece by Dounas (1986). Type locality: Mediterranean (Israel). As the species is easily confused with *Scoloplos armiger* (Müller, 1776) (López 2009), records of *Scoloplos armiger* from Greece may comprise specimens belonging to *Scoloplos haasi*. While it is quite possible that the species occurs in Greece, it has so far only been reported in grey literature and has not been found in over three decades, therefore its presence in Greece is here considered questionable.

#### Scoloplos typicus

(Eisig, 1914)

Scolaricia typica Eisig, 1914

##### Notes

As the species is easily confused with *Scoloplos armiger* (Müller, 1776) (Mikac 2015), many records of *Scoloplos armiger* from Greece may refer to *Scoloplos typicus*. Type locality: Mediterranean (Gulf of Naples).

### Oweniidae Rioja, 1917

#### Galathowenia fragilis

(Nilsen & Holthe, 1985)

Myriochele fragilis Nilsen & Holthe, 1985

##### Notes

Previously only known from the Arctic, the species was found in deep waters (>4000 m) off Crete by Fiege et al. (2000a) in large numbers, constituting to date the only records from Greece and the Mediterranean.

#### Galathowenia oculata

(Zachs, 1923)

Galathowenia oculata (Zachs, 1923) | *Myriochele oculata* Zachs, 1923

##### Notes

Martín (1989) and Simboura (1996) note some morphological variation (constant presence of the third mid-dorsal pygidial lobe) in Mediterranean specimens compared to Atlantic and Pacific material.

#### Myriochele heeri

Malmgren, 1867

##### Notes

Questionable status. Martín (1989) re-examined material identified as *Myriochele heeri* in the Mediterranean and assigned the material and the description of *Myriochele heeri* by Fauvel (1927) to *Galathowenia oculata* (Zachs, 1923). Parapar (2006) revised and re-described *Myriochele heeri*, as the original type series of *Myriochele heeri* constituted a mixture of different and confused species. The species is probably restricted to the boreal Atlantic and Arctic regions and Mediterranean records are questionable (Parapar 2006). Specimens identified using Fauvel's key are likely to belong to *Galathowenia oculata*.

#### Owenia fusiformis

Delle Chiaje, 1844

##### Notes

Species complex. *Owenia fusiformis* was long believed to be a cosmopolitan species but in fact contains a number of pseudo-cryptic species worldwide (e.g. Martín et al. 2006). *Owenia fusiformis* sensu stricto was originally described from Sicily and currently no evidence exists for the presence of more than one species of the complex in the Mediterranean.

### Paralacydoniidae Pettibone, 1963

#### Paralacydonia paradoxa

Fauvel, 1913

##### Notes

Type locality: Mediterranean (Monaco).

### Paraonidae Cerruti, 1909

#### Aricidea (Acmira) assimilis

Tebble, 1959

Aricidea assimilis Tebble, 1959 | *Aricidea mutabilis* Laubier & Ramos, 1974

##### Notes

*Aricidea assimilis* was included in the checklist by Simboura and Nicolaidou (2001) as separate from *Aricidea mutabilis*, but the species are considered synonymous by Katzmann and Laubier (1975) and *Aricidea mutabilis* has not been re-instated since. The species has often been misidentified as Aricidea (Acmira) lopezi Berkeley & Berkeley, 1956 (see notes there) and may in fact be a complex of species (Langeneck 2017). Type locality: Mediterranean (Israel).

#### Aricidea (Acmira) catherinae

Laubier, 1967

Aricidea catherinae Laubier, 1967 | Aricidea (Acmira) catherinae Laubier, 1967

##### Notes

Species complex. Molecular and morphological analyses by Langeneck (2017) revealed that specimens from the Mediterranean identified as Aricidea (Acmira) catherinae contain at least three pseudocryptic species which can be differentiated genetically, morphologically and on the basis of ecological characters. Type locality: Mediterranean (Banyuls-sur-Mer, France).

#### Aricidea (Acmira) cerrutii

Laubier, 1966

Aricidea (Acmira) cerrutii Laubier, 1966 | *Aricidea cerrutii* Laubier, 1966 | *Aricidea jeffreysii* (McIntosh, 1879) [sensu Cerruti / sensu Fauvel] | *Paraonis paucibranchiata* Cerruti, 1909

##### Notes

*Aricidea jeffreysii* sensu Cerruti, 1909 (non McIntosh, 1879), on which the description and illustrations of Fauvel (1927) are based, was referred to Aricidea (Acmira) cerrutii by Laubier (1967). *Paraonis paucibranciata* is considered a juvenile form by Strelzov (1973) who placed it into synonymy with Aricidea (Acmira) cerrutii. However, *Paraonis paucibranchiata* in this case would have nomenclatural priority over *Aricidea cerrutii* — an issue which is not resolved here. Type locality: Mediterranean (Albères, France).

#### Aricidea (Acmira) lopezi

Berkeley & Berkeley, 1956

Aricidea fauveli Hartman, 1957| *Aricidea lopezi lopezi* Berkeley & Berkeley, 1956

##### Notes

Questionable status. Records from Greece exist almost exclusively under the name *Aricidea fauveli*, despite the fact that they were recorded after Strelzov (1973) had synonymised the two species. It is therefore likely that they were identified using the key by Day (1967) which only includes *Aricidea fauveli*. Castelli (1987) attributes several Mediterranean records of *Aricidea lopezi* to Aricidea (Acmira) assimilis Tebble, 1959, which he considers synonymous with *Aricidea fauveli*. In the Mediterranean, *Aricidea lopezi / Aricidea fauveli* used to be distinguished from *Aricidea assimilis* on the basis of the length of the antenna (short in *Aricidea lopezi*, long in *Aricidea assimilis*) but molecular analyses by Langeneck (2017) revealed two clades related to depth which were incongruent with the two different morphotypes. It is therefore likely that Mediterranean specimens of Aricidea (Acmira) lopezi belong to different species.

#### Aricidea (Acmira) neosuecica

Hartman, 1965 sensu Laubier & Ramos, 1974

Aricidea neosuecica Hartman, 1965 | *Aricidea neosuecica* Hartman, 1965 sensu Laubier & Ramos, 1974

##### Notes

Questionable status. Amongst the Greek reports of the species, only Arvanitidis (1994) explicitly refers his specimens to *Aricidea neosuecica* sensu Laubier and Ramos 1974 (non Hartman, 1965). Laubier and Ramos' description of a single specimen of Aricidea cf. neosuecica differs substantially from that of Hartman (1965b) from New England (Arvanitidis 1994) and could constitute a juvenile of Aricidea (Acmira) simonae Laubier & Ramos 1974 (Hartley 1981), whereas Hartman's species is referred to Aricidea (Acmira) simplex Day, 1963 by Aguirrezabalaga (2012). The actual identity of the Greek specimens is currently unknown.

#### Aricidea (Acmira) simonae

Laubier & Ramos, 1974

Aricidea simonae Laubier & Ramos, 1974 | Aricidea (Acmira) simonae Laubier & Ramos, 1974

##### Notes

Type locality: Mediterranean (Adriatic).

#### Aricidea (Acmira) simplex

Day, 1963

Aricidea (Acesta) simplex Day, 1963 | Aricidea (Acmira) simplex Day, 1963 | *Aricidea simplex* Day, 1963| *Aricidea suecica simplex* Day, 1963

##### Notes

The species is poorly defined and, as a result, has been reported from a variety of habitats and locations worldwide; Mediterranean specimens probably belong to *Aricidea simonae* (Langeneck 2017).

#### Aricidea (Aricidea) capensis

Day, 1961

Aricidea (Aricidea) capensis Day, 1961 | *Aricidea capensis* Day, 1961

##### Notes

Questionable status. Aricidea (Aricidea) capensis is usually reported from the Mediterranean as the subspecies Aricidea (Aricidea) capensis bansei Laubier & Ramos, 1974, whereas *Aricidea capensis* is considered to be restricted to South Africa. Arvanitidis (1994) refers the Greek records of *Aricidea capensis* by Bogdanos and Satsmadjis (1983) and Karakassis (1991) to Aricidea (Aricidea) capensis bansei. However, *Aricidea capensis* is frequently reported from Greece at species level (Simboura and Nicolaidou 2001) and Conides et al. (1999) report both *Aricidea capensis* and *Aricidea capensis bansei* in the same study.

#### Aricidea (Aricidea) capensis bansei

Laubier & Ramos, 1974

Aricidea (Aricidea) capensis bansei Day, 1961 | *Aricidea capensis bansei* Laubier & Ramos, 1974

##### Notes

Type locality: Mediterranean (Baie de Rosas, Spain).

#### Aricidea (Aricidea) longicirrata

Hartmann-Schröder, 1965

Aricidea longicirrata Hartmann-Schröder, 1965

##### Notes

Questionable status. Reported from Greece by Xenopoulou (1987), no other Mediterranean records. Distributed along the Pacific coast of tropical and South America (de León-González et al. 2006).

#### Aricidea (Aricidea) longobranchiata

Day, 1961

Aricidea (Aricidea) longobranchiata Day, 1961 | *Aricidea longobranchiata* Day, 1961

##### Notes

Questionable status. In the Mediterranean reported from Greece (e.g. Bogdanos and Satsmadjis 1983, Bogdanos and Satsmadjis 1987, Simboura 1996; full reference list in Suppl. material 2) and France (Grémare et al. 1998) Records from Turkey belong to Aricidea (Acmira) assimilis Tebble, 1959 (Çınar et al. 2014). Otherwise distributed in tropical waters of the Atlantic and Indian Ocean and on the coasts of South Africa.

#### Aricidea (Aricidea) minima

Strelzov, 1973

Aricidea minima Strelzov, 1973

##### Notes

Questionable status. In the Mediterranean only reported from Greece (Spiliotis 1991, Zenetos et al. 1997). Originally described from the Patagonian Shelf.

#### Aricidea (Aricidea) minuta

Southward, 1956

Aricidea (Aricidea) minuta Southward, 1956 | *Aricidea minuta* Southward, 1956

##### Notes

Reported from Greece by Simboura (1996), in the Mediterranean also reported from the coasts of Spain (Laubier and Ramos 1974). Otherwise distributed along the Atlantic coasts of Europe.

#### Aricidea (Aricidea) pseudoarticulata

Hobson, 1972

Aricidea (Aricidea) fragilis mediterranea Laubier & Ramos, 1974 | *Aricidea fragilis mediterranea* Laubier & Ramos, 1974 | *Aricidea pseudoarticulata* Hobson, 1972

##### Notes

*Aricidea fragilis mediterranea* – a Mediterranean species originally described from southern France – was synonymised with *Aricidea pseudoarticulata* by Aguirrezabalaga and Gil (2008) but without examination of type material. This synonymy extends the distribution range of *Aricidea pseudoarticulata* to nearly cosmopolitan, including the Pacific (native range of *Aricidea pseudoarticulata*) and Atlantic Oceans and the Mediterranean Sea.

#### Aricidea (Aricidea) rosea

Reish, 1968

Aricidea (Acmira) lopezi rosea Reish, 1968 | *Aricidea lopezi rosea* Reish, 1968

##### Notes

Questionable status. In the Mediterranean only reported from Greece (NCMR 1989, Simboura 1996; based on the same specimens). Distributed along the Pacific coasts of America.

#### Aricidea (Aricidea) wassi

Pettibone, 1965

Aricidea wassi Pettibone, 1965 | Aricidea (Aricidea) wassi Pettibone, 1965

##### Notes

Reported from Greece by Simboura and Zenetos (2005). In the Mediterranean also reported from Italy (Castelli et al. 2008), Egypt (Abd-Elnaby 2009), Turkey (Çınar et al. 2014) and the Adriatic (Mikac 2015), otherwise reported from the North Atlantic and North Pacific (Hartley 1981).

#### Aricidea (Strelzovia) belgicae

(Fauvel, 1936)

Aedicira belgicae (Fauvel, 1936) | *Paraonis belgicae* Fauvel, 1936

##### Notes

Questionable status. The species has a complex taxonomic history. Most reports and taxonomic accounts are not based on the holotype, but on a re-description by Monro (1939), which was later found to be based on non-type specimens belonging to two different species (Hartley 1984, López 2008). Aricidea (Strelzovia) belgicae had been considered eurybathic and cosmopolitan, but is in fact considered restricted to the deep waters of the Antarctic and most reports (including those from Greece, see Suppl. material 2 for a full list) must be considered misidentifications (López 2008).

#### Aricidea (Strelzovia) claudiae

Laubier, 1967

Aricidea (Allia) claudiae Laubier, 1967 | *Aricidea claudiae* Laubier, 1967

##### Notes

Type locality: Mediterranean (Banyuls-sur-Mer, France).

#### Aricidea (Strelzovia) curviseta

Day, 1963

Aricidea (Allia) curviseta Day, 1963 | *Aricidea curviseta* Day, 1963

##### Notes

Questionable status. In the Mediterranean only reported from Greece (e.g. Bogdanos and Satsmadjis 1983, Bogdanos and Satsmadjis 1987, Simboura 1996; full reference list in Suppl. material 2). Distributed in the Indian Ocean and tropical Atlantic. As the species shows a wide polymorphism of the prostomial antenna (Strelzov 1973), Mediterranean specimens could have been misidentified and belong in fact to Aricidea (Strelzovia) claudiae Laubier, 1967 (J. Langeneck, pers. comm).

#### Aricidea (Strelzovia) hartmani

Strelzov, 1968

Aricidea (Allia) hartmani Strelzov, 1968 | *Aricidea hartmani* Strelzov, 1968

##### Notes

Questionable status. In the Mediterranean only reported from Greece (NCMR 1992, Simboura 1996, Zenetos et al. 1997, Pancucci-Papadopoulou et al. 1999); these could in fact belong to the Mediterranean species Aricidea (Strelzovia) mariannae Katzmann & Laubier, 1975, which shows similarities to Aricidea (Strelzovia) hartmani, especially in shape and length of the antenna (J. Langeneck, pers. comm). Aricidea (Strelzovia) hartmani is commonly distributed in Arctic regions.

#### Aricidea (Strelzovia) mediterranea

(Laubier & Ramos, 1974)

Aedicira mediterranea Laubier & Ramos, 1974

##### Notes

The species was described as including two different morphological forms (based on antennal shape) by Laubier and Ramos (1974) from the Western Mediterranean. Aguirrezabalaga and Gil (2009) revised the species and split Aricidea (Strelzovia) mediterranea into two species based on these forms, Aricidea (Strelzovia) mediterranea and Aricidea (Strelzovia) sardai Aguirrezabalaga & Gil, 2009. The records from Greece could belong to either of the two species and require further examination.

#### Aricidea (Strelzovia) monicae

Laubier, 1967

Aricidea (Allia) monicae Laubier, 1967 | *Aricidea monicae* Laubier, 1967

##### Notes

Reported from Greece by Koulouri et al. (2015). Type locality: Mediterranean (Banyuls-sur-Mer, France).

#### Aricidea (Strelzovia) quadrilobata

Webster & Benedict, 1887

Aricidea quadrilobata Webster & Benedict, 1887

##### Notes

Questionable status. Reported from Greece by Dounas (1986). In the Mediterranean also reported from Italy (Castelli et al. 2008), the Adriatic (Mikac 2015) and the Malta Escarpment (Langeneck et al. 2017b), otherwise known from the North Atlantic. Aricidea (Strelzovia) quadrilobata sensu Strelzov (1973) was referred to Aricidea (Strelzovia) antennata Annenkova, 1934 by Aguirrezabalaga and Gil (2009). Thus, if Strelzov's work was used to identify the Greek specimens of Aricidea (Strelzovia) quadrilobata, they may actually correspond to *Aricidea antennata*. In addition, as the species has so far only been reported in grey literature and has not been found in over three decades, its presence in Greece is here considered questionable.

#### Aricidea (Strelzovia) suecica

Eliason, 1920

Aricidea suecica Eliason, 1920

##### Notes

Questionable status. Usually reported in the Mediterranean as the subspecies *Aricidea suecica meridionalis* Laubier & Ramos, 1974, but Dando et al. (1995) and Koulouri et al. (2015) report *Aricidea suecica* at species level.

#### Aricidea (Strelzov﻿ia) suecica meridionalis

Laubier & Ramos, 1974

Aricidea suecica meridionalis Laubier & Ramos, 1974

##### Notes

Type locality: Mediterranean (Baie de Rosas, Spain).

#### Cirrophorus branchiatus

Ehlers, 1908

Cirrophorus branchiatus Ehlers, 1908| *Cirrophorus lyriformis* (Annenkova, 1934)

#### Cirrophorus furcatus

(Hartman, 1957)

##### Notes

Questionable status. Frequently reported from the Mediterranean and from Greece, but Mediterranean specimens probably belong to different species (Castelli et al. 2008), such as the recently described *Cirrophorus nikebianchii* Langeneck, Barbieri, Maltagliati & Castelli, 2017 or *Cirrophorus turcicus* Erdoğan-Dereli, Çınar & Dağli, 2017.

#### Cirrophorus nikebianchii

Langeneck, Barbieri, Maltagliati & Castelli, 2017

##### Notes

Reported from Greece by Langeneck et al. (2017a). Type locality: Mediterranean (Sardinia).

#### Levinsenia demiri

Çınar, Dağlı & Açik, 2011

##### Notes

Reported from Greece by Katsiaras and Simboura (2015). Type locality: Sea of Marmara.

#### Levinsenia gracilis

(Tauber, 1879)

Levinsenia gracilis (Tauber, 1879) | *Paraonis gracilis* (Tauber, 1879) | *Paraonis gracilis gracilis* (Tauber, 1879) | *Tauberia gracilis* (Tauber, 1879)

#### Levinsenia multibranchiata

(Hartman, 1957)

Levinsenia multibranchiata (Hartman, 1957) | *Tauberia multibranchiata* (Hartman, 1957)

##### Notes

Questionable status. In the Mediterranean only reported from Greece (Nicolaidou et al. 1993, IOFR 1984, Papadopoulos 1986). Originally described from southern California.

#### Levinsenia oculata

(Hartman, 1957)

Levinsenia oculata (Hartman, 1957) |*Paraonis gracilis oculata* Hartman, 1957

##### Notes

Questionable status. In the Mediterranean reported from Greece (e.g. Bogdanos and Satsmadjis 1983, Bogdanos and Satsmadjis 1987, Simboura 1996; full reference list in Suppl. material 2) and Italy (Castelli et al. 2008). Italian specimens probably belong to an undescribed species (Castelli et al. 2008). Originally described from southern California.

#### Levinsenia reducta

(Hartman, 1965)

Levinsenia reducta (Hartman, 1965) | *Tauberia reducta* (Hartman, 1965)

##### Notes

Questionable status. In the Mediterranean reported from Greece (e.g. Simboura 1987, Simboura 1996, Makra and Nicolaidou 2000, Bogdanos et al. 2002; full reference list in Suppl. material 2) and Egypt (Abd-Elnaby 2009). Commonly distributed in the Caribbean and the tropical West Atlantic.

#### Levinsenia tribranchiata

Çınar, Dağlı & Açik, 2011

##### Notes

Reported from Greece by Katsiaras and Simboura (2015). Type locality: Sea of Marmara.

#### Paradoneis armata

Glémarec, 1966

Cirrophorus harpagoneus (Storch, 1967) | *Paradoneis armata* Glémarec, 1966

##### Notes

López et al. (1987) compared type material of *Paradoneis armata* to the description of *Cirrophorus harpagoneus* (the type material of which is lost). The presumably differentiating character (acicular chaetae with subdistal teeth) is present in both species, thus López et al. (1987) consider the two species synonymous. However, as the species were described from different biogeographic regions (*Paradoneis armata*: Atlantic, *Paradoneis harpagoneus*: Red Sea) and the type material of *Paradoneis harpagoneus* is lost, not all authors share this view and examination of material from the type locality would be needed to clarify the status of the taxa (Langeneck 2017).

#### Paradoneis drachi

Laubier & Ramos, 1974

##### Notes

Reported from Greece by Simboura (1996) based on a single specimen, identification confirmed by A. Castelli (pers. comm. in Simboura 1996). Type locality: Mediterranean (Baie de Rosas, Spain).

#### Paradoneis ilvana

Castelli, 1985

##### Notes

Reported from Greece by Arvanitidis (2000a). Type locality: Mediterranean (coast of Tuscany).

#### Paradoneis lyra

(Southern, 1914)

Cirrophorus lyra (Southern, 1914) | *Paradoneis lyra* (Southern, 1914) | *Paraonis lyra* Southern, 1914

#### Paraonis fulgens

(Levinsen, 1884)

##### Notes

Reported from Greece by Xenopoulou (1987), also found by Papageorgiou et al. (2006) (unpublished data). In the Mediterranean also reported from the Gulf of Lions (Laubier and Ramos 1974), Spain (Torres-Gavila 2008), Italy (Castelli et al. 2008) and the Adriatic (Mikac 2015), otherwise distributed along the Atlantic coasts of Europe and North America.

### Pectinariidae Quatrefages, 1866

#### Amphictene auricoma

(O.F. Müller, 1776)

Amphictene auricoma (O.F. Müller, 1776) | Pectinaria (Amphictene) auricoma (O.F. Müller, 1776) | *Pectinaria auricoma* (O.F. Müller, 1776)

#### Amphictene capensis

(Pallas, 1766)

Pectinaria capensis (Pallas, 1766)

##### Notes

Questionable status. In the Mediterranean only reported from Greece (e.g. Bogdanos and Satsmadjis 1983, Simboura et al. 1995a, Simboura 1996; full reference list in Suppl. material 2). Commonly distributed along the coasts of South Africa and in the Indo-Pacific.

#### Lagis koreni

Malmgren, 1866

Lagis koreni Malmgren, 1866 | Pectinaria (Lagis) koreni (Malmgren, 1866) | *Pectinaria koreni* (Malmgren, 1866)

##### Notes

Species complex. At least two cryptic species with a strong genetic divergence exist along the north coast of France (Jolly et al. 2005, Jolly et al. 2006).

#### Lagis neapolitana

Claparède, 1869

Pectinaria (Lagis) neapolitana (Claparède, 1869) | *Pectinaria neapolitana* Claparède, 1869

##### Notes

Reported from Greece by Arvanitidis (2000a). Type locality: Mediterranean (Gulf of Naples). Considered a synonym of *Lagis koreni* Malmgren, 1866 by Fauvel (1927), thus it may have been overlooked in the area. Surugiu (2006) provides an overview of the differences between *Lagis koreni* and *Lagis neapolitana*.

#### Pectinaria belgica

(Pallas, 1766)

Pectinaria (Pectinaria) belgica (Pallas, 1766)

#### Petta pusilla

Malmgren, 1866

##### Notes

Reported from worldwide locations, but records from outside the North-East Atlantic probably belong to other species (Hutchings and Peart 2002).

### Phyllodocidae Ørsted, 1843

#### Eteone flava

(Fabricius, 1780)

##### Notes

Reported from Greece by Arvanitidis (2000a) based on a single specimen, in the Mediterranean also known from the western basin (Fredj 1974) and the Adriatic (Mikac 2015); otherwise distributed in the boreal Atlantic.

#### Eteone longa

(Fabricius, 1780)

##### Notes

Species complex. Consists of at least four putative cryptic species in the Arctic Ocean (Carr et al. 2011). It is unknown whether the Greek specimens belong to any of these or to a different species.

#### Eulalia bilineata

(Johnston, 1840)

##### Notes

Questionable status. Reported from Greece by Georgiou (1983) from Mazoma lagoon, Amvrakikos Gulf. In the Mediterranean also reported from the western basin (Fauvel 1923, Bellan 1964a) and the Adriatic (Mikac 2015); otherwise distributed in the North Atlantic. Taking into account its North Atlantic distribution, the rarity of Mediterranean records and the fact that, despite regular monitoring of the specific lagoonal system, the species has never been found again, the record must be considered questionable.

#### Eulalia clavigera

(Audouin & Milne Edwards, 1833)

##### Notes

Reported from Greece by Keklikoglou et al. (2013) and Maidanou et al. (2017). In the Mediterranean also known from Cyprus (Çınar 2005), Tunisia (Zaâbi 2013), Turkey (Çınar et al. 2014) and Egypt (Dorgham et al. 2014), otherwise distributed along the Atlantic coasts of Europe. Possibly underreported, as Bonse et al. (1996) refer the description and illustrations of *Eulalia viridis* (Linnaeus, 1767) by Fauvel (1923) to *Eulalia clavigera*, thus individuals of *Eulalia viridis* identified using Fauvel's publication probably belong to *Eulalia clavigera*.

#### Eulalia tripunctata

McIntosh, 1874

##### Notes

Reported from Greece by Arvanitidis (2000a). In the Mediterranean also reported from France (e.g. Bellan 1964a), Spain (Campoy 1982), Israel (Fishelson 2000), Cyprus (Çınar 2005), Italy (Castelli et al. 2008), Turkey (Çınar et al. 2014) and the Adriatic (Mikac 2015), otherwise distributed along the Atlantic coasts of Europe.

#### Eulalia viridis

(Linnaeus, 1767)

##### Notes

Species complex. *Eulalia viridis* contains at least two different species in northern Europe (Bonse et al. 1996). Greek specimens could belong to either of these or to a different species; however, identifications made using Fauvel (1923) probably belong to *Eulalia clavigera* (Audouin & Milne Edwards, 1833) (see remarks there).

#### Eumida punctifera

(Grube, 1860)

Eulalia punctifera (Grube, 1860) | *Eumida punctifera* (Grube, 1860) | *Pirakia punctifera* (Grube, 1860)

#### Eumida sanguinea

(Ørsted, 1843)

Eulalia sanguinea Ørsted, 1843 | *Eumida sanguinea* (Ørsted, 1843)

##### Notes

Species complex. *Nygren and Pleijel (2011)* recovered ten cryptic species in European specimens identified as *Eumida sanguinea*. They restrict the distribution of *Eumida sanguinea* to the Skagerrak, Kattegat and southern England. Five cryptic species were discovered from the Mediterranean: *Eumida asterope* Nygren & Pleijel 2011, *Eumida maia* Nygren & Pleijel 2011, *Eumida merobe* Nygren & Pleijel 2011, *Eumida* sp. (all from southern France and morphologically identical to *Eumida sanguinea* except for their pigmentation patterns) and *Eumida taygete* Nygren & Pleijel 2011 from Croatia, (morphologically identical to *Eumida sanguinea* sensu stricto). These new species are almost impossible to identify based on their morphology, thus the authors recommend the use of the name "*Eumida sanguinea* sensu lato" or "*Eumida sanguinea* complex" for all records which are not resolved through molecular methods.

#### Hesionura coineaui

(Laubier, 1962)

##### Notes

New record for Greece. Three specimens, Elafonisi, Crete, 35°16'20.7"N, 23°32'15.9"E, 1 m depth, fine sand; 3 specimens, Pachia Ammos, Crete, 35°06'39.6"N, 25°48'32.4"E, 1–5 m depth, fine to coarse sand, collected in the framework of the MEDCORE project (unpublished data from Papageorgiou et al. 2006). Literature used for identification: Alós (2004). Type locality: Mediterranean (Argèles, France).

#### Hesionura elongata

(Southern, 1914)

Hesionura elongata (Southern, 1914) | *Pseudomystides elongata* (Southern, 1914)

#### Hypereteone foliosa

(Quatrefages, 1865)

Eteone lactea Claparède, 1868

##### Notes

Reported from Greece by Simboura (1996) and NCMR (1997) as *Eteone lactea*, a Mediterranean species (type locality Gulf of Naples), which was synonymised with *Hypereteone foliosa* by Pleijel (1991).

#### Krohnia lepidota

(Krohn, 1845)

Callizonella lepidota (Krohn, 1845)

##### Notes

Reported from Greece by Wesenberg-Lund (1939a). Type locality: Mediterranean (Sicily).

#### Mysta picta

(Quatrefages, 1866)

Eteone picta Quatrefages, 1866 | *Mysta picta* (Quatrefages, 1866)

#### Mysta siphodonta

(Delle Chiaje, 1822)

Eteone siphonodonta Claparède, 1868 | *Eteone syphodonta* (Delle Chiaje, 1822) | Eteone (Mysta) siphonodonta (Claparède, 1868) | *Mysta siphonodonta* (Claparède, 1868)

##### Notes

Type locality: Mediterranean (Gulf of Naples).

#### Mystides borealis

Théel, 1879

##### Notes

Questionable status. Frequently reported from the Mediterranean (e.g. Castelli et al. 2008, Mikac 2015), but Pleijel and Dales (1991) and Pleijel (1993) consider the species to be restricted to the Arctic and the Mediterranean specimens to belong to *Mystides caeca*. Specimens from Cyprus were re-identified as *Mystides caeca* Langerhans, 1880 by Çınar (2005).

#### Mystides caeca

Langerhans, 1880

##### Notes

Arvanitidis (2000a) lists Aegean records of *Mystides borealis* Théel, 1879 under the name *Mystides caeca*, following Pleijel and Dales (1991) and Pleijel (1993) who consider Mediterranean specimens to belong to *Mystides caeca*. No published reports of *Mystides caeca* exist from Greece but the presence of the species has been confirmed recently (K. Keklikoglou, unpublished data).

#### Naiades cantrainii

Delle Chiaje, 1828

Alciopa cantraini (Delle Chiaje, 1828)

##### Notes

Reported from Greece by Wesenberg-Lund (1939a) and Gogou (2009). Type locality: Mediterranean (Gulf of Naples).

#### Nereiphylla paretti

Blainville, 1828

Nereiphylla paretti Blainville, 1828 | *Phyllodoce paretti* (Blainville, 1828)

##### Notes

Reported from Greece by Vamvakas (1971) and NCMR (2000b). In the Mediterranean also known from France (Bellan 1964a), Spain (Méndez and Cardell 1996), Cyprus (Çınar 2005), Italy (Castelli et al. 2008), Turkey (Çınar et al. 2014) and the Adriatic (Mikac 2015); otherwise distributed along the Atlantic coasts of Europe and in the Black Sea.

#### Nereiphylla pusilla

(Claparède, 1870)

Genetyllis nana (de Saint Joseph, 1908) | *Nereiphylla pusilla* (Claparède, 1870) | *Paranaitis pusilla* (Claparède, 1870)

##### Notes

Type locality: Mediterranean (Gulf of Naples).

#### Nereiphylla rubiginosa

(Saint-Joseph, 1888)

Eulalia rubiginosa (de Saint Joseph, 1888) | *Genetyllis rubiginosa* (de Saint-Joseph, 1888) | *Nereiphylla rubiginosa* (de Saint-Joseph, 1888) | *Phyllodoce rubiginosa* de Saint-Joseph, 1888

#### Notophyllum foliosum

(Sars, 1835)

##### Notes

Species complex. At least two cryptic species exist in Scandinavia, differentiated by depth preference and colour pattern (Nygren et al. 2010a). The deeper form was described as *Notophyllum crypticum* Nygren, Eklöf & Pleijel, 2010. The authors do not exclude further cryptic species in the complex.

#### Paranaitis kosteriensis

(Malmgren, 1867)

##### Notes

Reported from Greece by Eleftheriou et al. (1990) and Karakassis (1991). In the Mediterranean also known from France (Guille and Laubier 1966), Italy (Castelli et al. 2008) and Spain (Torres-Gavila 2008), otherwise distributed along the Atlantic coasts of Europe. Confirmed from Sicily by Kato and Pleijel (2003).

#### Phyllodoce groenlandica

Ørsted, 1842

##### Notes

Questionable status. Species complex. *Phyllodoce groenlandica* has been shown to comprise at least two different cryptic species in Canadian waters (Carr et al. 2011). Pleijel (1988) considers the species to be restricted to circumboreal areas.

#### Phyllodoce laminosa

Savigny in Lamarck, 1818

##### Notes

Type locality: Mediterranean (Nice, France).

#### Phyllodoce lineata

(Claparède, 1870)

Anaitides lineata (Claparède, 1870) | *Paranaitis lineata* (Claparède, 1870) | Phyllodoce (Anaitides) lineata (Claparède, 1870) | Phyllodoce (Paranaitis) lineata (Claparède, 1870) | *Phyllodoce lineata* (Claparède, 1870)

##### Notes

Type locality: Mediterranean (Gulf of Naples).

#### Phyllodoce longipes

Kinberg, 1866

##### Notes

Reported from Greece by Maidanou et al. (2017). In the Mediterranean also known from the Adriatic (Mikac 2015, records confirmed by F. Pleijel and A. Nygren). Otherwise widely distributed (Chile, Gulf of Mexico, North Atlantic) and, although specimens from these location do not differ morphologically, they probably belong to different species (Nygren and Pleijel 2015).

#### Phyllodoce maculata

(Linnaeus, 1767)

Anaitides maculata (Linnaeus, 1767) | *Phyllodoce maculata* (Linnaeus, 1767)

##### Notes

Easily confused with *Phyllodoce mucosa* Ørsted, 1843; probably restricted to the Atlantic coasts of Europe, the Arctic and the Sea of Japan (Pleijel 1988).

#### Phyllodoce madeirensis

Langerhans, 1880

Anaitides madeirensis (Langerhans, 1880) | *Phyllodoce madeirensis* Langerhans, 1880

##### Notes

Species complex. Recent molecular analyses by Ravara et al. (2017) recover three distinct putative cryptic species from specimens identified as *Phyllodoce madeirensis* in the North-East Atlantic.

#### Phyllodoce mucosa

Ørsted, 1843

Anaitides mucosa (Ørsted, 1843) | Phyllodoce (Anaitides) mucosa Ørsted, 1843 |*Phyllodoce mucosa* Ørsted, 1843

##### Notes

Easily confused with *Phyllodoce maculata* (Linnaeus, 1767); probably restricted to the Atlantic coasts of Europe to the White Sea (Pleijel 1988).

#### Phyllodoce rosea

(McIntosh, 1877)

##### Notes

Reported from Greece by Chatzigeorgiou et al. (2016) based on a single specimen. In the Mediterranean also reported from Spain (San Martín et al. 1981a), Italy (Castelli et al. 2008), Turkey (Çınar et al. 2014) and the Adriatic (Mikac 2015). Otherwise distributed along the Atlantic coasts of Europe (Pleijel 1988).

#### Pseudomystides limbata

(Saint-Joseph, 1888)

Mystides limbata Saint-Joseph, 1888 | *Pseudomystides limbata* (Saint-Joseph, 1888)

#### Pseudomystides limbata nigrolineata

(Rioja, 1925)

##### Notes

Reported from Greece by Papadopoulos (1986) and Nicolaidou et al. (1993). In the Mediterranean also known from Spain (López García 1995); otherwise known from the Atlantic coast of the Iberian Peninsula. Reports of *Pseudomystides limbata* (Saint-Joseph, 1888) could comprise specimens belonging to *Pseudomystides limbata nigrolineata*.

#### Pterocirrus macroceros

(Grube, 1860)

Eulalia macroceros (Grube, 1860) | Eulalia (Pterocirrus) macroceros (Grube, 1860) | *Pterocirrus macroceros* (Grube, 1860) |

##### Notes

Type locality: Mediterranean (Adriatic).

#### Rhynchonereella moebii

(Apstein, 1893)

Callizona moebii Apstein, 1893

##### Notes

Reported from Greece by Wesenberg-Lund (1939a). Type locality: Mediterranean (Sicily).

#### Torrea candida

(Delle Chiaje, 1841)

##### Notes

Reported from Greece by Wesenberg-Lund (1939a). Type locality: Mediterranean (Sicily).

#### Vanadis crystallina

Greeff, 1876

##### Notes

Questionable status. Reported from Greece by Wesenberg-Lund (1939a). Type locality: Mediterranean (Gulf of Naples). However, Wesenberg-Lund's depiction of *Vanadis crystallina* actually shows *Vanadis minuta* Treadwell, 1906 (Dales 1957). Without re-examination of Wesenberg-Lund's specimens, it remains unclear which of them belong to *Vanadis crystallina* and which to *Vanadis minuta*.

#### Vanadis formosa

Claparède, 1870

##### Notes

Reported from Greece by Wesenberg-Lund (1939a). Type locality: Mediterranean (Gulf of Naples).

### Pilargidae de Saint-Joseph, 1899

#### Ancistrosyllis falcata

(Day, 1957)

Ancistrosyllis falcata (Day, 1957) | *Pilargis falcata* Day, 1957

##### Notes

Questionable status. In the Mediterranean only reported from Greece (e.g. Bogdanos and Satsmadjis 1983, Bogdanos and Nicolaidou 1985, Bogdanos and Satsmadjis 1987, Nicolaidou and Papadopoulou 1989; full reference list in Suppl. material 2). Commonly distributed in South Africa and the Indian Ocean.

#### Ancistrosyllis fioronii

Fiege & Böggemann, 1999

##### Notes

One specimen from Greece in the collections of the Senckenberg Museum (SMF 11218, 39°16'05.4"N, 23°43'04.8"E, 1252–1255 m depth, coll. date 1998-01-01, det. D. Fiege). No other Mediterranean records. Originally described from the North Sea.

#### Ancistrosyllis groenlandica

McIntosh, 1879

#### Ancistrosyllis hamata

(Hartman, 1960)

Ancistargis hamata (Hartman, 1960) | *Ancistrosyllis hamata* (Hartman, 1960)

##### Notes

Reported from Greece by Arvanitidis (1994) and Simboura (1996). In the Mediterranean also known from Spain (Campoy 1982), Israel (Ben-Eliahu 1995), Italy (Castelli et al. 2008) and Turkey (Çınar et al. 2014), otherwise known from the temperate and tropical Atlantic and from the coasts of California.

#### Otopsis chardyi

Katzmann, 1974

##### Notes

Reported from Greece by Arvanitidis (2000a) based on a single specimen. Type locality: Mediterranean (Adriatic).

#### Pilargis verrucosa

Saint-Joseph, 1899

#### Pseudexogone dineti

(Katzmann, Laubier & Ramos, 1974)

Synelmis dineti Katzmann, Laubier & Ramos, 1974

##### Notes

Reported from Greece by Simboura (1996). Type locality: Mediterranean (Adriatic).

#### Sigambra parva

(Day, 1963)

Ancistrosyllis parva Day, 1963 | *Sigambra parva* (Day, 1963)

##### Ecological interactions

###### Native status

Non-native (established)

##### Notes

Considered a synonym of *Sigambra tentaculata* (Treadwell, 1941) until it was re-established by Moreira and Parapar (2002) based on differences in the arrangement and number of the pharyngeal papillae. Still, a level of confusion concerning the species in the *Sigambra tentaculata* complex and their distribution remains. Moreira and Parapar (2002) consider *Sigambra tentaculata* to possibly be restricted to the East coast of North America and identify specimens from the Mediterranean and Atlantic coasts of Spain as *Sigambra parva* – a non-native species originally described from South Africa. On the other hand, specimens of *Sigambra parva* from the Turkish Aegean were re-identified as *Sigambra tentaculata* (Çınar et al. 2014). The taxonomic and native status of the genus in the Mediterranean is therefore in need of revision.

#### Sigambra robusta

(Ehlers, 1908)

Ancistrosyllis robusta Ehlers, 1908

##### Notes

Questionable status. In the Mediterranean only reported from Greece (Bogdanos and Satsmadjis 1983, Bogdanos and Nicolaidou 1985, Bogdanos and Satsmadjis 1987, Bogdanos et al. 2002). Commonly distributed in the tropical Atlantic.

#### Sigambra tentaculata

(Treadwell, 1941)

##### Notes

See notes under *Sigambra parva*.

### Polycirridae Malmgren, 1867

#### Amaeana trilobata

(Sars, 1863)

Amaeana trilobata *Amaea trilobata (Sars, 1863) | Amaeana trilobata* (Sars, 1863)

#### Polycirrus aurantiacus

Grube, 1860

##### Notes

Type locality: Mediterranean (Cres, Croatia).

#### Polycirrus denticulatus

Saint-Joseph, 1894

##### Notes

Reported from Greece by Dando et al. (1995). In the Mediterranean also known from Italy (Castelli et al. 2008) and the Adriatic (Mikac 2015). Otherwise distributed along the Atlantic coasts of Europe.

#### Polycirrus latidens

Eliason, 1962

##### Notes

Questionable status. In the Mediterranean only reported from Greece (Maidanou et al. 2017; identified using key and descriptions by Holthe (1986)). Distributed in the boreal regions of Europe.

#### Polycirrus medusa

Grube, 1850

##### Notes

Type locality: Mediterranean (neotype from Anse du Rouet, France). Specimens identified using the key and descriptions by Holthe (1986) probably belong to other species (Glasby and Hutchings 2014).

#### Polycirrus plumosus

Wollebæk, 1912

##### Notes

Questionable status. In the Mediterranean reported from Greece (Simboura 1987, Simboura 1996, Pancucci-Papadopoulou et al. 1999, Chatzigeorgiou et al. 2016) and the Adriatic (Mikac 2015), otherwise distributed in the North Atlantic. Several Greek specimens were re-examined and found to belong to *Polycirrus twisti* Potts, 1928 (Simboura et al. 2010), a Lessepsian migrant. It is likely that *Polycirrus plumosus* does not occur in Greece.

#### Polycirrus twisti

Potts, 1928

##### Ecological interactions

###### Native status

Non-native (established)

##### Notes

Originally described from the Suez Canal. Reported from Greece by Simboura (2011), found several times afterwards (HCMR, unpublished data). May have been misidentified in the past as *Polycirrus plumosus* Wollebæk, 1912 (see notes there).

### Poecilochaetidae Hannerz, 1956

#### Poecilochaetus fauchaldi

Pilato & Cantone, 1976

##### Notes

Reported from Greece by Arvanitidis (2000a). Type locality: Mediterranean (Sicily).

#### Poecilochaetus fulgoris

Claparède in Ehlers, 1875

#### Poecilochaetus serpens

Allen, 1904

### Polynoidae Kinberg, 1856

#### Acholoe squamosa

(Delle Chiaje, 1825)

Acholoe astericola (Delle Chiaje, 1841) | *Acholoe squamosa* (Delle Chiaje, 1825)

##### Notes

Opinions on the validity of the name *Acholoe squamosa* are divided. Delle Chiaje (1825) described *Nereis squamosa*, but when he moved the species to *Polynoe* he used the replacement name *Polynoe astericola* (Delle Chiaje 1841: 106) possibly to avoid confusion with Savigny's 1818 *Polynoe squamata* (Pettibone 1996). Thus, Pettibone (1996) and Barnich and Fiege (2003) use *Acholoe astericola* as the valid name. However, as Savigny's species is *Polynoe squamata* (Savigny 1818: 309), not *squamosa* and Delle Chiaje was apparently aware of this, as he uses the name *squamata* alongside *squamosa* (Delle Chiaje 1841: 57, 106). Thus, there was no secondary homonymy and, as *Nereis squamosa* is an available name, there was and is no need for a replacement name. Therefore, we here consider *Acholoe squamosa* to be the valid name (see also WoRMS for additional information). Reported from Greece by Arvanitidis (1994). Type locality: Mediterranean (Gulf of Naples).

#### Adyte hyalina

(G.O. Sars, 1873)

Adyte assimilis (McIntosh, 1874)

##### Notes

Reported from Greece by Koulouri et al. (2015). In the Mediterranean also known from Italy (Bock et al. 2010), Spain (Núñez et al. 2011) and Turkey (Çınar et al. 2014), otherwise distributed in the North East Atlantic and the North Sea (Bock et al. 2010).

#### Antinoe aequiseta

Kinberg, 1856

Harmothoe aequiseta (Kinberg, 1856)

##### Notes

Questionable status. In the Mediterranean only reported from Greece (Papadopoulos 1986). Distributed along the coasts of South Africa and in the Indian Ocean.

#### Bathyfauvelia affinis

(Fauvel, 1914)

Bathyfauvelia affinis (Fauvel, 1914) | *Macellicephala annae* Reyss, 1968

##### Notes

Reported as *Macellicephala annae* by Chardy et al. (1973) from deep waters (2000–3000 m) in the Aegean and Ionian Sea, without giving a precise location. It is highly likely that the species was found in the Aegean in Greek waters, as such depths do not occur near the Turkish coast. Pettibone (1976) synonymised the two species after having examined the holotype of *Macellicephala annae* and Arvanitidis (2000a), accordingly, uses the name *Bathyfauvelia affinis* in his checklist. Type locality: Western Mediterranean.

#### Eunoe assimilis

McIntosh, 1924

##### Notes

Questionable status. In the Mediterranean only reported from Greece (NCMR 1992). Originally described from South Africa.

#### Eunoe nodosa

(M. Sars, 1861)

##### Notes

Species complex. The exact distribution of the species in the Mediterranean is still unknown. Frequently reported from Greece in ecological lists but Barnich and Fiege (2003) consider their record of the species from the Western Mediterranean to be the first from the region. *Barnich and Fiege (2010)* state the distribution of the species to be "Arctic Ocean, North Atlantic and North Pacific". *Eunoe nodosa* has recently been shown to constitute a species complex comprising at least two cryptic species in the Arctic Ocean (Carr et al. 2011); whether the Greek specimens belong to either of these or to a different species is currently unknown.

#### Gattyana cirrhosa

(Pallas, 1766)

##### Notes

Questionable status. In the Mediterranean only reported from Greece (Bogdanos and Satsmadjis 1983, Bogdanos and Diapoulis 1984, NCMR 1989), Spain (Desbruyères et al. 1973) and Italy (Castelli et al. 2008) otherwise known from boreal regions and cold water areas along the French and Spanish Atlantic coasts (Dauvin et al. 2006, Núñez et al. 2012); its presence in the Mediterranean could not yet be confirmed (Barnich and Fiege 2003). It is easily confused with *Harmothoe antilopes* McIntosh, 1876, a species with similar elytral structures but different notochaetae (Barnich and Fiege 2000a).

#### Harmothoe antilopes

McIntosh, 1876

#### Harmothoe areolata

(Grube, 1860)

##### Notes

Type locality: Mediterranean (Gulf of Naples).

#### Harmothoe extenuata

(Grube, 1840)

Harmothoe extenuata (Grube, 1840) | *Lagisca extenuata* (Grube, 1840)

##### Notes

Easily confused with other species of *Harmothoe*, literature records from the Mediterranean and North-East Atlantic should be treated with care (Barnich and Fiege 2000a).

#### Harmothoe fraserthomsoni

McIntosh, 1897

##### Notes

Two specimens of Arvanitidis (1994) from the Aegean station 45 were re-identified as *Harmothoe impar* (Johnston 1839) and *Harmothoe imbricata* (Linnaeus, 1767) (Barnich and Fiege 2000a).

#### Harmothoe gilchristi

Day, 1960

##### Notes

Reported from Greece by Barnich and Fiege (2000a) based on two unidentified specimens from the 1964 Calypso expedition. However, *Harmothoe* species are easily confused and *Harmothoe gilchristi* could be underreported (Barnich and Fiege 2000a).

#### Harmothoe imbricata

(Linnaeus, 1767)

Harmothoe imbricata (Linnaeus, 1767)

##### Notes

Species complex. At least 6 different putative cryptic species – of which two occur in the Atlantic, four in the Arctic and three in the Pacific – were found by Carr et al. (2011). Nygren et al. (2010b) showed that different colour morphs in Scandinavia all belong to the same species. Whether the Mediterranean specimens belong to the same species as those from northern Europe is currently unknown.

#### Harmothoe impar

(Johnston, 1839)

Harmothoe impar (Johnston, 1839) | *Harmothoe reticulata* (Claparède, 1870)

##### Notes

The description of *Harmothoe impar* by Fauvel (1923) partly refers to *Harmothoe pagenstecheri* Michaelsen, 1896. Thus, specimens identified using Fauvel's key could belong to either species (Barnich and Fiege 2000a). *Harmothoe impar* was confirmed from Greece by Barnich and Fiege (2000a). Type locality: Mediterranean (Gulf of Naples).

#### Harmothoe longisetis

(Grube, 1863)

##### Notes

The description of *Harmothoe longisetis* of Fauvel (1923) partly refers to *Harmothoe glabra* (Malmgren, 1866) (Barnich and Fiege 2003, Barnich and Fiege 2009). Thus, records identified using Fauvel's key could belong to either species. Recent records (e.g. Chatzigeorgiou et al. 2016) were identified using the key and descriptions by Barnich and Fiege (2003). Type locality: Mediterranean (Adriatic).

#### Harmothoe spinifera

(Ehlers, 1864)

##### Notes

Type locality: Mediterranean (Rijeka Bay, Adriatic).

#### Lepidasthenia elegans

(Grube, 1840)

##### Notes

Type locality: Mediterranean.

#### Lepidasthenia maculata

Potts, 1910

##### Notes

Questionable status. Easily confused with *Lepidasthenia brunnea* Day, 1960, therefore Barnich and Fiege (2003) consider the records of the species in Europe doubtful. The authors also refer the description of *Lepidasthenia maculata* by Fauvel (1923) to *Lepidasthenia brunnea*, thus it is likely that specimens identified with Fauvel's key in fact belong to *Lepidasthenia brunnea*.

#### Lepidonotus clava

(Montagu, 1808)

#### Lepidonotus squamatus

(Linnaeus, 1758)

##### Notes

Questionable status, species complex. At least two putative cryptic species exist in waters around Canada (Carr et al. 2011). Reported from Greece by NCMR (1995) and Conides et al. (1999) but the species is easily confused with *Lepidonotus clava* (Montagu, 1808) or *Lepidonotus tenuisetosus* (Gravier, 1902) (Barnich and Fiege 2003). Records from the Adriatic could not be confirmed (Mikac 2015).

#### Lepidonotus tenuisetosus

(Gravier, 1902)

##### Ecological interactions

###### Native status

Non-native (casual)

##### Notes

Reported from Greece by Chatzigeorgiou et al. (2016). Originally from the Red Sea, in the Mediterranean also known from Israel and Egypt (Barnich and Fiege 2003), Turkey (Çınar et al. 2014) and the Adriatic (Mikac 2015).

#### Macellicephala laubieri

Reyss, 1971

##### Notes

Two specimens from Greece in the collections of the Senckenberg Museum (SMF 10528, 39°14'59.4"N, 23°42'31.8"E, 1208–1253 m depth, coll. date 1998-01-01, det. R. Barnich; SMF 10529, 39°14'59.4"N, 23°43'25.8"E; 1208–1253 m depth, coll. date 1997-12-31, det R. Barnich). Type locality: Mediterranean (deep waters North-East off the Balearic Islands).

#### Malmgrenia andreapolis

McIntosh, 1874

Harmothoe lunulata var. andreapolis McIntosh, 1874 | *Malmgrenia andreapolis* McIntosh, 1874| *Malmgreniella andreapolis* (McIntosh, 1874)

##### Notes

Reported from Greece by Arvanitidis (2000a) and Koulouri et al. (2015). In the Mediterranean also known from France (Barnich and Fiege 2000a), Italy (Castelli et al. 2008) and the Adriatic (Mikac 2015), otherwise distributed in the North East Atlantic (Barnich and Fiege 2001). Easily confused with *Malmgrenia castanea* McIntosh, 1876 and *Malmgrenia lunulata* (Delle Chiaje, 1830), thus literature records should be treated with care (Barnich and Fiege 2001).

#### Malmgrenia castanea

McIntosh, 1876

Malmgreniella castanea (McIntosh, 1876)

##### Notes

Easily confused with *Malmgrenia lunulata* (Delle Chiaje, 1830), thus literature records should be treated with care (Barnich and Fiege 2001).

#### Malmgrenia darbouxi

(Pettibone, 1993)

Malmgreniella darbouxi Pettibone, 1993

##### Notes

Reported from Greece by Arvanitidis (2000a) based on a single specimen. Type locality: Mediterranean (Adriatic).

#### Malmgrenia ljungmani

(Malmgren, 1867)

Harmothoe ljungmani (Malmgren, 1867) | *Malmgreniella ljungmani* (Malmgren, 1867)

#### Malmgrenia lunulata

(Delle Chiaje, 1830)

Harmothoe lunulata (Delle Chiaje, 1830) | *Malmgreniella lunulata* (Delle Chiaje, 1830)

##### Notes

Easily confused with congeners, thus literature records should be treated with care (Barnich and Fiege 2001). Type locality: Mediterranean (Gulf of Naples).

#### Paradyte cf. crinoidicola

(Potts, 1910)

##### Ecological interactions

###### Native status

Non-native (questionable)

##### Notes

Only recorded twice from the Mediterranean: Barnich and Fiege (2003) examined an old specimen in bad condition from the 1964 Calypso expedition from near Kassos Island (Greek Aegean) and identified it as *Paradyte* cf. crinoidicola. It differs from *Paradyte crinoidicola* as described by Pettibone (1969) in having blunt notochaetal tips and lower neurochaetae with straight distal regions. The authors do not rule out the possibility that these differences represent intraspecific variations and that *Paradyte crinoidicola* is indeed present in the Mediterranean (in which case it should be regarded as a Lessepsian migrant). Another record by Radić (2009) from the Adriatic as *Paradyte crinoidicola* is considered questionable by Mikac (2015) who, based on the uncertainty of the Greek record, considers the species' presence in the Mediterranean doubtful.

#### Polaruschakov reyssi

Pettibone, 1976

##### Notes

Reported from Greece by Barnich and Fiege (2003). Type locality: Mediterranean (submarine canyon off Banyuls-sur-Mer, France).

#### Polynoe scolopendrina

Savigny, 1822

##### Notes

Reported from Greece by Bogdanos et al. (2002). In the Mediterranean known from France (Bellan 1963), Spain (Campoy 1982), Italy (Castelli et al. 2008) and the Adriatic (Mikac 2015).

#### Subadyte pellucida

(Ehlers, 1864)

Adyte pellucida (Ehlers, 1864) | *Scalisetosus fragilis* (Claparède, 1868) | *Scalisetosus pellucidus* (Ehlers, 1864) | *Subadyte pellucida* (Ehlers, 1864)

##### Notes

Type locality: Mediterranean (Adriatic).

### Protodrilidae Hatschek, 1888

#### Lindrilus flavocapitatus

(Uljanin, 1877)

Protodrilus flavocapitatus (Uljanin, 1877)

##### Notes

Reported from Greece by Koukouras (1979) and Dounas (1988). In the Mediterranean also known from Italy (Castelli et al. 2008) and France (Martínez et al. 2017), otherwise mainly distributed in the Black Sea. Many genus-level records of protodrilids exist in literature and in unpublished datasets from Greece; therefore it is likely that *Lindrilus flavocapitatus* is more widespread in Greece than currently known.

### Sabellariidae Johnston, 1865

#### Lygdamis muratus

(Allen, 1904)

Lygdamis muratus (Allen, 1904) | *Pallasia murata* Allen, 1904

#### Phalacrostemma cidariophilum

Marenzeller, 1895

##### Notes

Originally described from various locations in Greece (detailed stations and coordinates are listed in von Marenzeller (1902)). Subsequently reported from Greece by Bellan (1964b).

#### Sabellaria alcocki

Gravier, 1906

Sabellaria alcocki Gravier, 1906 | *Sabellaria spinulosa alcocki* Gravier, 1906

##### Notes

Questionable status. The specimens and description of Sabellaria spinulosa var. alcocki by Fauvel (1927) were examined by da Cunha Lana and Gruet (1989) and found to contain several species, some belonging to *Sabellaria wilsoni* Lana & Gruet, 1989. Thus, specimens identified using Fauvel's key probably belong to other species. Lezzi et al. (2015) suggest that specimens recorded as *Sabellaria alcocki* in the Mediterranean belong to juveniles of *Sabellaria spinulosa* (Leuckart, 1849) and that *Sabellaria alcocki* is absent from the region. Schimmenti et al. (2015) show through molecular analyses that specimens identified as *Sabellaria alcocki* from Italy fully matched the cytochrome c oxidase subunit I (COI) sequences of *Sabellaria spinulosa*.

#### Sabellaria eupomatoides

Augener, 1918

##### Notes

Questionable status. In the Mediterranean only reported from Greece (Nicolaidou and Papadopoulou 1989). Distributed in the South-East Atlantic along the coasts of Africa.

#### Sabellaria spinulosa

(Leuckart, 1849)

##### Notes

Could comprise records of *Sabellaria alcocki* Gravier, 1906 (see remarks there).

### Sabellidae Latreille, 1825

#### Acromegalomma vesiculosum

(Montagu, 1813)

Branchiomma vesiculosum (Montagu, 1813) | *Megalomma vesiculosum* (Montagu, 1813)

##### Notes

Questionable status. Giangrande and Licciano (2008) re-examined several specimens previously identified as *Megalomma vesiculosum* from the Adriatic and Tyrrhenian Sea and Mediterranean Spain and found all of them to belong to *Megalomma lanigera* (Grube, 1846) (now *Acromegalomma lanigerum*, see Gil and Nishi 2017). They conclude that *Megalomma vesiculosum* is absent from the Mediterranean and that all Mediterranean material of *Megalomma vesiculosum* should be assigned to *Megalomma lanigera*.

#### Amphicorina armandi

(Claparède, 1864)

Amphicorina armandi (Claparède, 1864) | *Oridia armandi* (Claparède, 1864) | *Oriopsis armandi* (Claparède, 1864)

##### Notes

Type locality: Mediterranean (Port-Vendres, France).

#### Amphiglena mediterranea

(Leydig, 1851)

##### Notes

Type locality: Mediterranean.

#### Bispira fabricii

(Krøyer, 1856)

Bispira fabricii (Krøyer, 1856) | *Sabella fabricii* Krøyer, 1856

##### Notes

Frequently reported from the Mediterranean, but Knight-Jones and Perkins (1998) consider the species to be absent from the Mediterranean and restricted to Arctic areas.

#### Bispira melanostigma

(Schmarda, 1861)

Bispira bipunctata (Baird, 1865) | *Sabella bipunctata* Baird, 1865

##### Notes

Questionable status. Mediterranean records are rare and considered questionable (Giangrande 1989). Knight-Jones and Perkins (1998) likewise consider Mediterranean specimens to belong to other species (e.g. the record by Iroso (1921) probably is a juvenile *Bispira mariae* (Lo Bianco, 1893)) and restrict the distribution of the species to the wider Caribbean area.

#### Bispira volutacornis

(Montagu, 1804)

##### Notes

Knight-Jones and Perkins (1998) consider the distribution of the species insufficiently known but possibly restricted to the Atlantic coasts of Europe; several Mediterranean records could belong to other species.

#### Branchiomma bairdi

(McIntosh, 1885)

##### Ecological interactions

###### Native status

Non-native (casual)

##### Notes

Reported from Greece by Corsini-Foka et al. (2015). A West Atlantic / East Pacific species which has been reported in the Mediterranean from Italy (Giangrande et al. 2012), Malta (Arias et al. 2013b), Turkey (Çınar et al. 2014) and Tunisia (Khedhri et al. 2017).

#### Branchiomma bombyx

(Dalyell, 1853)

Branchiomma bombyx (Dalyell, 1853) | *Dasychone bombyx* (Dalyell, 1853)

#### Branchiomma luctuosum

(Grube, 1870)

##### Ecological interactions

###### Native status

Non-native (established)

##### Notes

Reported from Greece by Arvanitidis (2000a) based on a single specimen, found again afterwards (HCMR, unpublished data). A Red Sea species has been reported in the Mediterranean from Italy (Licciano and Giangrande 2008), Spain (El Haddad et al. 2009) and Turkey (Çınar et al. 2014).

#### Branchiomma lucullanum

(Delle Chiaje, 1828)

Branchiomma lucullanum (Delle Chiaje, 1828) | *Dasychone lucullana* (Delle Chiaje, 1828)

##### Notes

Type locality: Mediterranean (Gulf of Naples).

#### Chone duneri

Malmgren, 1867

##### Notes

Questionable status. Tovar-Hernández et al. (2007) and Tovar‐Hernández (2007) revise several species of *Chone* and restrict *Chone duneri* to the Arctic Ocean. Mediterranean specimens recorded as *Chone duneri* probably all belong to *Dialychone dunerificta* Tovar-Hernández, Licciano & Giangrande, 2007. According to Wasson et al. (2017), the genus *Chone* is absent from the Mediterranean.

#### Chone filicaudata

Southern, 1914

Chone filicaudata Southern, 1914 | *Paradialychone filicaudata* (Southern, 1914)

##### Notes

Questionable status. Until recently, the only known species in the genus *Chone* with a pygidial cirrus used to be *Chone filicaudata*; consequently all specimens possessing it were assigned to this species (Tovar-Hernández et al. 2007). However, nowadays more species with this character are known and historic records could belong to any of these species possessing a pygidial cirrus. *Chone filicaudata* is frequently reported from the Mediterranean but without accompanying descriptions or illustrations. The only available illustration by Giangrande (1992) does not correspond to the characters of the type material of *Chone filicaudata* (Tovar-Hernández et al. 2007). The species is currently believed to be restricted to the North-East Atlantic (Tovar-Hernández et al. 2007, Wasson et al. 2017); Mediterranean specimens likely belong to *Dialychone usticensis* (Giangrande, Licciano & Castriota, 2006).

#### Claviramus candelus

(Grube, 1863)

Jasmineira candela (Grube, 1863)

##### Notes

Reported from Greece by Antoniadou et al. (2004). Type locality: Mediterranean (Adriatic).

#### Desdemona ornata

Banse, 1957

##### Ecological interactions

###### Native status

Non-native (casual)

##### Notes

Reported from Greece by Panagopoulos and Nikolaidou (1989). Originally from the Indo-Pacific region, in the Mediterranean also known from Italy (Castelli et al. 2008) and the Adriatic (Mikac 2015). Present in the adjacent Sea of Marmara (Çınar et al. 2014).

#### Dialychone acustica

Claparède, 1870

Chone acustica (Claparède, 1870) | *Dialychone acustica* Claparède, 1870

##### Notes

Type locality: Mediterranean (Gulf of Naples).

#### Dialychone arenicola

(Langerhans, 1881)

Chone arenicola Langerhans, 1881

##### Notes

Reported from Greece by NCMR (1997) in the Mediterranean also known from Italy (Castelli et al. 2008) and Turkey (Çınar et al. 2014), otherwise known from the Atlantic Ocean (Madeira).

#### Dialychone collaris

(Langerhans, 1881)

Chone collaris Langerhans, 1881 | *Dialychone collaris* (Langerhans, 1881)

#### Dialychone longiseta

(Giangrande, 1992)

Chone longiseta Giangrande, 1992

##### Notes

Reported from Greece by Simboura (1996) and Zenetos et al. (1997) (based on the same specimens) and Chatzigeorgiou et al. (2016). Identification of the former specimens confirmed by A. Giangrande (pers. comm. in Simboura 1996). Type locality: Mediterranean (Gulf of Taranto, Ionian Sea).

#### Euchone capensis

Day, 1961

##### Notes

Questionable status. In the Mediterranean reported from Greece (Arvanitidis 2000a), Turkey (Çınar et al. 2014) and Italy (Cognetti-Varriale, unpublished data in Giangrande and Licciano 2006) However, Giangrande and Licciano (2006) consider the presence of *Euchone capensis* in the Mediterranean questionable, as the species is considered to be restricted to South Africa and the few reports of the species from the Mediterranean have never been verified.

#### Euchone pararosea

Giangrande & Licciano, 2006

##### Notes

Reported from Greece by Katsiaras and Simboura (2015). Type locality: Mediterranean (South Tyrrhenian Sea).

#### Euchone rosea

Langerhans, 1884

##### Notes

According to Giangrande and Licciano (2004) the species usually occurs on coralligenous habitats and soft-bottom records in the Mediterranean could belong to other species. However, Mikac (2015) confirms the species from soft bottoms in the North Adriatic.

#### Euchone rubrocincta

(Sars, 1862)

#### Euchone southerni incisa

Banse, 1970

##### Notes

Questionable status. In the Mediterranean only reported from Greece (Arvanitidis 2000a) based on a single specimen. Giangrande and Licciano (2004) do not list the species amongst the Mediterranean species of *Euchone*. Originally described from the Islas Malvinas, South Atlantic.

#### Euratella salmacidis

(Claparède, 1869)

Euratella salmacidis (Claparède, 1869) | *Laonome salmacidis* Claparède, 1869

##### Notes

Type locality: Mediterranean (Gulf of Naples).

#### Hypsicomus stichophthalmos

(Grube, 1863)

Hypsicomus stichophthalmos (Grube, 1863) | *Potamilla stichophthalmos* (Grube, 1863)

##### Notes

Type locality: Mediterranean.

#### Jasmineira caudata

Langerhans, 1880

#### Jasmineira elegans

Saint-Joseph, 1894

#### Laonome kroyeri

Malmgren, 1866

#### Myxicola infundibulum

(Montagu, 1808)

##### Notes

Species complex. *Myxicola infundibulum* is a taxon with a long list of synonyms and has until recently been considered cosmopolitan (Giangrande et al. 2012). However, specimens from different parts of the world (West Atlantic, Mediterranean, Pacific Canada) show genetic and morphological differences (Carr et al. 2011). Giangrande et al. 2012 found specimens of *Myxicola* from the Gulf of Naples and Lake Faro to morphologically differ from specimens of *Myxicola infundibulum* from the type locality in the North Adriatic Sea. It is therefore possible that the species constitutes a complex of cryptic species even in the Mediterranean.

#### Notaulax phaeotaenia

(Schmarda, 1861)

Hypsicomus phaeotaenia (Schmarda, 1861) | *Notaulax phaeotenia* (Schmarda, 1861)

##### Notes

*Notaulax phaeotaenia* has a long list of synonyms, as a result, the species has been reported from worldwide locations. However, many of these synonyms probably constitute valid and distinct species (Perkins 1984).

#### Parasabella langerhansi

(Knight-Jones, 1983)

Demonax langerhansi Knight-Jones, 1983

##### Notes

Reported from Greece by Arvanitidis (2000a) and Kitsos et al. (2005). In the Mediterranean also known from Turkey (Çınar et al. 2014) and the Adriatic (Mikac 2015), otherwise distributed along the Atlantic coasts of Europe and in Madeira (Giangrande 1994).

#### Parasabella saxicola

(Grube, 1861)

Demonax brachychona (Claparède, 1870)

##### Notes

Knight-Jones et al. (1991) synonymise *Demonax saxicola* and *Demonax brachychona* (Claparède, 1870) but erroneously use *Demonax brachychona* as the preferred name, despite Grube's name being older and thus having priority. Tovar‐Hernández and Harris (2010) re-instate the use of the name *Demonax saxicola* over *Demonax brachychona* and introduce *Parasabella* Bush, 1905 as a replacement name for *Demonax* Kinberg, 1867 (which is pre-occupied by the beetle genus *Demonax* Thomson, 1860). Type locality: Mediterranean (Adriatic).

#### Parasabella tenuicollaris

(Grube, 1870)

Demonax tenuicollaris (Grube, 1870)

##### Notes

Reported from Greece by Arvanitidis (1994). Type locality: Mediterranean (Adriatic).

#### Potamilla torelli

(Malmgren, 1866)

##### Notes

Questionable status. Knight-Jones (1983) re-examined the specimens of *Potamilla torrelli* of Fauvel (1927) and referred them to *Demonax brachychona* (Claparède, 1870) (now accepted as *Parasabella saxicola* (Grube, 1861). Thus, it is likely that all specimens under the name *Potamilla torelli* which were identified using Fauvel's work belong to *Potamilla saxicola*. Species of *Demonax* (now *Parasabella)* were usually overlooked or misidentified during the last century due to a very narrow description of the genus, as specimens with broadly hooded inferior thoracic chaetae used to be referred to *Potamilla*; consequently, records of *Potamilla torelli* from the Mediterranean are probably misidentifications Giangrande (1994). The presence of *Potamilla torelli* in the Mediterranean is furthermore doubted by Knight-Jones et al. (1991) who consider it to be distributed in Arctic regions.

#### Pseudopotamilla reniformis

(Bruguière, 1789)

Potamilla reniformis (Bruguière, 1789)| *Pseudopotamilla reniformis* (Bruguière, 1789)

##### Notes

Questionable status. Knight-Jones et al. (2017) investigate specimens of *Pseudopotamilla reniformis* from various locations in Europe, restrict the distribution of *Pseudopotamilla reniformis* to cold northern waters and re-instate *Pseudopotamilla saxicava* (Quatrefages 1866) for specimens from temperate and tropical waters, including the Mediterranean, noting that the description of *Pseudopotamilla reniformis* by Fauvel (1927) contains figures of both *Pseudopotamilla reniformis* and *Pseudopotamilla saxicava*. Greek records of *Pseudopotamilla reniformis* probably belong to *Pseudopotamilla saxicava*.

#### Sabella discifera

Grube, 1874

Megalomma linaresi (Rioja, 1917) | *Sabella discifera* Grube, 1874

##### Notes

Reported from Greece by Arvanitidis (1994) as *Sabella discifera*, by Fassari (1982) as *Megalomma linaresi*. Type locality of *Sabella discifera*: Mediterranean (Adriatic).

#### Sabella pavonina

Savigny, 1822

#### Sabella spallanzanii

(Gmelin, 1791)

Sabella spallanzanii (Gmelin, 1791) | *Spirographis spallanzanii* (Viviani, 1805)

##### Notes

Type locality: Mediterranean (neotype from Malta).

### Saccocirridae Czerniavsky, 1881

#### Saccocirrus papillocercus

Bobretzky, 1872

##### Notes

The genus *Saccocirrus* was recently split into the two genera *Saccocirrus* Bobretzky, 1872 (without pharynx, mainly occurring in the Mediterranean and along European coasts) and *Pharyngocirrus* Di Domenico, Martínez, Lana and Worsaee, 2014 (with pharynx, in tropical latitudes) based on morphological and molecular analyses (Di Domenico et al. 2014).

### Scalibregmatidae Malmgren, 1867

#### Asclerocheilus capensis

Day, 1963

##### Notes

Questionable status. In the Mediterranean only reported from Greece (NCMR 1992, Simboura 1996). Distributed in the tropical and South Atlantic and the Indian Ocean.

#### Asclerocheilus intermedius

(Saint-Joseph, 1894)

##### Notes

Reported from Greece by Fassari (1982) and Arvanitidis (1994). In the Mediterranean also known from Cyprus (Çınar 2005), Turkey (Çınar et al. 2014) and the Adriatic (Mikac 2015), otherwise distributed in the Atlantic.

#### Hyboscolex longiseta

Schmarda, 1861

##### Ecological interactions

###### Native status

Non-native (questionable)

##### Notes

Questionable status. Reported from Greece by Fassari (1982). In the Mediterranean also known from Tunisia (Cantone et al. 1978) and Italy (Castelli et al. 2008), otherwise distributed in the Southern Hemisphere (Kudenov and Blake 1978). Its presence in the Mediterranean is considered doubtful by Arvanitidis (1994) and Simboura et al. (2010).

#### Polyphysia crassa fauveli

(Laubier, 1959)

##### Notes

Reported from Greece by Simboura (1996) as *Polyphysia crassa* (Ørsted, 1843) but belonging in fact to the Mediterranean subspecies *Polyphysia crassa fauveli*; *Polyphysia crassa* is restricted to northern Europe (Kudenov and Blake 1978). In some unpublished datasets from Greece, the name *Lipobranchus jeffreysii* (McIntosh, 1869) is used. Støp-Bowitz (1945) considers *Lipobranchus jeffreysii*, the type of *Lipobranchius*, to be the juvenile of *Polyphysia crassa* (see also Kudenov and Blake 1978). Nevertheless, *Lipobranchus jeffreysii* continues to be listed in some works as a valid species (e.g. Hartmann-Schröder 1996).

#### Scalibregma celticum

Mackie, 1991

#### Scalibregma inflatum

Rathke, 1843

##### Notes

Species complex. *Scalibregma inflatum*, often considered cosmopolitan, actually comprises a number of very similar or sibling species (e.g. Kudenov and Blake 1978). In Europe, at least four species of *Scalibregma* are present (Mackie 1991). It is likely that some older records of *Scalibregma inflatum* from Greece belong to *Scalibregma celticum* Mackie, 1991 (Simboura 1996).

#### Sclerocheilus minutus

Grube, 1863

##### Notes

Type locality: Mediterranean (Croatia).

### Serpulidae Rafinesque, 1815

#### Apomatus ampulliferus

Philippi, 1844

Apomatus ampulliferus Philippi, 1844 | *Protula ampullifera* (Philippi, 1844)

##### Notes

Type locality: Mediterranean (Gulf of Naples).

#### Apomatus similis

Marion & Bobretzky, 1875

##### Notes

Type locality: Mediterranean (Marseille).

#### Bathyvermilia langerhansi

(Fauvel, 1909)

##### Notes

Questionable status. Reported from Greece by Tselepides (1992), but its presence in the Mediterranean is questioned by Ben-Eliahu and Fiege (1996), as Zibrowius (1973a) had studied all Mediterranean specimens available to him and found that they belong in fact to other species.

#### Bushiella (Jugaria) granulata

(Linnaeus, 1767)

Jugaria granulata (Linnaeus, 1767)

##### Notes

Questionable status. In the Mediterranean only reported from Greece (Christomanos 1963). Commonly distributed in boreal waters (Jirkov 2001).

#### Ditrupa arietina

(O. F. Müller, 1776)

Ditrupa arietina (O.F. Müller, 1776) | *Ditrupa subulata* (Deshayes, 1825) in Berkeley, 1835

##### Notes

Hartley (2014) points out different ecological preferences between populations of *Ditrupa arietina* in the Mediterranean and in the Atlantic and a sudden increase in population densities in the Western Mediterranean in the late 1980s, indicating the possibility of an unknown cryptic invasive species.

#### Ficopomatus enigmaticus

(Fauvel, 1923)

Ficopomatus enigmaticus (Fauvel, 1923) | *Mercierella enigmatica* Fauvel, 1923

##### Ecological interactions

###### Native status

Non-native (established)

##### Notes

Species complex. A species with an unknown native range but believed to be an introduced species in shallow waters worldwide (Styan et al. 2017) and frequently reported from Greece. Styan et al. (2017), using molecular methods, find at least three putative cryptic species of *Ficopomatus enigmaticus* in Australia.

#### Filograna implexa

Berkeley, 1835

##### Notes

Reported from Greece by Arvanitidis (1994) and Chatzigeorgiou et al. (2016). The relationships within the species of the *Filograna* / *Salmacina* complex and the characters used to distinguish species and genera are still a matter of debate (Nogueira and ten Hove 2000). *Filograna* is usually considered operculate and *Salmacina* non-operculate, but mixed colonies have been found, questioning the validity of the character. Fauvel (1927) considers the three taxa *Filograna implexa*, *Salmacina dysteri* (Huxle, 1855) and *Salmacina incrustans* Claparède, 1870 to be separate species, a view which is also tentatively followed by Nogueira and ten Hove (2000) until more information becomes available. They recommend using locally applied names for each of the species. We follow this view here and list all three species as separate, pending further taxonomic investigation. Greek specimens were identified using the key of Bianchi (1981).

#### Filogranula annulata

(O. G. Costa, 1861)

##### Notes

Reported from Greece by Zibrowius (1979) and Gerovasileiou et al. (2015). Type locality: Mediterranean.

#### Filogranula calyculata

(O. G. Costa, 1861)

#### Filogranula gracilis

Langerhans, 1884

#### Filogranula stellata

(Southward, 1963)

#### Hyalopomatus variorugosus

Ben-Eliahu & Fiege, 1996

##### Notes

Reported from Greece by Ben-Eliahu and Fiege (1996) (several paratypes from deep waters off Crete) and by Sanfilippo et al. (2017) as Hyalopomatus cf. variorugosus from two caves of Lesvos Island. Type locality: Mediterranean (Levantine Basin).

#### Hydroides brachyacantha

Rioja, 1941

##### Ecological interactions

###### Native status

Non-native (casual)

##### Notes

Species complex. Reported from Greece by Ulman et al. (2017). In the Mediterranean also known from Israel (Ben-Eliahu 1991a), Turkey (Çınar 2006) and Spain (Ulman et al. 2017). Probably originating from the Mexican Pacific (Ulman et al. 2017), but the species has been shown to constitute a complex of cryptic and pseudo-cryptic species (Bastida-Zavala and ten Hove 2003, Sun et al. 2016). The identity of Mediterranean specimens, their introduction status in the Mediterranean and the potential native range cannot be determined without molecular methods.

#### Hydroides dianthus

(Verrill, 1873)

##### Notes

Species complex. *Hydroides dianthus* was originally described from Connecticut and long believed to be non-native in the Mediterranean (Zibrowius 1973b, Zenetos et al. 2017). A recent study by Sun et al. (2017) shows, however, that *Hydroides dianthus* could originate from the Mediterranean, as the species shows the highest genetic diversity in the Mediterranean (nine out of ten haplotypes present, as opposed to only two haplotypes in the USA). The authors recovered also a putative cryptic species, Hydroides cf. dianthus, probably originating from the Gulf of Mexico and apparently recently introduced to the Black Sea.

#### Hydroides dirampha

Mörch, 1863

Hydroides dirampha Mörch, 1863 | *Hydroides lunulifera* (Claparède, 1870)

##### Ecological interactions

###### Native status

Non-native (established)

##### Notes

Originally described from the Caribbean, established throughout the Mediterranean (Zibrowius 1973b).

#### Hydroides elegans

(Haswell, 1883)

##### Ecological interactions

###### Native status

Non-native (established)

##### Notes

May be underreported in Greece, as specimens identified as *Hydroides norvegica* Gunnerus, 1768 from harbour and biofouling environments probably belong to *Hydroides elegans* (Zibrowius 1971, Arvanitidis 1994, ten Hove and Kupriyanova 2009). The species is considered introduced to the Mediterranean, although its centre of origin is still unknown (ten Hove 2003).

#### Hydroides helmata

(Iroso, 1921)

##### Notes

Reported from Greece by Arvanitidis (1994). Type locality: Mediterranean (Gulf of Naples).

#### Hydroides nigra

Zibrowius, 1971

##### Notes

Type locality: Mediterranean (Tarbaka, Tunisia).

#### Hydroides norvegica

Gunnerus, 1768

##### Notes

Zibrowius (1971) shows that the non-native species *Hydroides elegans* (Haswell, 1883) has replaced *Hydroides norvegica* in harbour fouling communities of the Mediterranean and that *Hydroides norvegica* is restricted to deeper waters. ten Hove and Kupriyanova (2009) suggest that all (sub)tropical records of *Hydroides norvegica* should be attributed to *Hydroides elegans*. In Greece, *Hydroides norvegica* has been reported both from harbour and shallow coastal communities as well as from deep waters below 500 m (e.g. Tselepides 1992). Its presence in the port of Heraklion and other Mediterranean ports has been recently confirmed (Ulman et al. 2017).

#### Hydroides pseudouncinata

Zibrowius, 1968

Hydroides pseudouncinata Zibrowius, 1968 | *Hydroides pseudouncinata pseudouncinata* Zibrowius, 1968

##### Notes

Type locality: Mediterranean (Marseille).

#### Hydroides stoichadon

Zibrowius, 1971

##### Notes

Reported from Greece by Simboura (1996) and Kitsos et al. (2005). Type locality: Mediterranean (off the French coast).

#### Janita fimbriata

(Delle Chiaje, 1822)

Janita fimbriata (Delle Chiaje, 1822) | Janita (Omphalopoma) fimbriata (Delle Chiaje, 1822) | *Omphalopomopsis fimbriata* (Delle Chiaje, 1822)

##### Notes

Type locality: Mediterranean (Gulf of Naples).

#### Janua heterostropha

(Montagu, 1803)

Janua (Dexiospira) pagenstecheri (Quatrefages, 1866) | *Janua pagenstecheri* (Quatrefages, 1866) | *Janua pagenstecheri gnonomica* (Bailey, 1969) | Spirorbis (Janua) gnomonicus Bailey, 1969| Spirorbis (Janua) pagenstecheri Quatrefages, 1866

#### Josephella marenzelleri

Caullery & Mesnil, 1896

#### Metavermilia acanthophora

(Augener, 1914)

Metavermilia acanthophora (Augener, 1914) | *Vermiliopsis acanthophora* Augener, 1914

##### Notes

Questionable status. In the Mediterranean only reported from Greece (Bogdanos and Nicolaidou 1985, Bogdanos and Satsmadjis 1987). Commonly distributed in the Indo-Pacific region (Kupriyanova et al. 2015).

#### Metavermilia multicristata

(Philippi, 1844)

Metavermilia multicristata (Philippi, 1844) | *Vermilia multicristata* Philippi, 1844

##### Notes

Type locality: Mediterranean.

#### Metavermilia taenia

Zibrowius, 1971

##### Notes

Questionable status. One specimen from Greece in the collections of the Senckenberg Museum (SMF 22101, 34°57'20.34"N, 26°11'04.62"E, 107–117 m depth, coll. date 2005-08-31, det. H Zibrowius, 2005). Identification uncertain, entry indicated with a question mark. No other Mediterranean records; originally described from off the Portuguese coast.

#### Neodexiospira pseudocorrugata

(Bush, 1905)

Neodexiospira pseudocorrugata (Bush, 1905) | Spirorbis (Janua) parvulus Bailey, 1969

##### Notes

Records likely also comprise those recorded under the name *Spirorbis corrugatus* which were identified using Fauvel (1927) (see also entry for *Spirorbis corrugatus* (Montagu, 1803) in Table 1 for detailed notes on taxonomy and nomenclature).

#### Nidificaria clavus

(Harris, 1968)

Nidificaria clavus (Harris, 1968) | Spirorbis (Pileolaria) clavus Harris, 1968

#### Pileolaria heteropoma

(Zibrowius, 1968)

Pileolaria heteropoma (Zibrowius, 1968) | Spirorbis (Pileolaria) heteropoma Zibrowius, 1968

##### Notes

Type locality: Mediterranean (Marseille).

#### Pileolaria militaris

Claparède, 1870

Pileolaria militaris Claparède, 1870| *Spirorbis mediterraneus* Caullery & Mesnil, 1896 | Spirorbis (Pileolaria) militaris (Claparède, 1870)

##### Notes

Type locality: Mediterranean (Gulf of Naples).

#### Placostegus crystallinus

non (Scacchi, 1836) sensu Zibrowius, 1968

##### Notes

Greek records all refer to the species as described by Zibrowius (1968). Type locality: Mediterranean (France).

#### Placostegus tridentatus

(Fabricius, 1779)

##### Notes

The species exhibits two different morphotypes in the Atlantic and the Mediterranean. Mediterranean specimens are smaller than Atlantic specimens and show differences in morphological details of the tube (for a detailed description see Sanfilippo (2003)).

#### Protis arctica

(Hansen, 1879)

##### Notes

Reported from deep waters of Greece and the Eastern Mediterranean by Ben-Eliahu and Fiege (1996).

#### Protolaeospira striata

(Quiévreux, 1963)

Protolaeospira (Protolaeospira) striata (Quiévreux, 1963) | *Protolaeospira striata* (Quiévreux, 1963) | Spirorbis (Marsipospira) striatus (Quiévreux, 1963)

#### Protula intestinum

(Lamarck, 1818)

Protula intestinum (Lamarck, 1818) | *Serpula graeca* Brullé, 1832

##### Notes

Type locality: Mediterranean.

#### Protula tubularia

(Montagu, 1803)

#### Salmacina dysteri

(Huxley, 1855)

##### Notes

Reported from Greece by Morri et al. (1999) and Bianchi and Morri (2000b). Reported from worldwide locations but probably constitutes a complex of species (Nogueira and ten Hove 2000). See also remarks under *Filograna implexa* Berkeley, 1835.

#### Salmacina incrustans

Claparède, 1870

##### Notes

See remarks under *Filograna implexa* Berkeley, 1835. Type locality: Mediterranean (Gulf of Naples).

#### Semivermilia agglutinata

(Marenzeller, 1893)

Semivermilia agglutinata (Marenzeller, 1893) | *Vermilia agglutinata* Marenzeller, 1893

##### Notes

The original description is based on specimens collected in Libya and Greece (near Kythira Island and north of Crete).

#### Semivermilia crenata

(O. G. Costa, 1861)

##### Notes

Type locality: Mediterranean.

#### Semivermilia cribrata

(O. G. Costa, 1861)

##### Notes

Reported from Greece by Ben-Eliahu and Fiege (1996); additionally two specimens from Greece in the collections of the Senckenberg Museum (SMF 21066, 37°39'48"N, 23°56'42"E, 23 m depth, coll. date July 1995; SMF 19682, near Corfu, ca. 20m depth, coll. date 1958-07-10, both det. H. Zibrowius). Type locality: Mediterranean.

#### Semivermilia pomatostegoides

(Zibrowius, 1969)

#### Semivermilia torulosa

(Delle Chiaje, 1822)

##### Notes

Type locality: Mediterranean (Gulf of Naples).

#### Serpula cavernicola

Fassari & Mòllica, 1991

##### Notes

Reported from Greece by Sanfilippo et al. (2017). Only dead specimens of *Serpula cavernicola* were found – although in several stations – in two caves of Lesvos Island. Type locality: Mediterranean (Sicily).

#### Serpula concharum

Langerhans, 1880

#### Serpula lobiancoi

Rioja, 1917

#### Serpula vermicularis

Linnaeus, 1767

Serpula vermicularis Linnaeus, 1767 | *Serpula vermicularis echinata* Gmelin, 1791

#### Simplaria pseudomilitaris

(Thiriot-Quiévreux, 1965)

Simplaria pseudomilitaris (Thiriot-Quiéreux, 1965) | Spirorbis (Pileolaria) pseudomilitaris Thiriot-Quiévreux, 1965)

##### Notes

Type locality: Mediterranean (Villefranche-sur-Mer).

#### Spiraserpula massiliensis

(Zibrowius, 1968)

Serpula massiliensis Zibrowius, 1968 | *Spiraserpula massiliensis* (Zibrowius, 1968)

##### Notes

Type locality: Mediterranean (Marseille).

#### Spirobranchus lamarcki

(Quatrefages, 1866)

Pomatoceros lamarckii (Quatrefages, 1866)

#### Spirobranchus polytrema

(Philippi, 1844)

Pomatostegus polytrema (Philippi, 1844) | *Spirobranchus polytrema* (Philippi, 1844)

##### Notes

Type locality: Mediterranean.

#### Spirobranchus tetraceros

(Schmarda, 1861)

##### Ecological interactions

###### Native status

Non-native (established)

##### Notes

Species complex with a confused taxonomic status, comprising one species in the Caribbean and several in the Indo-Pacific (Ben-Eliahu and ten Hove 2011). Reported from Greece by Ben-Eliahu (1991a) and Ulman et al. (2017). Originally from the Red Sea, in the Mediterranean also known from Egypt (Ben-Eliahu and Fiege 1996), Israel (Fishelson 2000), Cyprus (Çınar 2005), Turkey (Çınar et al. 2014) and Italy (Ulman et al. 2017).

#### Spirobranchus triqueter

(Linnaeus, 1758)

Pomatoceros triqueter (Linnaeus, 1758) | *Spirobranchus triqueter* (Linnaeus, 1758)

#### Spirorbis cuneatus

Gee, 1964

Spirorbis (Spirorbis) cuneatus Gee, 1964 | *Spirorbis cuneatus* Gee, 1964

#### Spirorbis marioni

Caullery & Mesnil, 1897

##### Ecological interactions

###### Native status

Non-native (established)

##### Notes

Reported from Greece by NCMR (1997) and Kitsos (2003). Originally described from Panama; known from harbours throughout the Mediterranean (Galil et al. 2008).

#### Vermiliopsis infundibulum

(Philippi, 1844)

Vermilia multivaricosa Mörch, 1863 | *Vermiliopsis infundibulum* (Philippi, 1844)

##### Notes

Type locality: Mediterranean.

#### Vermiliopsis labiata

(O. G. Costa, 1861)

Vermiliopsis labiata (O. G. Costa, 1861) | *Vermiliopsis richardi* Fauvel, 1909

##### Notes

Type locality: Mediterranean.

#### Vermiliopsis monodiscus

Zibrowius, 1968

##### Notes

Type locality: Mediterranean.

#### Vermiliopsis striaticeps

(Grube, 1862)

##### Notes

Type locality: Mediterranean.

#### Vinearia endoumensis

(Zibrowius, 1968)

Spirorbis (Pileolaria) endoumensis Zibrowius, 1968 | *Vinearia endoumensis* (Zibrowius, 1968)

##### Notes

Reported from Greece by Bailey (1969). Type locality: Mediterranean (Marseille).

#### Vinearia koehleri

(Caullery & Mesnil, 1897)

Spirorbis (Pileolaria) koehleri (Caullery & Mesnil, 1897) |*Vinearia koehleri* (Caullery & Mesnil, 1897)

##### Notes

Type locality: Mediterranean (La Ciotat, France).

### Siboglinidae Caullery, 1914

#### Lamellibrachia anaximandri

Southward, Andersen & Hourdez, 2011

##### Notes

Originally described from the Anaximander Seamount (Eastern Mediterranean), paratypes from the Olimpi mud volcano, south of Crete, ca. 2000 m depth. Previously recorded as *Lamellibrachia* sp. by Olu-Le Roy et al. (2004) from the Olimpi mud volcano and by Hughes and Crawford (2006) from the wreck of the SS Persia, south-east of Crete.

#### Siboglinum cf. carpinei

Ivanov, 1970

##### Notes

Reported from Greece by Southward et al. (2011) from the Olimpi mud volcano, south of Crete. Type locality: Mediterranean (west of Corsica).

### Sigalionidae Malmgren, 1867

#### Claparedepelogenia inclusa

(Claparède, 1868)

Claparedepelogenia inclusa (Claparède, 1868) | *Psammolyce inclusa* (Claparède, 1868)

##### Notes

Type locality: Mediterranean (Gulf of Naples).

#### Euthalenessa oculata

(Peters, 1854)

Euthalenessa oculata (Peters, 1854) | *Thalenessa dendrolepis* (Claparède, 1868)

##### Notes

Type locality: Mediterranean (Gulf of Naples).

#### Fimbriosthenelais minor

(Pruvot & Racovitza, 1895)

Fimbriosthenelais minor (Pruvot & Racovitza, 1895) | S*thenelais minor* Pruvot & Racovitza, 1895

##### Notes

Type locality: Mediterranean (Banyuls-sur-Mer).

#### Fimbriosthenelais zetlandica

(McIntosh, 1876)

Sthenelais papillosa Day, 1960

#### Heteropelogenia articulata

(Day, 1960)

Psammolyce articulata Day, 1960

##### Notes

Questionable status. In the Mediterranean only reported from Greece (Makra and Nicolaidou 2000). Distributed along the coasts of South Africa and in the Indian Ocean.

#### Labioleanira yhleni

(Malmgren, 1867)

Labioleanira yhleni (Malmgren, 1867) | *Leanira yhleni* Malmgren, 1867| *Sthenolepis yhleni* (Malmgren, 1867)

#### Neoleanira tetragona

(Ørsted, 1845)

##### Notes

Questionable status. Usually occurring in muddy sediments of deep-water habitats (down to 2200 m) and rarely recorded from the Mediterranean (Barnich and Fiege 2003). The two Greek records are from shallower waters: from max. 35 m depth (Chintiroglou 1987) and from coelenteron content of the anemone *Calliactis parasitica* (Couch, 1842) collected from depths down to 80 m (Chintiroglou and Koukouras 1991) and must therefore be considered questionable.

#### Pelogenia arenosa

(Delle Chiaje, 1830)

Pelogenia arenosa (Delle Chiaje, 1830) | *Psammolyce arenosa* (Delle Chiaje, 1830)

##### Notes

Type locality: Mediterranean (Gulf of Naples).

#### Pholoe inornata

Johnston, 1839

Pholoe inornata Johnston, 1839 | *Pholoe synophthalmica* Claparède, 1868

##### Notes

Recent phylogenetic analysis of the Aphroditiformia, taking into account morphological and molecular information, recovered species previously assigned to Pholoidae as nested within Sigalionidae (Norlinder et al. 2012, Gonzalez et al. 2017). Pholoidae were demoted to subfamiliar rank (Pholoinae) within Sigalionidae by Gonzalez et al. (2017).

#### Pholoe minuta

(Fabricius, 1780)

##### Notes

Questionable status. Barnich and Fiege (2003) doubt the presence of *Pholoe minuta* in the Mediterranean and suggest that records should be attributed to *Pholoe inornata* Johnston, 1839. *Pholoe minuta* is very likely absent from Greece. See notes under *Pholoe inornata* for information on higher classification.

#### Pholoides dorsipapillatus

(Marenzeller, 1893)

Pholoe dorsipapillata Marenzeller, 1893 | *Pholoides dorsipapillatus* (Marenzeller, 1893)

##### Notes

The original description is based on specimens collected in Egypt and Greece (harbour of Santorini). See notes under *Pholoe inornata* Johnston, 1839 for information on higher classification.

#### Pisione remota

(Southern, 1914)

Pisione remota (Southern, 1914) | *Praegeria remota* Southern, 1914

##### Notes

Species of *Pisione* are mainly distinguished on the basis of number and shape of male copulatory organs (Yamanishi 1998, Gonzalez et al. 2017b). Juvenile specimens are often erroneously assigned to *Pisione remota* (A. Martínez, pers. comm.) and the diversity of the genus in Europe might be underestimated (Martins et al. 2012b). As at least three species of *Pisione* are known to occur in the Mediterranean (Martins et al. 2012b), more than one species of the genus could in fact be present in Greece (R. Martins, pers. comm.).

#### Sigalion mathildae

Audouin & Milne Edwards in Cuvier, 1830

#### Sigalion squamosus

Delle Chiaje, 1830

##### Notes

Type locality: Mediterranean (Gulf of Naples).

#### Sthenelais boa

(Johnston, 1833)

Sthenelais boa (Johnston, 1833) | S*thenelais ctenolepis* Claparède, 1868

##### Notes

Type locality: Mediterranean (Gulf of Naples).

#### Sthenelais limicola

(Ehlers, 1864)

##### Notes

Type locality: Mediterranean (Croatia).

### Sphaerodoridae Malmgren, 1867

#### Sphaerodoridium claparedii

(Greeff, 1866)

Sphaerodoridium claparedii (Greeff, 1866) | *Sphaerodorum claparedii* Greeff, 1866

#### Sphaerodoridium minutum

(Webster & Benedict, 1887)

Sphaerodoropsis minuta (Webster & Benedict, 1887) | *Sphaerodoridium minutum* (Webster & Benedict, 1887) | *Sphaerodorum minutum* (Webster & Benedict, 1887)

#### Sphaerodorum gracilis

(Rathke, 1843)

Sphaerodorum flavum Ørsted, 1843

##### Notes

Questionable status. Reported from Greece by Goumenaki (2005). In the Mediterranean also known from Italy (Castelli et al. 2008), Spain (Serrano-Samaniego 2012) and the Adriatic (Mikac 2015). Present in the adjacent Sea of Marmara (Çınar et al. 2014). Otherwise distributed in the North Atlantic. While it is possible that the species occurs in Greece, it has so far only been reported in grey literature; its presence is therefore preliminarily considered questionable, pending confirmation.

### Spintheridae Augener, 1913

#### Spinther arcticus

(M. Sars, 1851)

Spinther miniaceus Grube, 1860

##### Notes

Several specimens from Greece in the collections of the Natural History Museum Vienna (8 specimens, Inv. No. 109, Acq. No. 929, Zante [=Zakynthos Island], acquisition date 1874; leg. E. v. Marenzeller; many specimens, Inv. No. 107, Acq. No. 12583, Zante [=Zakynthos Island], acquisition date 1887, det. R. Drasche). Type locality of *S. miniaceus*: Mediterranean (North Adriatic).

### Spionidae Grube, 1850

#### Aonidella cirrobranchiata

(Day, 1961)

Prionospio cirrobranchiata Day, 1961

##### Notes

Questionable status. In the Mediterranean only reported from Greece (Bogdanos and Satsmadjis 1983). Commonly distributed in West Atlantic and South-West Africa. Arvanitidis (1994) regards its presence in the Mediterranean as doubtful. Day (1961) and Day (1967) include it in the subgroup of *Prionospio* species with apinnate branchiae. Maciolek in her unpublished dissertation (Maciolek Blake 1983) and Lopez-Jamar (1989) moved it to a new genus (*Aonidella*) on the basis of other unique diagnostic characters (for more information see Maciolek 2000). Re-examination of the material would be required to investigate the possible assignment of the Greek record to a Prionospio (Minuspio) species with apinnate branchiae.

#### Aonides oxycephala

(Sars, 1862)

##### Notes

Frequently reported from Greece and other parts of the Mediterranean (Çınar 2005, Castelli et al. 2008, Ayari et al. 2009, Çınar et al. 2014, Mikac 2015). Originally described from the North Sea, reported from worldwide locations. However, Radashevsky (2015) questions its cosmopolitanism, noting the possibility of a series of sibling species.

#### Aonides paucibranchiata

Southern, 1914

##### Notes

Identifications of Greek specimens are based on descriptions of Fauvel (1927) and may have been easily confused with *Aonides oxycephala* (Sars, 1862) or other similar species. According to a note in Fauvel (1927), this small sized species could be a juvenile form of *Aonides oxycephala*, differing from it mainly by the smaller size, the number of gills and the smaller sized pygidial cirri, while the tridentate ventral hooks could be in fact a post-larval character, frequently observed in the Spionidae. Besides, the number of branchiae and the range of the ventral hooks may follow an intraspecific variation (Ramos 1976a, Johnson 1984 and personal observations from Greek material). According to F. Gravina and A. Somaschini (pers. comm.), it is a rare species.

#### Atherospio guillei

(Laubier & Ramos, 1974)

Polydora guillei Laubier & Ramos, 1974

##### Notes

Type locality: Mediterranean (Baie de Rosas, Spain).

#### Aurospio banyulensis

(Laubier, 1966)

Prionospio banyulensis Laubier, 1966

##### Notes

Type locality: Mediterranean (Banyuls-sur-Mer, France).

#### Boccardia polybranchia

(Haswell, 1885)

Boccardia polybranchia (Haswell, 1885) | Polydora (Boccardia) polybranchia Haswell, 1885

##### Notes

Originally described from Australia and reported from worldwide locations. However, Radashevsky (2015) notes that the characters of the material from type locality are poorly known and that the insufficient original description could have led to reports from worldwide locations. Therefore several records possibly have been confused or constitute undescribed species.

#### Boccardiella cf. ligerica

(Ferronnière, 1898)

##### Notes

Reported from Greece by Papazacharias (1991). In the Mediterranean also known from the Adriatic (Casellato and Stefanon 2008). Originally described from the East Atlantic.

#### Dipolydora armata

(Langerhans, 1880)

Dipolydora armata (Langerhans, 1880) | *Polydora armata* Langerhans, 1881

##### Notes

Originally described from Madeira, with a circumtropical distribution. However, reports of this species show a high polymorphism of specific characters (Radashevsky and Nogueira 2003). It has been suggested that at least two allopatric species are present under the name *Dipolydora armata* (Blake 1983). Bick (2002) distinguishes three morphological forms amongst Western Mediterranean material on the basis of notopodial spines, but Radashevsky and Nogueira (2003) attribute this variability to stages of asexual reproduction of the species.

#### Dipolydora blakei

(Maciolek, 1984)

##### Ecological interactions

###### Native status

Cryptogenic

##### Notes

Reported from Greece by Radashevsky and Simboura (2013) who re-examined specimens reported by Simboura (1996) and Zenetos et al. (1997) as *Polydora caulleryi* Mesnil, 1897. Originally described from New England, otherwise distributed in the North-West Atlantic and South America.

#### Dipolydora coeca

(Ørsted, 1843)

Dipolydora coeca (Ørsted, 1843)| *Polydora caeca* (Ørsted, 1843)

#### Dipolydora flava

(Claparède, 1870)

Dipolydora flava (Claparède, 1870) | *Polydora flava* Claparède, 1870

##### Notes

Type locality: Mediterranean (Gulf of Naples).

#### Dipolydora quadrilobata

(Jacobi, 1883)

Polydora quadrilobata Jacobi, 1883

##### Notes

Reported from Greece by Akoumianaki (2004). In the Mediterranean also known from the Adriatic (Mikac 2015). Reported also from the Black Sea, Romania (Kurt Şahin and Çınar 2012), otherwise distributed in the North Atlantic.

#### Dispio magnus

(Day, 1955)

##### Ecological interactions

###### Native status

Non-native (casual)

##### Notes

Reported from Greece by Simboura (1996). In the Mediterranean also known from the central basin (Zenetos et al. 2012). Originally described from South Africa.

#### Laonice cirrata

(M. Sars, 1851)

##### Notes

Questionable status. Species complex. At least two putative cryptic species exist in the Canadian Pacific (Carr et al. 2011). Records of *Laonice cirrata* in the Mediterranean may actually belong to *Laonice bahusiensis* Söderström 1920 (Mikac 2015, see Sikorski 2003 for differences between species). The former is restricted to circumpolar areas, the very similar *Laonice bahusiensis*, however, has a more southern distribution and is also present in the Central and Eastern Mediterranean (Sikorski 2003, Çınar et al. 2014). Fauvel (1927) synonymised *Laonice bahusiensis* with *Laonice cirrata*, including only *Laonice cirrata* in his key. *Laonice bahusiensis* may therefore be underreported or misidentified and all previous records of *Laonice cirrata* need to be investigated to confirm their identity.

#### Laubieriellus salzi

(Laubier, 1970)

Prionospio salzi Laubier, 1970

##### Notes

Reported from Greece by Simboura and Zenetos (2005) and HCMR (2011). Type locality: Mediterranean (Israel).

#### Malacoceros fuliginosus

(Claparède, 1870)

Malacoceros fuliginosus (Claparède, 1870) | *Scolelepis fuliginosa* (Claparède, 1870)

##### Notes

Species complex. Guerin and Kerambrun (1984) report three forms, on the basis of morphological, ecological and genetic characters. Two of these forms co-exist in the Mediterranean Sea, while the third was found only in the North East Atlantic. Type locality: Mediterranean Sea (Italy).

#### Malacoceros tetracerus

(Schmarda, 1861)

Malacoceros ciliatus (Keferstein, 1862) | *Scolelepis ciliata* (Keferstein, 1862)

#### Malacoceros vulgaris

Johnston, 1827

Malacoceros girardi Quatrefages, 1834 | *Malacoceros vulgaris Johnston, 1827 | Scolelepis girardii* (Quatrefages, 1834)

##### Notes

Reported from Greece by Dando et al. (1995) as *Malacoceros vulgaris*, but Arvanitidis (2000a) and Simboura and Nicolaidou (2001) use the name *Malacoceros girardi*. Pettibone (1963) synonymised *Malacoceros girardi* with *Malacoceros vulgaris* and only the latter is accepted as valid by several later authors (e.g. Dauvin et al. 2003, Delgado-Blas and Díaz-Díaz 2010, Mikac 2015). However, the name *Malacoceros girardi* has been recently used for records from the Mediterranean (e.g. Castelli et al. 2008, Dağli 2008, Çınar et al. 2014). Herein, we follow the suggested synonymy by Pettibone (1963), since no contradicting evidence has been presented in the literature.

#### Microspio mecznikowianus

(Claparède, 1869)

Microspio mecznikowianus (Claparède, 1869) | *Paraspio mecznikowianus* (Claparède, 1869)

##### Notes

Type locality: Mediterranean (Gulf of Naples).

#### Paraprionospio coora

Wilson, 1990

##### Ecological interactions

###### Native status

Cryptogenic

##### Notes

Reported from Greece by Simboura et al. (2010) who report some slight morphological deviations from the original description and Katsiaras and Simboura (2015). In the Mediterranean also known from Turkey (Yokoyama et al. 2010, Çınar et al. 2014), Spain (Zenetos et al. 2010), Tunisia (Zaâbi et al. 2012) and the Adriatic (Mikac 2015), otherwise known from the Pacific Ocean (Australia, Far East).

#### Paraprionospio pinnata

(Ehlers, 1901)

Paraprionospio pinnata (Ehlers, 1901) | *Prionospio pinnata* Ehlers, 1901

##### Notes

Questionable status. Several authors suggest that Mediterranean records of this species should be assigned to *Paraprionospio coora* (Yokoyama et al. 2010, Simboura et al. 2010, Mikac 2015).

#### Polydora ciliata

(Johnston, 1838)

##### Notes

Species complex. Mustaquim (1986) and Mustaquim (1988) provided behavioural (boring and tube-dwelling) and genetic evidence of two forms present under *Polydora ciliata*; morphological differences between the forms are also reported. Radashevsky and Pankova (2006), regarding European material, assign the tube-dwelling form to *Polydora ciliata* and the boring form to *Polydora calcarea* (Templeton, 1836). The presence of *Polydora ciliata* in the Mediterranean was questioned by Çinar et al. (2005) and Dağlı et al. (2011); but see also Mikac (2015). The former authors suggest that *Polydora ciliata* could have been confused with *Polydora cornuta* Bosc, 1802 or *Polydora agassizii* Claparède, 1869; a Mediterranean species synonymised with *Polydora ciliata* by Carazzi (1893), but resurrected by Radashevsky and Hsieh (2000). Until re-examination of the Greek material is possible, these records are retained under *Polydora ciliata* in this checklist.

#### Polydora cornuta

Bosc, 1802

##### Ecological interactions

###### Native status

Non-native (casual)

##### Notes

Species complex. Rice et al. (2008) provide molecular evidence of at least three putative cryptic species present in North America. Reported from Greece by Simboura et al. (2008). Originally described from South Carolina; in the Mediterranean also known from Spain (Tena et al. 2000), Turkey (Çınar et al. 2014) and from Adriatic lagoons (Bertasi 2016). Also reported from the Black Sea (Kurt Şahin and Çınar 2012, Radashevsky and Selifonova 2013).

#### Polydora hoplura

Claparède, 1868

##### Notes

Often found boring in oysters. Type locality: Mediterranean (Gulf of Naples). Sato-Okoshi et al. (2016), in agreement with the taxonomic observations of Radashevsky and Migotto (2016), provided molecular evidence that *Polydora uncinata* Sato-Okoshi, 1998 is a synonym of *Polydora hoplura*.

#### Polydora spongicola

Berkeley & Berkeley, 1950

##### Ecological interactions

###### Native status

Non-native (questionable)

##### Notes

Questionable status. In the Mediterranean only reported from Greece (Arvanitidis 2000a). Originally described from Western Canada, distributed in the East and West Pacific. The species is associated with sponges, a habitat for polychaetes which is little investigated in Greece.

#### Prionospio caspersi

Laubier, 1962

Prionospio caspersi Laubier, 1962 | Prionospio (Prionospio) caspersi Laubier, 1962

##### Notes

Type locality: Mediterranean (Venice Lagoon). Otherwise distributed in the North Atlantic and Pacific Ocean. Simboura (1996) notes that the Greek material differs slightly from the type material (Adriatic) and from material from Japan, suggesting that the Greek specimens may belong to an undescribed species.

#### Prionospio cirrifera

Wirén, 1883

Minuspio cirrifera (Wirén, 1883) | Prionospio (Minuspio) cirrifera Wirén, 1883 | *Prionospio cirrifera* Wirén, 1883

##### Notes

Questionable status. Reported frequently from Greece and other parts of the Mediterranean (Castelli et al. 2008, Zaâbi et al. 2012, Çınar et al. 2014, Mikac 2015) and considered cosmopolitan. However, Mackie (1984) re-examined European material identified as *Prionospio cirrifera* and found many specimens to actually belong to a species that could not be distinguished from *Prionospio multibranchiata* Berkeley, 1927, based on the available information at that time. Sigvaldadóttir (2002), in agreement with Mackie (1984), suggests that *Prionospio cirrifera* is a North Sea species and probably does not occur south of Portugal. Mackie suggested that Mediterranean specimens probably belong to other, endemic species (Mackie pers. comm. in Simboura 1996) and later, Dağlı and Çınar (2011) described *Prionospio maciolekae* from Mediterranean specimens. Other specimens of *Prionospio cirrifera* from Italy (Giangrande and Gambi 1982) were re-examined by Dağlı and Çınar (2011) and shown to belong to the alien species *Prionospio pulchra* Imajima, 1990. See also remarks under *Prionospio maciolekae*, *Prionospio multibranchiata* and *Prionospio pulchra*.

#### Prionospio decipiens

Söderström, 1920

##### Notes

Questionable status. In the Mediterranean only reported from Greece (Bogdanos and Nicolaidou 1985). Originally described from Portugal. The material was not available for re-examination, but the species' presence in Greece is considered questionable.

#### Prionospio dubia

Maciolek, 1985

##### Notes

Synonymised with *Prionospio steenstrupi* Malmgren, 1867 soon after the original description (Day 1963). Maciolek (1985) re-instated *Prionospio dubia* as a valid species and Sigvaldadóttir and Mackie (1993) assigned the description of *Prionospio steenstrupi* by Fauvel (1927) in part to *Prionospio dubia* (Sigvaldadóttir 2002). *Prionospio steenstrupi* seems to be restricted to boreal areas, whereas material from Greece was identified to belong to *Prionospio dubia* (Sigvaldadóttir pers. comm. in Simboura 1996).

#### Prionospio ehlersi

Fauvel, 1928

Prionospio (Prionospio) ehlersi Fauvel, 1928 | *Prionospio ehlersi* Fauvel, 1928

#### Prionospio ergeni

Dağlı & Çınar, 2009

##### Notes

New record for Greece. One specimen, Antikyra, Gulf of Corinth, 38°23'03.8''N, 22°39'14.5''E, 6.5 m depth, silty sand with biogenic detritus. Literature used for identification: Dağli and Çınar (2009). Type locality: Mediterranean (Turkey).

#### Prionospio fallax

Söderström, 1920

##### Notes

Frequently reported from Greece and other parts of the Mediterranean (Castelli et al. 2008, Ayari et al. 2009, Çınar et al. 2014, Mikac 2015). Simboura (1996), after examination of material from Greece and in communication with other experts (Sigvaldadóttir, Mackie), concludes that several specimens from Europe erroneously identified as *Prionospio malmgreni* Claparède, 1870 actually belong to *Prionospio fallax*. In the original description, *Prionospio malmgreni* was reported to have up to 9 pairs of branchiae, a character which was never found again in any specimen. The description of *Prionospio malmgreni by* Fauvel (1927) belongs to *Prionospio fallax*, leading to several misidentifications (Sigvaldadóttir and Mackie 1993). *Prionospio malmgreni* has been considered an indeterminable species (Maciolek 1985, Sigvaldadóttir and Mackie 1993) and it is suggested to assign previous Mediterranean records to *Prionospio fallax* (Çınar et al. 2014, Mikac 2015).

#### Prionospio maciolekae

Dağlı & Çınar, 2011

##### Notes

New record for Greece. Dağlı and Çınar (2011) described *Prionospio maciolekae* from material previously identified as *Prionospio multibranchiata* Berkeley, 1927 and question the presence of *Prionospio multibranchiata* in the Mediterranean. Re-examination of some of the Greek specimens of *Prionospio multibranchiata* revealed that they actually belong to *Prionospio maciolekae*. Type locality: Mediterranean (Turkey).

#### Prionospio multibranchiata

Berkeley, 1927

Minuspio multibranchiata (Berkeley, 1927) | *Prionospio multibranchiata* Berkeley, 1927 | Prionospio (Minuspio) multibranchiata Berkeley, 1927

##### Notes

Questionable status. Species complex. Reported frequently from Greece and other parts of the Mediterranean (Çınar 2005, Castelli et al. 2008, Mikac 2015). However, Mediterranean records probably belong to *Prionospio maciolekae* Dağlı and Çınar 2011 (see remarks there and under *Prionospio cirrifera* Wirén 1883). Otherwise distributed in the West Atlantic and East Pacific. Simboura (1996) describes two forms of Prionospio cf. multibranchiata, the one with longer gills which was later re-identified as *Prionospio pulchra* Imajima, 1990.

#### Prionospio pulchra

Imajima, 1990

##### Ecological interactions

###### Native status

Non-native (casual)

##### Notes

Reported from Greece by Pancucci-Papadopoulou et al. (2005). Originally from the Pacific Ocean, in the Mediterranean also known from Turkey (Çınar et al. 2014) and introduced to the East Atlantic (Moreira et al. 2000). Dağlı and Çınar (2011) suggest that the specimens of *Prionospio cirrifera* Wirén, 1883 collected from Italy by Giangrande and Gambi (1982) are identical to *Prionospio pulchra*. Mediterranean records of *Prionospio multibranchiata* Berkeley, 1927 and *Prionospio cirrifera* could comprise specimens of *Prionospio pulchra* (see Dağlı and Çınar 2011). See also remarks under *Prionospio cirrifera*.

#### Prionospio steenstrupi

Malmgren, 1867

Prionospio (Prionospio) steenstrupi Malmgren, 1867 | *Prionospio steenstrupi* Malmgren, 1867

##### Notes

Questionable status. Often confused with *Prionospio dubia* Maciolek, 1985 and several Greek specimens identified as *Prionospio steenstrupi* in fact belong to the former (see discussion there). In addition, Sigvaldadóttir and Mackie (1993) restrict its distribution to the coast of Iceland and consider all other reports doubtful. However, Dağli and Çınar (2009) confirm the presence of *Prionospio steenstrupi* in Turkey. Reported from the Atlantic, Pacific, Arctic and Indian Ocean.

#### Pseudopolydora antennata

(Claparède, 1869)

Polydora antennata Claparède, 1869 | Polydora (Carazzia) antennata Claparède, 1869 | *Pseudopolydora antennata* (Claparède, 1869)

##### Notes

Type locality: Mediterranean (Gulf of Naples).

#### Pseudopolydora paucibranchiata

(Okuda, 1937)

##### Ecological interactions

###### Native status

Non-native (casual)

##### Notes

Reported from Greece by Simboura et al. (2010), found again afterwards (HCMR, unpublished data). Originally described from Japan, in the Mediterranean also known from Turkey (Dağlı and Çınar 2008) and the Adriatic (Mikac 2015).

#### Pseudopolydora pulchra

(Carazzi, 1893)

##### Notes

Type locality: Mediterranean (Gulf of Naples).

#### Pygospio elegans

Claparède, 1863

##### Notes

Species complex. Radashevsky et al. (2016) provide molecular evidence that at least two sibling species are present in the Pacific Ocean. However, high genetic similarity was found between European and West Atlantic specimens. Currently, no evidence exists for the presence of more than one species in the Mediterranean.

#### Scolelepis bonnieri

(Mesnil, 1896)

Nerine bonnieri Mesnil, 1896 | *Scolelepis bonnieri* (Mesnil, 1896)

##### Notes

Reported from Greece by Harmelin (1969) and Antoniadou and Chintiroglou (2005). In the Mediterranean also reported from the central basin (Castelli et al. 2008), Cyprus (Çınar 2005) and Turkey (Çınar et al. 2014), otherwise known from the Atlantic coast of Europe.

#### Scolelepis cantabra

(Rioja, 1918)

Nerinides cantabra Rioja, 1918 | *Pseudomalacoceros cantabra* (Rioja, 1918) | *Scolelepis cantabra* (Rioja, 1918)

##### Notes

The new species *Scolelepis neglecta* Surugiu, 2016 was described after re-examination of Mediterranean material which was previously identified as *Scolelepis squamata* (O.F. Müller, 1806) and *Scolelepis cantabra* (Surugiu 2016). Reports of *Scolelepis cantabra* from Greece could therefore also comprise specimens of *Scolelepis neglecta*.

#### Scolelepis foliosa

(Audouin & Milne Edwards, 1833)

Nerine foliosa (Audouin & Milne Edwards, 1833) | *Scolelepis foliosa* (Audouin & Milne Edwards, 1833)

#### Scolelepis gilchristi

(Day, 1961)

Nerinides gilchristi Day, 1961

##### Notes

Questionable status. Reported from Greece by Nicolaidou and Pitta (1986). In the Mediterranean also known from Egypt (Ben-Eliahu 1972a) and Italy (Cantone and Fassari 1982). However, as the species is not included in subsequent reviews / checklists from Italy (Lardicci 1989, Castelli et al. 2008), it was probably considered a misidentification by the later authors. Originally described from South Africa.

#### Scolelepis squamata

(O.F. Müller, 1806)

Nerine cirratulus (Delle Chiaje, 1831) | *Scolelepis mesnili* (Bellan & Lagardère, 1971) | *Scolelepis squamata* (Müller, 1806)

##### Notes

Could comprise specimens of *Spiophanes neglecta* Surugiu, 2016 (see notes under *Scolelepis cantabra* (Rioja, 1918).

#### Scolelepis tridentata

(Southern, 1914)

Nerinides tridentata Southern, 1914 | *Pseudomalacoceros tridentata* (Southern, 1914) | *Scolelepis tridentata* (Southern, 1914)

#### Spio decoratus

Bobretzky, 1870

#### Spio filicornis

(Müller, 1776)

##### Notes

Questionable status. Frequently reported, but its presence in the Mediterranean has been questioned by Simboura (1996). In addition, Meißner et al. (2011) redescribed the species from the type locality (Iluilârssuk, Greenland) and concluded that traditionally used diagnostic characters are inappropriate. The same authors limit the species' distribution to Greenland and encourage the re-examination of material from other locations.

#### Spio multioculata

(Rioja, 1918)

#### Spiophanes bombyx

(Claparède, 1870)

##### Notes

Species complex. At least four morphologically similar species exist (Meißner and Blank 2009). All forms currently known have been formally described as different species in recent years. Specimens from the Turkish Aegean were studied by Meißner and Blank (2009) and found to belong to *Spiophanes bombyx*, thus it can be assumed that at least some of the Greek material belongs to *Spiophanes bombyx* sensu stricto. Type locality: Mediterranean (Gulf of Naples).

#### Spiophanes duplex

(Chamberlin, 1919)

Spiophanes soderstromi Hartman, 1953

##### Notes

Questionable status. Reported from Greece by NCMR (1989). Meißner (2005) restricts the distribution of the species to the Atlantic and Pacific coast of America and the Greek record is probably a misidentification.

#### Spiophanes kr﻿oyeri

Grube, 1860

Spiophanes kroyeri Grube, 1860| *Spiophanes kroyeri kroyeri* Grube, 1860

##### Notes

Questionable status. Frequently reported from Greece and other parts of the Mediterranean (Castelli et al. 2008, Çınar et al. 2014). However, Meißner (2005) concluded that so far only specimens from the North Atlantic can be assigned to *Spiophanes kroyeri*. Mikac (2015) could only confirm the presence of *Spiophanes afer* Meißner, 2005 from the Adriatic Sea, whereas *Spiophanes kroyeri* could not be found. *Spiophanes afer* is also known from the Turkish Aegean Sea and the Sea of Marmara (Dağli 2008, Dağlı et al. 2011).

#### Spiophanes reyssi

Laubier, 1964

Spiophanes kroyeri reyssi Laubier, 1964

##### Notes

Meißner (2005) raised *Spiophanes reyssi* to species level and described *Spiophanes mediterraneus* Meißner, 2005 from the Eastern Mediterranean, a species morphologically very close to *Spiophanes reyssi*. Thus, older records of *Spiophanes kroyeri reyssi* need to be treated with care as they may belong to either of the two species. *Spiophanes kroyeri reyssi* is reported from Greece and the Mediterranean from bathyal zones (Simboura and Nicolaidou 2001); *Spiophanes mediterraneus* is likewise described from depths of 300–700 m, a habitat which distinguishes these two species from congeneric species such as *Spiophanes kroyeri* or *Spiophanes afer*. Type locality: Mediterranean (off France).

#### Streblospio shrubsolii

(Buchanan, 1890)

### Sternaspidae Carus, 1863

#### Sternaspis scutata

Ranzani, 1817

##### Notes

Sendall and Salazar-Vallejo (2013) re-instated *Sternaspis thalassemoides* Otto, 1821 from the Mediterranean alongside *Sternaspis scutata*, mainly based on morphological features of the fan (truncate, entire and not expanding beyond the posterolateral margins in *Sternaspis thalassemoides*; notched and expanded beyond the posterolateral margins in *Sternaspis scutata*). Specimens recorded in the past as *Sternaspis scutata* could belong to either of the two species. Type locality: Mediterranean (originally Adriatic Sea, neotype from Izmir, Turkey).

### Syllidae Grube, 1850

#### Amblyosyllis formosa

(Claparède, 1863)

Amblyosyllis dorsigera Claparède, 1864 | *Amblyosyllis formosa* (Claparède, 1863) | *Pterosyllis formosa* Claparède, 1863

##### Notes

Three *Amblyosyllis* species have been recorded so far from the Mediterranean (*Amblyosyllis formosa*, *Amblyosyllis dorsigera* (Claparède, 1864) and *Amblyosyllis madeirensis* Langerhans, 1879); *Amblyosyllis dorsigera* is currently considered a synonym of *Amblyosyllis formosa*. However, the characters traditionally used to distinguish these three species (number and type of pharyngeal teeth) seem not to be consistent (Çınar and Ergen 2002) and the genus is in need of revision. At least two species of *Amblyosyllis* occur in Greece, as Arvanitidis (1994) reports both *Amblyosyllis formosa* and *Amblyosyllis madeirensis* in the same study; therefore both records are here retained as valid pending further analyses resolving their identity. Type locality: Mediterranean (Port-Vendres, France).

#### Amblyosyllis madeirensis

Langerhans, 1879

##### Notes

Reported from Greece by Arvanitidis (2000a). In the Mediterranean also known from Italy (Mikac 2015, Castelli et al. 2008) and Spain (San Martín 2003). See also notes for *Amblyosyllis formosa* (Claparède, 1863).

#### Anoplosyllis edentula

Claparède, 1868

Syllides edentulus (Claparède, 1868)

##### Notes

Questionable status. Reported from Greece by IOFR (1984). Type locality: Mediterranean (Gulf of Naples). While it is possible that the species occurs in Greece, it has so far only been reported in grey literature and has not been found in over three decades, therefore its presence in Greece is here considered questionable.

#### Autolytus rubrovittatus

Claparède, 1864

##### Notes

Questionable status. Reported from Greece by Karakassis (1991). Type locality: Mediterranean (Gulf of Naples). As type material has been lost and the original description is insufficient, the taxon is considered of unclear generic affiliation (Nygren 2004) and all subsequent records must be considered questionable.

#### Branchiosyllis cirropunctata

(Michel, 1909)

Syllis cirropunctata Michel, 1909 | Syllis (Typosyllis) cirropunctata Michel, 1909 | *Typosyllis cirropunctata* (Michel, 1909)

##### Notes

Treated as a synonym of *Branchiosyllis exilis* (Gravier, 1900) for many years (e.g. Licher 1999, San Martín 2003) and resurrected by San Martín et al. (2008), thus it may be underreported in Greece. Type locality: Mediterranean (Gulf of Naples).

#### ﻿B﻿ranchiosyllis exilis

(Gravier, 1900)

##### Notes

Species complex. The complex of pseudo-cryptic species around *Branchiosyllis exilis* is confused and in need of revision and it is likely that specimens around the world recorded under the nam*e Branchiosyllis exilis* belong to different species (San Martín et al. 2008). Specimens from the Mediterranean recorded as *Branchiosyllis exilis* could also belong to the native Mediterranean species *Branchiosyllis cirropunctata* (Michel, 1909), as the two species differ mainly in the shape of the shaft head of the falcigers and *Branchiosyllis cirropunctata* was considered a synonym of *Branchiosyllis exilis* until recently.

#### Brania arminii

(Langerhans, 1881)

Brania arminii (Langerhans, 1881) | *Brania oculata* (Hartmann-Schröder, 1960)

#### Brania pusilla

(Dujardin, 1851)

Brania pusilla (Dujardin, 1851) | *Grubea pusilla* (Dujardin, 1851)

#### Brevicirrosyllis weismanni

(Langerhans, 1879)

Pionosyllis weismanni Langerhans, 1879

#### Epigamia alexandri

(Malmgren, 1867)

Autolytus longeferiens Saint Joseph, 1887

##### Notes

Questionable status. Reported from Greece by Kisseleva (1983). In the Mediterranean also reported from Egypt (Fauvel 1937) and Italy (Castelli et al. 2008) but Nygren (2004) restricts it to the Arctic, the North-West Atlantic and the North Pacific.

#### Erinaceusyllis cryptica

(Ben-Eliahu, 1977)

Erinaceusyllis cryptica (Ben-Eliahu, 1977) | *Sphaerosyllis cryptica* Ben-Eliahu, 1977

##### Notes

Reported from Greece by Arvanitidis (1994), also found by Papageorgiou et al. (2006) (unpublished data). Widely distributed in the Mediterranean (Musco and Giangrande 2005).

#### Erinaceusyllis erinaceus

(Claparède, 1863)

Sphaerosyllis erinaceus Claparède, 1863

#### Eurysyllis tuberculata

Ehlers, 1864

##### Notes

Type locality: Mediterranean (Kvarner Bay, Adriatic).

#### Eusyllis assimilis

Marenzeller, 1875

##### Notes

Type locality: Mediterranean (Lošinj, Adriatic).

#### Eusyllis blomstrandi

Malmgren, 1867

#### Eusyllis lamelligera

Marion & Bobretzky, 1875

##### Notes

Type locality: Mediterranean (Marseille).

#### Exogone breviantennata

Hartmann-Schröder, 1959

Exogone ovalis Hartmann-Schröder, 1960

##### Ecological interactions

###### Native status

Non-native (casual)

##### Notes

Reported from Greece by Dando et al. (1995) in an ecological species list as *Exogone ovalis*. In the Mediterranean also known from Lebanon (Aguado and San Martín 2007) and Turkey (Çınar et al. 2014), otherwise it has a circumtropical distribution (e.g. San Martín 1991, Nogueira et al. 2004, Böggemann and Westheide 2004). The Eastern Mediterranean records make an occurrence of *Exogone breviantennata* in Greece likely, however, as it is very similar to the Mediterranean *Exogone verugera* (Claparède, 1868), (distinguished from the latter mainly by the presence of a dorsal cirrus on the second chaetiger (Aguado and San Martín 2007)), additional specimens are needed to confirm its presence.

#### Exogone dispar

(Webster, 1879)

Exogone (Exogone) dispar (Webster, 1879) | *Exogonedispar (Webster, 1879)*

#### Exogone lopezi

San Martín, Ceberio & Aguirrezabalaga, 1996

Exogone (Exogone) lopezi San Martín, Ceberio & Aguirrezabalaga, 1996

##### Notes

Deep sea species, in the Mediterranean only known from the bathyal zone of the North Aegean (Simboura and Zenetos 2005). In the Mediterranean also reported from deep waters off Sardinia (Busoni 2013), otherwise known from the Atlantic coasts of the Iberian Peninsula.

#### Exogone naidina

Ørsted, 1845

Exogone (Exogone) naidina Ørsted, 1845 | *Exogone gemmifera* Pagenstecher, 1862 | *Exogone naidina* Ørsted, 1845

#### Exogone rostrata

Naville, 1933

Exogone (Exogone) rostrata Naville, 1933 | *Exogone rostrata* Naville, 1933

##### Notes

Type locality: Mediterranean (Banyuls-sur-Mer, France).

#### Exogone sorbei

San Martín, Ceberio & Aguirrezabalaga, 1996

Exogone sorbei San Martín, Ceberio & Aguirrezabalaga, 1996 | Exogone (Exogone) sorbei San Martín, Ceberio & Aguirrezabalaga, 1996

##### Notes

Deep sea species, in the Mediterranean only known from the bathyal zone of the North Aegean (Simboura and Zenetos 2005). Otherwise known from the Atlantic coasts of the Iberian Peninsula.

#### Exogone torulosa

(Claparède, 1864)

Spermosyllis torulosa Claparède, 1864

##### Notes

Questionable status. A poorly known species which is frequently reported in ecological lists, but no recent descriptions exist (the newest being by Fauvel (1923), who reproduces description and illustrations by Claparède (1864) and Soulier (1904)). The species is not included in the treatise of the family by San Martín (2003). Type locality: Mediterranean (Port-Vendres, France).

#### Exogone verugera

(Claparède, 1868)

Exogone (Exogone) verugera (Claparède, 1868) | *Exogone verugera* (Claparède, 1868)

##### Notes

Type locality: Mediterranean (Gulf of Naples).

#### Haplosyllis spongicola

(Grube, 1855)

Haplosyllis hamata (Claparède, 1868) | *Haplosyllis spongicola* (Grube, 1855) | *Syllis spongicola* Grube, 1855 | Syllis (Haplosyllis) spongicola Grube, 1855

##### Notes

Species complex. The taxonomy of the species is confused, with a long list of synonyms. Originally described from the Northern Adriatic but reported from worldwide locations (Martín et al. 2003), with three different morphotypes in the Western Mediterranean. Currently, three well-described species of *Haplosyllis* are known the Mediterranean: *Haplosyllis spongicola, Haplosyllis chamaeleon* Laubier, 1960 and *Haplosyllis granulosa* (Lattig, San Martín & Martin, 2007). The description of *Haplosyllis spongicola* by San Martín (2003) is referred in part to *Haplosyllis granulosa* (Lattig et al. 2007). In addition, a number of *Haplosyllis* species have been described from the Mediterranean and the Black Sea which are currently regarded as incertae sedis due to lost type material and insufficient descriptions (Lattig and Martín 2009). Eastern Mediterranean records should also be examined for possible occurrences of the Red Sea species *Haplosyllis djiboutiensis* Gravier, 1900.

#### Myrianida brachycephala

(Marenzeller, 1874)

Autolytus brachycephalus (Marenzeller, 1874)

##### Notes

Type locality: Mediterranean (Adriatic).

#### Myrianida convoluta

(Cognetti, 1953)

Autolytus convolutus Cognetti, 1953 | *Myrianida convoluta* (Cognetti, 1953)

##### Notes

Type locality: Mediterranean (Gulf of Naples).

#### Myrianida edwarsi

(Saint Joseph, 1887)

Autolytus edwarsi Saint Joseph, 1887 | *Myrianida edwarsi* (Saint Joseph, 1887)

#### Myrianida inermis

(Saint Joseph, 1887)

##### Notes

Questionable status. Reported from Greece by Faulwetter et al. (2011a) based on a single specimen. In the Mediterranean also known from Italy (Somaschini 1988). Nygren (2004) restricts its distribution to the North-East Atlantic and North-East Pacific. Mediterranean specimens could be misidentifications in which the trepan was overlooked. In addition, the Greek specimen was identified using the key and description by San Martín (2003) which, according to Nygren (2004), corresponds only in part to *Myrianida inermis*.

#### Myrianida pinnigera

(Montagu, 1808)

##### Notes

Reported from Greece by Arvanitidis (2000a) based on a single specimen. Widely distributed in the Mediterranean (Nygren 2004).

#### Myrianida prolifera

(O.F. Müller, 1788)

Autolytus prolifer (O.F. Müller, 1788) | *Myrianida prolifera* (O.F. Müller, 1788)

##### Notes

The name has been applied to different taxa in the past, including stolons (Gidholm 1967). Nygren (2004) restricts its distribution to the North Atlantic and cautions that preserved specimens are easily confused with other taxa.

#### Myrianida quindecimdentata

(Langerhans, 1884)

##### Notes

Reported from Greece by Faulwetter et al. (2011a) and Keklikoglou et al. (2013). Widely distributed in the Mediterranean (Musco and Giangrande 2005).

#### Myrianida rodosensis

Çınar, 2015

##### Notes

Originally described from Rhodes (36°20.13′N, 28°12.52′E; 1 m depth), no other records from Greece.

#### Myrianida rubropunctata

(Grube, 1860)

Autolytus rubropunctatus (Grube, 1860)

##### Notes

Type locality: Mediterranean (Kraljevica, Croatia).

#### Neopetitia amphophthalma

(Siewing, 1956)

Petitia amphophthalma Siewing, 1956

##### Notes

Species complex. Reported from Greece by von Soosten et al. (1998) in the framework of a molecular analysis. Greek specimens differ genetically from those from the type locality (Arcachon Bay), but the authors do not consider the differences significant enough to separate different species (von Soosten et al. 1998, Westheide and Hass-Cordes 2001).

#### Nudisyllis divaricata

(Keferstein, 1862)

Nudisyllis divaricata (Keferstein, 1862) | *Pionosyllis divaricata* (Keferstein, 1862)

#### Nudisyllis pulligera

(Krohn, 1852)

Pionosyllis pulligera (Krohn, 1852) | *Pionosyllis serrata* Southern, 1914

##### Notes

Type locality: Mediterranean.

#### Odontosyllis ctenostoma

Claparède, 1868

##### Notes

Type locality: Mediterranean (Gulf of Naples).

#### Odontosyllis dugesiana

Claparède, 1864

##### Notes

Reported from Greece by Fassari (1982). Type locality: Mediterranean (Port-Vendres, France).

#### Odontosyllis fulgurans

(Audouin & Milne Edwards, 1833)

##### Notes

Type locality: Mediterranean.

#### Odontosyllis gibba

Claparède, 1863

#### Opisthodonta longocirrata

(Saint-Joseph, 1887)

Pionosyllis longocirrata Saint Joseph, 1887

##### Notes

Questionable status. Reported from Greece by Xenopoulou (1987). Widely distributed throughout the Mediterranean (Musco and Giangrande 2005). While it is possible that the species occurs in Greece, it has so far only been reported in grey literature and has not been found in over three decades, therefore its presence in Greece is here considered questionable.

#### Opisthodonta morena

Langerhans, 1879

##### Notes

New record for Greece. Six specimens, Othoni, off Corfu, 39°47'50.3"N, 19°28'13.6"E, 29 m depth, coarse biogenic sand and *Posidonia oceanica* fragments, collected in the framework of the COCONET project, Literature used for identification: San Martín (2003). Widely distributed throughout the Mediterranean (Musco and Giangrande 2005).

#### Opisthosyllis brunnea

Langerhans, 1879

##### Notes

Reported from Greece by Faulwetter et al. (2011a) and Keklikoglou et al. (2013). Widely distributed in the Mediterranean (Musco and Giangrande 2005). Considered cosmopolitan, but Paresque et al. (2016) found morphological differences between specimens from different localities and raise the possibility of *Opisthosyllis brunnea* constituting a species complex.

#### Paraehlersia dionisi

(Núñez & San Martín, 1991)

Pionosyllis dionisi Núñez & San Martín, 1991

##### Notes

Reported from Greece by NCMR (1995) and Simboura (1996), identification confirmed by G. San Martín (pers. comm. in Simboura 1996). In the Mediterranean also known from the Western Mediterranean (Musco and Giangrande 2005), Turkey (Çınar et al. 2014) and the Adriatic (Mikac 2015), otherwise known from the Canary Islands and Antarctica (San Martín et al. 2009).

#### Paraehlersia ferrugina

(Langerhans, 1881)

Ehlersia ferrugina Langerhans, 1881 | *Langerhansia ferrugina* (Langerhans, 1881) | *Paraehlersia ferrugina* (Langerhans, 1881) | *Syllis ferrugina* (Langerhans, 1881)

#### Parapionosyllis brevicirra

Day, 1954

#### Parapionosyllis cabezali

Parapar, San Martín & Moreira, 2000

#### Parapionosyllis elegans

(Pierantoni, 1903)

##### Notes

Questionable status. Reported from Greece by IOFR (1984). Type locality: Mediterranean (Gulf of Naples). While it is possible that the species occurs in Greece, it has so far only been reported in grey literature and has not been found in over three decades, therefore its presence in Greece is here considered questionable.

#### Parapionosyllis minuta

(Pierantoni, 1903)

##### Notes

Type locality: Mediterranean (Gulf of Naples).

#### Parexogone campoyi

(San Martín, Ceberio & Aguirrezabalaga, 1996)

Exogone (Parexogone) campoyi San Martín, Ceberio & Aguirrezabalaga, 1996

##### Notes

Deep sea species, reported from Greece by Simboura and Zenetos (2005) from the bathyal zone of the North Aegean. In the Mediterranean also reported from deep waters in the Ionian Sea (Langeneck et al. 2017b), otherwise known from the Atlantic coasts of the Iberian Peninsula.

#### Parexogone gambiae

Lanera, Sordino & San Martín, 1994

Exogone gambiae Lanera, Sordino & San Martín, 1994 | Exogone (Parexogone) gambiae Lanera, Sordino & San Martín, 1994

##### Notes

Type locality: Mediterranean (Ponza, Tyrrhenian Sea).

#### Parexogone hebes

(Webster & Benedict, 1884)

Exogone hebes (Webster & Benedict, 1884) | Exogone (Parexogone) hebes (Webster & Benedict, 1884) | *Parexogone hebes* (Webster & Benedict, 1884)

#### P﻿arexogone ﻿merid﻿ionalis﻿﻿

Cognetti, 1955

Exogone parahomoseta mediterranea San Martín, 1984

##### Notes

New record for Greece. Three specimens, Elafonisi, Crete, 35°16'20.7"N, 23°32'15.9"E, 1 m depth, fine sand, collected in the framework of the MEDCORE project (unpublished data from Papageorgiou et al. 2006). Literature used for identification: Cognetti (1955), San Martín (2003). Type locality: Mediterranean (Gulf of Naples).

#### Parexogone wolfi

San Martín, 1991

Exogone (Parexogone) wolfi San Martín, 1991

##### Notes

Reported from Greece from the bathyal zone of the North Aegean (Simboura and Zenetos 2005). In the Mediterranean also known from Cyprus (Çınar et al. 2003), otherwise distributed on both sides of the Atlantic (San Martín 2003).

#### Perkinsyllis anophthalma

(Capaccioni & San Martín, 1990)

Pionosyllis anophthalma Capaccioni & San Martín, 1990

##### Notes

Reported from Greece by Simboura et al. (2007). Type locality: Mediterranean (Catalonia, Spain).

#### Plakosyllis brevipes

Hartmann-Schröder, 1956

Plakosyllis brevipes Hartmann-Schröder, 1956 | *Plakosyllis quadrioculata* Perkins, 1981

##### Notes

Type locality: Mediterranean.

#### Proceraea aurantiaca

Claparède, 1868

Autolytus aurantiacus (Claparède, 1868) | *Proceraea aurantiaca* Claparède, 1868

##### Notes

Has often been confused with similar species, especially with *Proceraea paraurantiaca* Nygren, 2004 and older records should be treated with caution (Nygren 2004). Type locality: Mediterranean (Gulf of Naples).

#### Proceraea picta

Ehlers, 1864

Autolytus pictus (Ehlers, 1864) | *Proceraea picta* Ehlers, 1864

##### Notes

As preserved specimens of *Proceraea picta* are often impossible to distinguish from other species, literature records need to be treated with care (Nygren 2004). Type locality: Mediterranean (Croatia).

#### Procerastea nematodes

Langerhans, 1884

Procerastea nematodes Langerhans, 1884 | *Procerastea perrieri* Gravier, 1900

##### Notes

Reported from Greece as *Procerastea perrieri* by Nicolaidou et al. (1986) and Simboura et al. (1995b), subsequently listed as *Procerastea nematodes* by Arvanitidis (2000a) and Simboura and Nicolaidou (2001). In the Mediterranean also reported from Italy (Castelli et al. 2008), otherwise distributed along the Atlantic coasts of Europe. Nygren (2004) restricts the species' distribution to the North-East Atlantic and the North-East Pacific.

#### Prosphaerosyllis brevicirra

(Hartmann-Schröder, 1960)

Sphaerosyllis brevicirra Hartmann-Schröder, 1960

##### Notes

Questionable status. Records of *Sphaerosyllis brevicirra* from the Western Mediterranean by Alós (1989) and from the Aegean Sea (Simboura 1996, Çınar 1999) belong to an undescribed *Prosphaerosyllis* species (San Martín 2003). Faulwetter et al. (2011a) examined the holotype of *Prosphaerosyllis brevicirra* from the Red Sea and found that the species bears a conspicuous papilla on each dorsal cirrus. All descriptions and illustrations of *Prosphaerosyllis brevicirra* from the Mediterranean lack these papillae. In addition, these specimen descriptions differ from *Prosphaerosyllis brevicirra* by the absence of dorsal cirri on chaetiger 2 (reported as present by Alós (1989) but in fact absent (San Martín 2003) and by thicker aciculae.

#### Prosphaerosyllis campoyi

(San Martín, Acero, Contonente & Gomez, 1982)

Prosphaerosyllis campoyi (San Martín, Acero, Contonente & Gomez, 1982) | *Sphaerosyllis campoyi* San Martín, Acero, Contonente & Gomez, 1982

##### Notes

Reported from Greece by Faulwetter et al. (2011a), also found by Papageorgiou et al. (2006) (unpublished data). Type locality: Mediterranean (Andalusia, Spain).

#### Prosphaerosyllis tetralix

(Eliason, 1920)

Sphaerosyllis tetralix Eliason, 1920

##### Notes

Questionable status. Reported from Greece by Dando et al. (1995); the record by Zenetos et al. (1997) probably belongs to a different species. Records from throughout the Mediterranean exist but at least some of them belong to other species (Faulwetter et al. 2011a) and the species is probably restricted to the colder waters of the North Sea and North-East Atlantic (Olivier et al. 2012). During the last years, several new *Prosphaerosyllis* species have been described from Europe and the Mediterranean and Mediterranean species of the genus need to be revised. The Greek record is from near hydrothermal vents where temperatures are probably unfavourable for the species and must be considered questionable.

#### Prosphaerosyllis xarifae

(Hartmann-Schröder, 1960)

##### Notes

Reported from Greece by Faulwetter et al. (2011a). Widely distributed in the Mediterranean (Musco and Giangrande 2005).

#### Pseudosyllis brevipennis

Grube, 1863

Pseudosyllis brevipennis Grube, 1863 | *Syllis brevipennis* (Grube, 1863) | *Trypanosyllis coeliaca* Claparède, 1868 | *Typosyllis brevipennis* (Grube, 1863)

##### Notes

*Pseudosyllis brevipennis* was considered a synonym of *Trypanosyllis coeliaca* Claparède, 1868 by San Martín (2003), although *Pseudosyllis brevipennis*, being the older name, should have priority over *Trypanosyllis coeliaca*. San Martín (2003), however, considered the former a nomen dubium and chose the latter as the preferred name (San Martín 2003, Álvarez-Campos et al. 2017). A molecular analysis using multiple loci (Álvarez-Campos et al. 2017) reveals that *Trypanosyllis coeliaca* differs distinctly from other *Trypanosyllis* species and is much closer related to *Xenosyllis*, *Eurysyllis* and *Plakosyllis*. These authors re-instate *Pseudosyllis brevipennis*, designate a neotype from Rovigno (Croatia) and place *Trypanosyllis coeliaca* into synonymy with *Pseudosyllis brevipennis*.

#### Salvatoria alvaradoi

(San Martín, 1984)

##### Notes

Reported from Greece by Faulwetter et al. (2011a). Type locality: Mediterranean (Balearic Islands).

#### Salvatoria clavata

(Claparède, 1863)

Brania clavata (Claparède, 1863) | *Grubea clavata* (Claparède, 1863) | *Grubeosyllis clavata* (Claparède, 1863) | *Salvatoria clavata* (Claparède, 1863)

##### Notes

Type locality: Mediterranean (Gulf of Naples).

#### Salvatoria euritmica

(Sardá, 1984)

##### Notes

Reported from Greece by Faulwetter et al. (2011a). Widely distributed in the Mediterranean Sea (Musco and Giangrande 2005), originally described from Gibraltar.

#### Salvatoria limbata

(Claparède, 1868)

Grubeosyllis limbata (Claparède, 1868) | *Pseudobrania limbata* (Claparède, 1868) | *Salvatoria limbata* (Claparède, 1868)

##### Notes

Type locality: Mediterranean (Gulf of Naples).

#### Salvatoria neapolitana

(Goodrich, 1930)

##### Notes

Reported from Greece by Faulwetter et al. (2011a) and Keklikoglou et al. (2013). Type locality: Mediterranean (Gulf of Naples).

#### Salvatoria tenuicirrata

(Claparède, 1864)

Brania tenuicirrata (Claparède, 1864)

##### Notes

Questionable status. A poorly known species which is frequently reported in ecological lists, but no recent descriptions exist (the newest being Fauvel (1923), who reproduces description and illustrations by Claparède (1864)). Fauvel considers the species very close to *Salvatoria clavata* (Claparède, 1863) from which it differs in "minor and variable characters". The species is not included in the treatise of the family by San Martín (2003). Type locality: Mediterranean (Port-Vendres, France).

#### Salvatoria vieitezi

(San Martín, 1984)

##### Notes

Reported from Greece by Faulwetter et al. (2011a). Type locality: Mediterranean (Balearic Islands).

#### Salvatoria yraidae

(San Martín, 1984)

##### Notes

Reported from Greece by Faulwetter et al. (2011a). Type locality: Mediterranean (Balearic Islands).

#### Sphaerosyllis austriaca

Banse, 1959

##### Notes

Reported from Greece by Faulwetter et al. (2011a). Type locality: Western Mediterranean.

#### Sphaerosyllis boeroi

Musco, Çınar & Giangrande, 2005

##### Notes

Reported from Greece by Faulwetter et al. (2011a) based on specimens previously identified as *Sphaerosyllis hystrix*. Type locality: Mediterranean (Porto Cesareo, Ionian Sea).

#### Sphaerosyllis bulbosa

Southern, 1914

#### Sphaerosyllis capensis

Day, 1953

##### Notes

Questionable status. Reported from Greece by Arvanitidis (2000a), in the Mediterranean also reported from Spain (San Martín et al. 1981b), otherwise distributed along the coast of South Africa and in the Indian Ocean. The specimens may have been confused with other Mediterranean species. *Sphaerosyllis capensis* is distinguished from many congeners by having all antennae in line. However, the position of the antennae in preserved material may be misleading if fixation has caused muscle contraction (Riser 1991).

#### Sphaerosyllis claparedei

Ehlers, 1864

##### Notes

Questionable status. *Sphaerosyllis claparedei* is a poorly known species. Ehler's original description is detailed overall, but not clear on all characters that are used nowadays to distinguish species (e.g. papillation pattern, shape of chaetae, parapodial glands etc.). The species is not included in the treatise of the family by San Martín (2003). A character which allegedly distinguishes *Sphaerosyllis claparedei* from other species of the genus is the presence of a dorsal cirrus on the second chaetiger. However, other species were also originally described with this feature (e.g. *Sphaerosyllis hystrix* Claparède, 1863) although it is in fact absent (see also remarks under *Sphaerosyllis ovigera* Langerhans, 1879). Ehlers (1864) also describes the dorsal cirri as having a “knob-like shape” at their tip, at least in the anterior chaetigers. This could be an indication for retractile cirri, as seen in *Prosphaerosyllis*. As no recent re-descriptions exist which take into account characters used nowadays to distinguish species, the status of the species remains unclear and specimens should be revised. Type locality: Mediterranean (Porto di Lazaretto, Adriatic).

#### Sphaerosyllis glandulata

Perkins, 1981

##### Notes

Reported from Greece by Faulwetter et al. (2011a). In the Mediterranean also known from Spain (Somaschini and San Martín 1994), Italy (Castelli et al. 2008) and Turkey (Çınar et al. 2014), otherwise distributed on both sides of the Atlantic (San Martín 2003).

#### Sphaerosyllis gravinae

Somaschini & San Martín, 1994

##### Notes

New record for Greece. Two specimens, Elafonisi, Crete, 35°16'20.7"N, 23°32'15.9"E, 1 m depth, fine sand, collected in the framework of the MEDCORE project (unpublished data from Papageorgiou et al. 2006). Literature used for identification: Somaschini and San Martín (1994). Type locality: Mediterranean (Tyrrhenian Sea).

#### Sphaerosyllis hystrix

Claparède, 1863

##### Notes

The name *Sphaerosyllis hystrix* has been applied to a number of specimens worldwide which likely belong to other species. Several specimens from Greece previously identified as *Sphaerosyllis hystrix* were assigned to *Sphaerosyllis boeroi* Musco, Çınar & Giangrande, 2005 after re-examination (Faulwetter et al. 2011b). It is likely that other specimens recorded under the name *Sphaerosyllis hystrix* from Greece actually belong to different species. Type locality: Mediterranean (Gulf of Naples).

#### Sphaerosyllis magnidentata

Perkins, 1981

##### Notes

Questionable status. Reported from Greece by Koulouri et al. (2015), in the Mediterranean also known from Spain (Tena et al. 2000), otherwise distributed in the Caribbean. The Mediterranean occurrence record leads to the inclusion of the species in the key of Mediterranean *Sphaerosyllis* by Faulwetter et al. (2011b), but the rarity of records indicates that it may in fact not be present in the region. A re-examination of the specimens is required to verify its presence in Greece and the Mediterranean.

#### Sphaerosyllis ovigera

Langerhans, 1879

##### Notes

Questionable status. A poorly known species which is frequently reported in ecological lists, but no recent descriptions exist (the newest being Fauvel (1923), who reproduces description and illustrations by Langerhans (1879)). The species is not included in the treatise of the family by San Martín (2003). A character which allegedly distinguishes *Sphaerosyllis ovigera* from other species of the genus is the presence of a dorsal cirrus on the second chaetiger. However, other species were originally also described with this feature (e.g. *Sphaerosyllis hystrix*) although it is in fact absent. Non-type material of Langerhans in the Natural History Museum of Vienna (Inv. No. 2549, Acq. No. 3296, locality Madeira, coll. year 1884) was examined by S. Faulwetter; the dorsal cirrus on chaetiger 2 is absent. The status of the species remains unclear and specimens should be revised.

#### Sphaerosyllis pirifera

Claparède, 1868

##### Notes

Type locality: Mediterranean (Gulf of Naples).

#### Sphaerosyllis riseri

Perkins, 1981

##### Notes

New record for Greece. Two specimens, Elafonisi, Crete, 35°16'20.7"N, 23°32'15.9"E, 1 m depth, fine sand, collected in the framework of the MEDCORE project (unpublished data from Papageorgiou et al. 2006). Literature used for identification: Perkins (1981). No other Mediterranean records, otherwise distributed along the Atlantic coast of North America.

#### Sphaerosyllis taylori

Perkins, 1981

#### Sphaerosyllis thomasi

San Martín, 1984

##### Notes

Reported from Greece by Arvanitidis (2000a). Type locality: Mediterranean (Balearic Islands).

#### Streptodonta pterochaeta

(Southern, 1914)

Opisthodonta pterochaeta Southern, 1914

##### Notes

Reported from Greece by Simboura (1996) and Zenetos et al. (1997) (based on the same specimens), identification confirmed by G. San Martín (pers. comm. in Simboura 1996). In the Mediterranean also reported from Spain (Parapar et al. 1993b) and Egypt (Abd-Elnaby 2010), otherwise distributed along the Atlantic coasts of Europe.

#### Streptosyllis bidentata

Southern, 1914

##### Notes

Questionable status. Reported from Greece by Xenopoulou (1987), in the Mediterranean also known from Spain (San Martín 2003) and Tunisia (Zaâbi et al. 2012). Present in the adjacent Sea of Marmara (Çınar et al. 2014). Otherwise distributed in the North East Atlantic.

#### Streptosyllis nunezi

Faulwetter, Vasileiadou, Papageorgiou & Arvanitidis, 2008

##### Notes

Originally described from Crete (Pachia Ammos; 35º06'43''N 25º48'34''E; 1–5 m depth), no other records from Greece.

#### Streptosyllis websteri

Southern, 1914

#### Syllides bansei

Perkins, 1981

##### Notes

Reported from Greece by Simboura (1996), identification confirmed by G. San Martín (pers. comm. in Simboura 1996). Also found by Papageorgiou et al. (2006) (unpublished data). In the Mediterranean also reported from Spain (Alós 1989), Italy (Castelli et al. 2008) and Turkey (Çınar et al. 2014), otherwise distributed on both sides of the Atlantic (San Martín 2003).

#### Syllides benedicti

Banse, 1971

##### Notes

In the Mediterranean only reported from Greece (Dando et al. 1995; also found by M. Simboura in the North Aegean (HCMR, unpublished data)). Commonly distributed in the North Atlantic.

#### Syllides convolutus

Webster & Benedict, 1884

##### Notes

New record for Greece. One specimen, Elafonisi, Crete, 35°16'20.7"N, 23°32'15.9"E, 1 m depth, fine sand, collected in the framework of the MEDCORE project (unpublished data from Papageorgiou et al. 2006). Literature used for identification: San Martín (1984), San Martín (2003). In the Mediterranean also known from the western Basin (Musco and Giangrande 2005), otherwise distributed along the Atlantic coast of North America.

#### Syllides edentatus

Westheide, 1974

##### Notes

Reported from Greece by Faulwetter et al. (2011a). In the Mediterranean also known from Spain (San Martín 2003), Italy (Cosentino 2011) and Turkey (Çınar et al. 2014). Cited from worldwide locations.

#### Syllides fulvus

(Marion & Bobretzky, 1875)

##### Notes

Type locality: Mediterranean (Adriatic).

#### Syllides japonicus

Imajima, 1966

##### Notes

Reported from Greece by Faulwetter et al. (2011a). In the Mediterranean also reported from Spain (San Martín 2003), Turkey (Çınar et al. 2014) and the Adriatic (Mikac 2015). Cited from disjunct worldwide locations (San Martín 2003).

#### Syllides longocirratus

(Ørsted, 1845)

##### Notes

Questionable status. The species described as *Syllides longocirrata* by Fauvel (1923) actually belongs to *Syllides fulvus* (Marion & Bobretzky, 1875) (Parapar et al. 1992, Çınar and Ergen 2003), thus any specimens identified using Fauvel's key probably belong to *Syllides fulvus*. Campoy (1982) considers Mediterranean specimens from soft substrates to belong to *Syllides fulvus* and specimens from hard substrates to *Syllides edentatus*. Records from Greece are all from soft substrates, thus probably belong to *Syllides fulvus*.

#### Syllis alternata

Moore, 1908

##### Notes

Species complex. Consists of at least five putative cryptic species in the North American Pacific coasts, near the type locality (Carr et al. 2011). Specimens from Europe could belong to either of these cryptic species or to a different one. Reported from Greece by Faulwetter et al. (2011a) and Maidanou et al. (2017), widely distributed in the Mediterranean (Musco and Giangrande 2005).

#### Syllis amica

Quatrefages, 1866

#### Syllis armillaris

(O.F. Müller, 1776)

Syllis armillaris (O.F. Müller, 1776) | Syllis (Typosyllis) armillaris (O.F. Müller, 1776) | *Typosyllis armillaris* (O.F. Müller, 1776)

##### Notes

Considered cosmopolitan, but populations from different geographic locations show morphological differences and the species could actually constitute a complex of pseudo-cryptic species (López et al. 2001).

#### Syllis beneliahuae

(Campoy & Alquézar, 1982)

##### Notes

Type locality: Mediterranean (Spain).

#### Syllis columbretensis

(Campoy, 1982)

##### Notes

Licher (1999) considers *Syllis columbretensis* a synonym of *Syllis variegata* Grube, 1860. San Martín (2003) lists it as a valid species. Specimens from Greece reported by Faulwetter et al. (2011a) and Keklikoglou et al. (2013) were identified using the description and key by San Martín (2003), but those identified by Arvanitidis (2000a) could belong to either *Syllis corallicola* Verrill, 1900 or *Syllis columbretensis*, as he used both the description by Campoy (1982) and the key by San Martín (1984) to identify the specimens. *Syllis columbretensis* sensu San Martin was, however re-identified as *Syllis corallicola* (Licher 1999, San Martín 2003). Type locality: Mediterranean (Columbretes Islands, Spain).

#### Syllis compacta

Gravier, 1900

##### Notes

Reported from Greece by Faulwetter et al. (2011a) and Keklikoglou et al. (2013). Widely distributed in the Mediterranean (Musco and Giangrande 2005). Licher (1999) considers *Syllis compacta* a synonym of *Syllis variegata* Grube, 1860, San Martín (2003) lists it as a valid species. Specimens from Greece were identified using the description and key by San Martín (2003).

#### Syllis corallicola

Verrill, 1900

##### Notes

Reported from Greece by Faulwetter et al. (2011a) and Keklikoglou et al. (2013). Widely distributed in the Mediterranean (Musco and Giangrande 2005).

#### Syllis cornuta

Rathke, 1843

Ehlersia cornuta (Rathke, 1843) | *Langerhansia cornuta* (Rathke, 1843) | *Syllis cornuta* Rathke, 1843

##### Notes

Questionable status. San Martin and Lopez (2000) suggest that all European records of *Syllis cornuta* should be assigned to *Syllis parapari* San Martín & Lopez, 2000. *Mikac and Musco (2010)* consider it possible that they could belong to any of the Mediterranean *Syllis* with pseudospinigerous chaetae (*Syllis beneliahuae* (Campoy & Alquézar, 1982), *Syllis rosea* Langerhans, 1879, *Syllis garciai* (Campoy, 1982) or *Syllis parapari)*. Licher (1999) examined specimens identified as *Syllis cornuta* from Greece and found that none of these specimens actually belonged to *Syllis cornuta*. However, all Greek specimens would need to be re-examined to determine their true identity.

#### Syllis cruzi

Núñez & San Martín, 1991

##### Notes

Reported from Greece by Faulwetter et al. (2011a). In the Mediterranean also known from Spain (San Martín 2003), Cyprus (Çınar and Ergen 2003), Turkey (Çınar et al. 2014) and the Adriatic (Mikac 2015); otherwise known from the Canary Islands.

#### Syllis ferrani

Alós & San Martín, 1987

##### Notes

Rarely reported from Greece, identifications of specimens by Simboura (1996) were confirmed by G. San Martín (pers. comm.). Type locality: Mediterranean (Cap Creus, Spain).

#### Syllis garciai

(Campoy, 1982)

##### Notes

Type locality: Mediterranean (Balearic Islands).

#### Syllis gerlachi

(Hartmann-Schröder, 1960)

Syllis gerlachi (Hartmann-Schröder, 1960) | *Syllis truncata cryptica* Ben-Eliahu, 1977 | Syllis (Typosyllis) truncata cryptica Ben-Eliahu, 1977

##### Notes

Licher (1999) synonymised *Syllis truncata cryptica* with *Syllis gerlachi*. San Martín et al. (2017) examined the holotype of *Syllis gerlachi* and found it to differ from Mediterranean specimens of *Syllis truncata cryptica* in the shape of the posterior aciculae and other chaetal features (no exact details given). They consider the synonymy incorrect, but as they do not formally re-instate *Syllis truncata cryptica*, we here report records of both species under the name *Syllis gerlachi* until further evidence is provided.

#### Syllis gerundensis

(Alós & Campoy, 1981)

##### Notes

Reported from Greece by Faulwetter et al. (2011a) and Maidanou et al. (2017). Type locality: Mediterranean (Gerona, Spain).

#### Syllis gracilis

Grube, 1840

##### Notes

Species complex. Originally described from the Gulf of Naples. Maltagliati et al. (2000) found two cryptic species of *Syllis gracilis* in Sardinia and western Italy, Álvarez-Campos et al. (2016) recover three cryptic species in the Mediterranean: one from Sicily and two from the North-Western Mediterranean. No material from the Eastern Mediterranean was studied, and specimens from Greece could belong to any of these or a different species.

#### Syllis hyalina

Grube, 1863

Syllis hyalina Grube, 1863 | *Typosyllis hyalina* (Grube, 1863) | Typosyllis (Syllis) hyalina (Grube, 1863)

##### Notes

Type locality: Mediterranean (Croatia).

#### Syllis jorgei

San Martín & López, 2000

##### Notes

Reported from Greece by Faulwetter et al. (2011a) under this name, previously reported as *Syllis lutea* (Hartmann-Schröder, 1960) by Arvanitidis (1994) (see also Table 1). Type locality: Mediterranean (Columbretes Islands, Spain).

#### Syllis katzmanni

Arvanitidis, 2017, nomen novum

Langerhansia caeca Katzmann, 1973 | *Syllis caeca* (Katzmann, 1973) | *Syllis katzmanni* (Katzmann, 1973) [as a temporary name]

##### Notes

Reported from Greece by Arvanitidis (1994). The species was originally described as *Langerhansia caeca* by Katzmann (1973) from the Adriatic. San Martín (1984) synonymised *Langerhansia* with *Syllis*, thus *Langerhansia caeca* Katzmann, 1973 became *Syllis caeca* (Katzmann, 1973), but the name was preoccupied by *Syllis caeca* Monro 1933. Arvanitidis (1994) proposed the replacement name *Syllis katzmanni* in his PhD thesis, which was however never validly published under the International Code of Zoological Nomenclature (ICZN). Licher (1999) considered *Syllis* and *Typosyllis* two different genera and placed *Syllis caeca* (Katzmann, 1973) back into *Typosyllis*, removing the need for a replacement name and thus synonymising the temporary name *Syllis katzmanni* (Katzmann, 1973) with *Typosyllis caeca* (Katzmann, 1973). He also considered *Syllis caeca* Monro, 1933 a nomen dubium (which does not affect the availability of the name). San Martín (2003) re-synonymised *Typosyllis* with *Syllis*, so again a replacement name is needed to avoid synonymy with *Syllis caeca* Monro, 1933. *Syllis katzmanni* Arvanitidis, 2017 nom. nov. is herewith proposed as a replacement name for *Syllis caeca* (Katzmann, 1973), a previously unreplaced secondary junior homonym of *Syllis caeca* Monro, 1933. As no synonyms are available to choose a replacement name, the new epithet *katzmanni* was chosen in memory of Werner Katzmann (1943–2004), the author of the original name. Records from the Mediterranean given in the literature without an authority likely all belong to Katzmann's species as the type locality of Monro's species is in the Galapagos islands.

#### Syllis krohnii

Ehlers, 1864

Syllis krohnii Ehlers, 1864 | *Typosyllis krohnii* (Ehlers, 1864)

##### Notes

Type locality: Mediterranean (Kvarner Gulf, Croatia).

#### Syllis cf. mayeri

Musco & Giangrande, 2005

##### Notes

Reported from Greece by Keklikoglou et al. (2013). *Syllis mayeri* is a Caribbean species which was first reported from the Mediterranean (Lebanon) by Aguado and San Martín (2007) as Syllis cf. mayeri. The authors compared their material to the type material and found slight differences in the length of the anterior dorsal cirri and the pharynx. They note that *Syllis mayeri* and other "cosmopolitan" *Syllis* species possibly constitute species complexes. The Greek material corresponds exactly to the description of Aguado and San Martín (2007) and may belong to an undescribed species.

#### Syllis nigricirris

Grube, 1863

Syllis nigricirris Grube, 1863 | Syllis (Typosyllis) nigricirris Grube, 1863

##### Notes

Licher (1999) considers the species as valid, but notes that the characteristic black colour of the dorsal cirri may disappear in ethanol-preserved specimens which would make an unambiguous identification difficult. Greek records are all from before 1987 and were probably identified using Fauvel (1923); the increased use of the keys by San Martín (1984) and San Martín (2003) which do not contain the species could explain the absence of records since then. Type locality: Mediterranean (Adriatic).

#### Syllis parapari

San Martín & López, 2000

##### Notes

Probably recorded under the name *Syllis cornuta* before the year 2000 (see discussion under *Syllis cornuta*). In the Mediterranean also known from Cyprus, (Çınar and Ergen 2003), Turkey (Çınar et al. 2014) and the Adriatic (Mikac 2015), otherwise known from the Atlantic coast of the Iberian Peninsula and from the British Isles (San Martín 2003, San Martín and Worsfold 2015).

#### Syllis prolifera

Krohn, 1852

Syllis prolifera Krohn, 1852 | *Typosyllis prolifera* (Krohn, 1852)

##### Notes

Type locality: Mediterranean.

#### Syllis pulvinata

(Langerhans, 1881)

##### Notes

Reported from Greece by Faulwetter et al. (2011a). Widely distributed in the Mediterranean (Musco and Giangrande 2005).

#### Syllis rosea

(Langerhans, 1879)

#### Syllis rosea magna

(Westheide 1974) sensu Ben-Eliahu 1977

##### Notes

Reported from Greece by Simboura (1996) who considers the species closer to *Syllis rosea rosea* than to the Pacific form o*f Syllis rosea magna* described by Westheide (1974) from the Galapagos Islands. It is likely that the specimens from the Mediterranean belong to neither of the two species but to an undescribed species (Simboura 1996).

#### Syllis torquata

Marion & Bobretzky, 1875

##### Notes

Reported from Greece by Simboura (1996) and Zenetos et al. (1997) (based on the same specimens). Identification confirmed by G. San Martín (pers. comm. in Simboura 1996). Type locality: Mediterranean (Gulf or Marseille, France).

#### Syllis tyrrhena

(Licher & Kuper, 1998)

##### Notes

Reported from Greece by Faulwetter et al. (2011a) based on a single specimen. Type locality: Mediterranean (Tyrrhenian Sea).

#### Syllis variegata

Grube, 1860

Syllis variegata Grube, 1860 | Syllis (Typosyllis) variegata Grube, 1860 | *Typosyllis variegata* (Grube, 1860)

##### Notes

Type locality: Mediterranean (Adriatic).

#### Syllis vittata

Grube, 1840

##### Notes

Type locality: Mediterranean (Palermo, Sicily, Italy).

#### Syllis westheidei

San Martín, 1984

##### Notes

Reported from Greece by Faulwetter et al. (2011a) and Keklikoglou et al. (2013). Type locality: Mediterranean (Balearic Islands).

#### Synmerosyllis lamelligera

(Saint-Joseph, 1886)

Pionosyllis lamelligera Saint Joseph, 1887 | *Synmerosyllis lamelligera* (Saint-Joseph, 1886)

#### Trypanosyllis aeolis

Langerhans, 1879

##### Notes

Reported from Greece by Chatzigeorgiou et al. (2016) under this name, previously reported as *Trypanosyllis gemmipara* Johnson, 1901 by Arvanitidis (2000a), based on works by San Martín (1984) and Campoy (1982). San Martín (2003) synonymises *Trypanosyllis gemmipara* with *Trypanosyllis aeolis* and also refers the two aforementioned descriptions to *Trypanosyllis aeolis*. Álvarez-Campos et al. (2017) re-instate *Trypanosyllis gemmipara* based on molecular analyses and assign them to the genus *Trypanedenta* Imajima & Hartman 1964. *Trypanedenta gemmipara* is probably restricted to the Indo-Pacific region (Alaska to Mexico, Japan, New Zealand, India) and reports from the Mediterranean belong to *Trypanosyllis aeolis* (Álvarez-Campos et al. 2017).

#### Trypanosyllis zebra

(Grube, 1860)

##### Notes

Species complex. Due to its distinct colouration (dark dorsal stripes), *Trypanosyllis zebra* has long been considered a cosmopolitan species. Álvarez-Campos et al. (2017) recovered seven cryptic species worldwide, five of which are described as new species as they can also be morphologically distinguished. The analysis recovered at least three clades in the Mediterranean: *Trypanosyllis krohnii* Claparède, 1864 (re-instated, designated as type species of the genus and used as the preferred name for Mediterranean species), *Trypanosyllis* sp. 2 (differing from *Trypanosyllis krohnii* in bathymetrical distribution) and a clade from Crete (originally identified as *Trypanosyllis zebra* by Chatzigeorgiou et al. (2016), labelled Trypanosyllis cf. krohnii in the analyses). Greek specimens did not have enough support in the analyses to be placed unambiguously. Unfortunately, no material from the type locality of *Trypanosyllis zebra* in the Adriatic Sea was included in the analyses, so it remains unclear how many clades exist in the Mediterranean and whether *Trypanosyllis zebra* and *Trypanosyllis krohnii* are synonymous or distinct species. Until these ambiguities are resolved, all material from Greece is listed under the name *Trypanosyllis zebra*.

#### Virchowia clavata

Langerhans, 1879

Umbellisyllis clavata (Langerhans, 1879) | *Virchowia clavata* Langerhans, 1879

##### Notes

Reported from Greece by Simboura (1996) and Faulwetter et al. (2011a). In the Mediterranean also known from Spain (San Martín 2003), Italy (Castelli et al. 2008) and France (Nygren and Pleijel 2010), otherwise known only from Madeira.

#### Xenosyllis scabra

(Ehlers, 1864)

##### Notes

Possibly a complex of pseudo-cryptic species. Campoy (1982) found differences in chaetal features in specimens of *Xenosyllis scabra* from different localities of the Iberian Peninsula. San Martín et al. (2008) describe two new species from Australia. One of these, *Xenosyllis scabroides* San Martín, Hutchings & Aguado, 2008, differs from *Xenosyllis scabra* only in the shape of the compound chaetae. The authors suggest that material from outside the type locality (Kvarner Gulf, Adriatic) should be carefully examined as it may belong to different, undescribed species.

### Terebellidae Johnston, 1846

#### Amphitrite cirrata

Müller, 1776

#### Amphitrite rubra

(Risso, 1826)

#### Amphitrite variabilis

(Risso, 1826)

##### Notes

Type locality: Mediterranean.

#### Amphitritides gracilis

(Grube, 1860)

#### Amphitritides kuehlmanni

Arvanitidis & Koukouras, 1995

##### Notes

Originally described from the Evoikos Gulf (38°28'55"N, 23°29'10"E, 5 m depth), no other records from Greece.

#### Axionice conchilega

(Pallas, 1766)

Lanice conchilega (Pallas, 1766)

##### Notes

The genus *Lanice* Malmgren, 1866 is considered a synonym of *Axionice* Malmgren, 1866 by Jirkov and Leontovich (2017) based on a phylogenetic analysis of morphological characters. Hutchings et al. (2017) consider the genus *Lanice* valid but could not take the publication by Jirkov and Leontovich (2017) into account as it was published almost concurrently. There is general agreement that several Terebellidae genera are in need of revision (e.g. Matos Nogueira et al. 2013, Jirkov and Leontovich 2017, Hutchings et al. 2017). We here follow the nomenclature of the most recent publication by Jirkov and Leontovich (2017) until more information on the generic affinity of several Terebellidae species becomes available.

#### Axionice cretacea

(Grube, 1860)

Pista cretacea (Grube, 1860)

##### Notes

*Pista cretacea* is considered to belong to *Axionice* by Jirkov and Leontovich (2017). Type locality: Mediterranean (Kvarner Gulf, Adriatic).

#### Axionice medusa

(Savigny in Lamarck, 1818)

Loimia medusa (Savigny in Lamarck, 1818)

##### Ecological interactions

###### Native status

Non-native (established)

##### Notes

Considered a species complex by several authors (e.g. Hutchings and Glasby 1995). Originally described from the Red Sea, considered established in the Mediterranean Sea (Zenetos et al. 2017). The genus *Loimia* Malmgren, 1866 is considered a synonym of *Axionice* Malmgren, 1866 by Jirkov and Leontovich (2017), but see remarks under *Axionice conchilega*, which apply also to *Loimia*.

#### Eupolymnia nebulosa

(Montagu, 1819)

Eupolymnia nebulosa (Montagu, 1819) | *Polymnia nebulosa* (Montagu, 1819)

##### Notes

Species complex. Different populations of *Eupolymnia nebulosa* show different life cycles (brooding vs. free spawning, Bhaud and Lenaers 1992). Sromek et al. (2016), using molecular methods, recovered four cryptic species in Europe: one along the south coast of Norway and three in the Western Mediterranean (of which two also occur in the Atlantic).

#### Eupolymnia nesidensis

(Delle Chiaje, 1828)

Eupolymnia nesidensis (Delle Chiaje, 1828) | *Polymnia nesidensis* (Delle Chiaje, 1828)

##### Notes

Type locality: Mediterranean (Gulf of Naples).

#### Neoamphitrite affinis

(Malmgren, 1866)

##### Notes

Questionable status. Reported from Greece by Papazacharias (1991) from the North Aegean. In the Mediterranean also reported from the Adriatic (Mikac 2015) and Spain (Alós 1984), otherwise distributed in Arctic waters.

#### Neoamphitrite edwardsi

(Quatrefages, 1865)

Amphitrite edwardsi (Quatrefages, 1865) | *Neoamphitrite edwardsi* (Quatrefages, 1865)

##### Notes

Questionable status. Reported from Greece by Karlou and Melea (1977). In the Mediterranean also known from Spain (Tena et al. 1991), Turkey (Çınar et al. 2014) and the Adriatic (Mikac 2015), otherwise distributed along the Atlantic coasts of Europe. While it is possible that the species occurs in Greece, it has so far only been reported in grey literature and has not been found in the last four decades, therefore its presence in Greece is here considered questionable.

#### Neoamphitrite figulus

(Dalyell, 1853)

Amphitrite johnstoni Malmgren, 1865 | *Neoamphitrite figulus* (Dalyell, 1853)

#### Neoamphitrite groenlandica

(Malmgren, 1866)

Amphitrite groenlandica Malmgren, 1866

##### Notes

Questionable status. In the Mediterranean only reported from Greece (Bogdanos and Satsmadjis 1983, Nicolaidou and Papadopoulou 1989). Distributed in the North Atlantic.

#### Nicolea venustula

(Montagu, 1819)

#### Nicolea zostericola

Ørsted, 1844

#### Pista cristata

(Müller, 1776)

Pista cristata (Müller, 1776) | *Pista lornensis* (Pearson, 1969)| *Scionella lornensis* Pearson, 1969

##### Notes

*Scionella lornensis* was synonymised with *Pista cristata* (Müller, 1776) by Jirkov and Leontovich (2017). However, both *Scionella lornensis* and *Pista cristata* are reported in the same publication by Arvanitidis (2000a) (who identified them using the key and descriptions by Holthe (1986)). Mikac and Hutchings (2017), in a paper published concurrently with the work by Jirkov and Leontovich (2017), consider several European records of *Scionella lornensis* (including those from Greece) as potential misidentifications and possibly belonging to either *Pista adriatica* Mikac & Hutchings, 2017 or *Pistella rovignensis* Mikac & Hutchings, 2017.

#### Proclea graffii

(Langerhans, 1884)

##### Notes

Questionable status. Reported from Greece by Bogdanos and Satsmadjis (1983) and NCMR (1995). In the Mediterranean also reported from the Adriatic (Mikac 2015), otherwise distributed in the Arctic. Given the rarity of records in the Mediterranean and the native distribution range of the species, the Greek records are considered questionable.

#### Terebella ehrenbergi

Gravier, 1906

##### Ecological interactions

###### Native status

Non-native (questionable)

##### Notes

Questionable status. Reported from Greece by Arvanitidis (1994). Originally from the Red Sea, in the Mediterranean also reported from the western Mediterranean (Pérès 1954), Cyprus (Ben-Eliahu 1972b) and Israel (Ben-Eliahu 1976b). The taxonomy of the species is confused, as two different descriptions exist in literature: *Terebella ehrenbergi* sensu Day, 1967 and *Terebella ehrenbergi* sensu Rullier, 1972, differing in the shape of the notochaetae (denticulated / smooth) and the number of abdominal chaetigers with notochaetae (posterior 20–40 segments without notochaetae vs. posterior 10–15 segments) (Ben-Eliahu 1976b). Ben-Eliahu (1976b) compared material from the Israeli coasts of the Mediterranean with museum specimens from the Red Sea identified by Gravier and by Fauvel and found the former to lack calyx-type chaetae on abdominal chaetigers. However, as this may be a size-dependent character, it is currently unknown whether Mediterranean species belong to *Terebella ehrenbergi* Gravier, 1906 or to a different species.

#### Terebella lapidaria

Linnaeus, 1767

### Telothelepodidae Nogueira, Fitzhugh & Hutchings, 2013

#### Parathelepus collaris

(Southern, 1914)

### Thelepodidae Hessle, 1917

#### Streblosoma bairdi

Malmgren, 1866)

#### Thelepus cincinnatus

(Fabricius, 1780)

#### Thelepus setosus

(Quatrefages, 186)

##### Notes

Often regarded as cosmopolitan, but probably many of the worldwide records are misidentifications (Hutchings and Glasby 1987).

#### Thelepus triserialis

(Grube, 1855)

##### Notes

Type locality: Mediterranean.

### Trichobranchidae Malmgren, 1866

#### Terebellides stroemii

Sars, 1835

##### Notes

Questionable status. Species complex. *Terebellides stroemii* is a species complex of pseudo-cryptic species (Parapar and Hutchings 2014). Çınar (2005) re-examined some specimens from Cyprus previously identified as *Terebellides stroemii* and compared them to material collected near the type locality. He concluded that two or three different species of *Terebellides* occur along the coast of Cyprus. Parapar et al. (2013) re-investigated material from the Adriatic, found *Terebellides gracilis* (Malm, 1874) to be present in the area and additionally described a new species, *Terebellides mediterranea* Parapar, Mikac & Fiege, 2013. Thus, specimens reported in the Mediterranean under the name *Terebellides stroemii* certainly belong to different species.

#### Trichobranchus glacialis

Malmgren, 1866

### Typhloscolecidae Uljanin, 1878

#### Travisiopsis lanceolata

Southern, 1910

##### Notes

Reported from Greece by Wesenberg-Lund (1939a). In the Mediterranean also known from the Tyrrhenian Sea (Wesenberg-Lund 1939a) and the Adriatic (Mikac 2015), otherwise distributed in the North and South Atlantic (Dales and Peter 1972).

## Analysis

**Figure 2.**
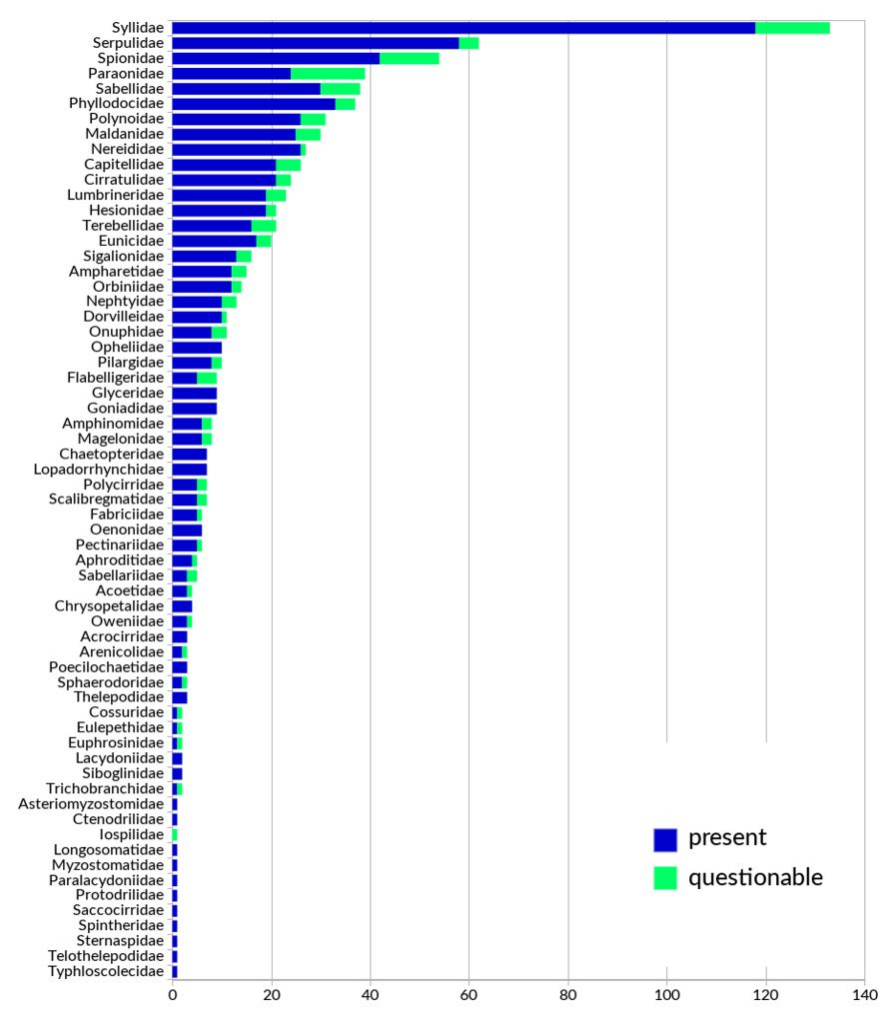
Numbers of polychaete species per family in Greece and their status (present / questionable).

The present work comprises an updated and annotated inventory of polychaete species from Greek waters, compiled from literature reports, online databases, museum collections and unpublished datasets. In total, 999 species-level taxa have been reported from Greece since the first studies in 1832. Of these, 84 are currently considered absent from the country’s waters (Table 1), 79 are heterotypic synonyms and the presence of a further 142 species is considered questionable (Fig. 2). These questionable species are included in the present inventory until additional evidence becomes available to justify their exclusion. Fourteen species are reported here for the first time from Greek waters. At least 52 species in the present list constitute in fact a complex of cryptic or pseudo-cryptic species. Forty-seven species are considered non-native to the area, ten of which are reported here for the first time for the country (Table 2). In addition to the species-level taxa reported in this checklist, eleven genera have been recorded from Greece with no representatives identified to species level (Table 3).

**Figure 3.**
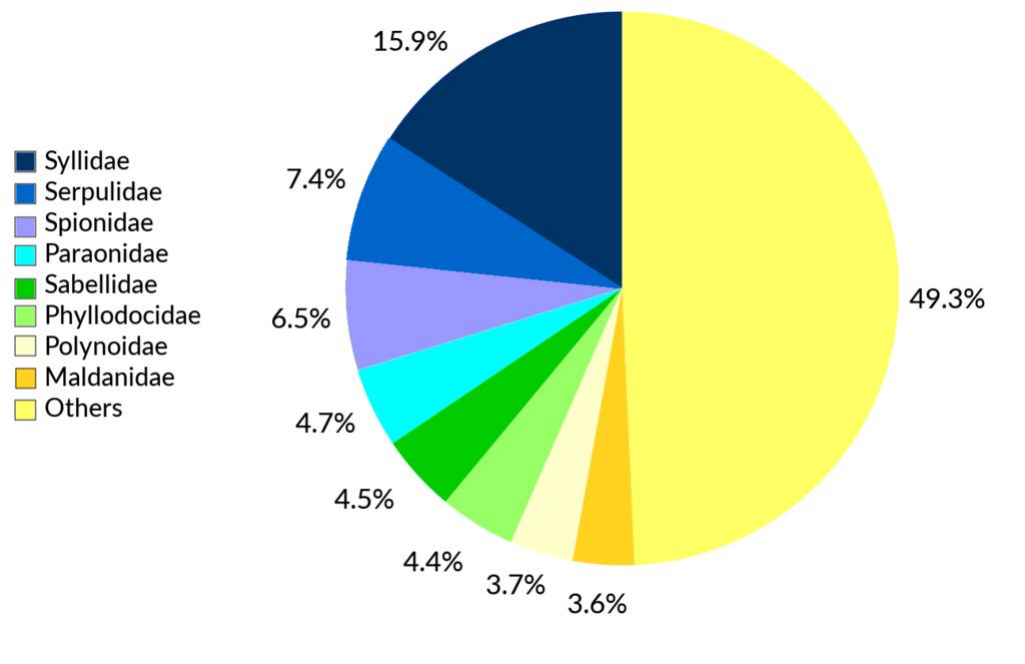
Percentages of species numbers of the eight most species-rich polychaete families in Greek waters compared to the remaining families.

Of the 836 species-level taxa reported from Greece and not explicitly excluded from this checklist, Syllidae are the most species-rich family (133 species / 15.9% of all species), followed by the Serpulidae (62 / 7.4%), Spionidae (54 / 6.5%), Paraonidae (39 / 4.7%) , Sabellidae (38 / 4.5%), Phyllodocidae (37 / 4.4%), Polynoidae (31 / 3.7%) and Maldanidae (30 / 3.6%). Together, these eight families constitute 50.7% of the Greek polychaete species richness (Fig. 3). Twenty-six families are represented by less than 5 species and 17 families have never been reported from Greece: Aberrantidae Wolf, 1987, Alvinellidae Desbruyères & Laubier, 1986, Antonbruunidae Fauchald, 1977, Dinophilidae Remane, 1925, Hartmaniellidae Imajima, 1977, Histriobdellidae Vaillant, 1890, Ichthyotomidae Eisig, 1906, Iphionidae Kinberg, 1856, Laetmonectidae Buzhinskaya, 1986, Nerillidae Levinsen, 1883, Parergodrilidae Reisinger, 1925, Pontodoridae Bergström, 1914, Protodriloididae Purschke & Jouin, 1988, Psammodrilidae Swedmark, 1952, Travisiidae Hartmann-Schröder, 1971, Trochochaetidae Pettibone, 1963 and Uncispionidae Green, 1982.

**Figure 4.**
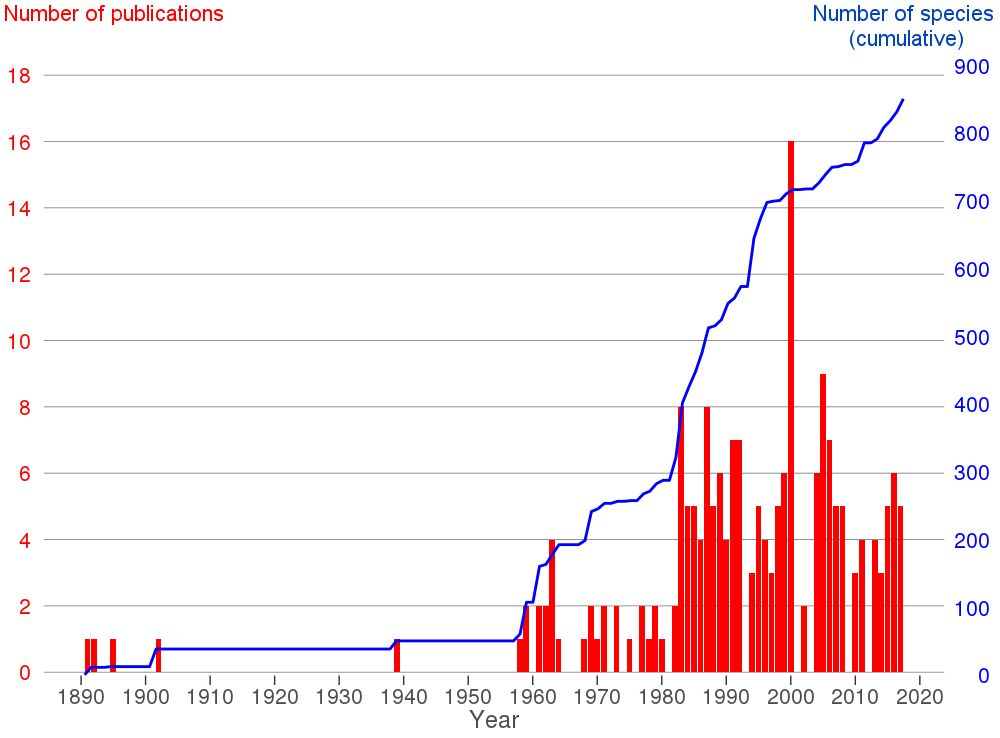
Cumulative numbers of known polychaete records from the Greek EEZ (blue line), superimposed on the number of publications containing polychaete records (red bars). Checklists and other secondary works are excluded.

The Greek polychaete fauna was poorly studied until the 1960-70s, with only occasional publications and a total number of 108 species reported from Greek waters until 1960. Subsequently, marine research was performed much more systematically and studies on polychaete communities were published frequently, adding a substantial number of species to the national faunal inventory (Fig. 4). Up to date, a total of 18 species-level taxa new to science were described from the country’s waters (Table 4). Despite the continuous research on benthic communities, 285 species (34.1%) have been only reported once or twice from Greek waters and 90 species have not been reported since 1993 (Table 5, Suppl. material 2). Since the publication of the last checklist of Greek polychaetes by Simboura and Nicolaidou (2001), 102 species were newly recorded from Greek waters. Another 53 species, reported from Greece before 2001 but not listed by Simboura and Nicolaidou (2001), were recovered from literature as a result of increased availability of electronic resources and the inclusion of habitats not covered previously (deep sea, pelagic waters).

The present list reports 836 species from Greece (including those considered questionable). This corresponds to 75.6% of the species reported by Martin and Gil (2010) from the Mediterranean (1,105 species, standardised to currently accepted names). Simboura and Nicolaidou (2001) report 753 species from Greece vs. 1,015 species from the Mediterranean, corresponding to 74.18%. Martín and Gil (2010) list 359 species in the Mediterranean checklist that are not known from Greece, whereas the Greek checklist contains 67 species not contained in the Mediterranean checklist (excluding questionable species and those described after 2010). Compared to the most recent (and thus comparable) inventories from neighbouring countries and regional seas, Greece shows similar species numbers to Italy (872 species, Castelli et al. 2008) and slightly higher numbers than the Adriatic Sea (756 species, Mikac 2015) and the Mediterranean coasts of Turkey (excluding the Sea of Marmara; 641 species, Çınar et al. (2014); all lists standardised to currently accepted names and including species indicated as questionable). Species unique to each of these checklists (not contained in any of the other three checklists) number 147 for Greece, 48 for the Adriatic, 135 for Italy and 70 for the Turkish Mediterranean (excluding the Sea of Marmara).

## Discussion

Advanced imaging and molecular techniques have given a boost to polychaete systematics and taxonomy. This has not only resulted in up-to-date taxonomic descriptions, reviews and identification keys but also in a refined understanding of regional polychaete diversity and biogeography. Many polychaete species used to be considered cosmopolitan or widely distributed (e.g. Hutchings 1998). Lately, however, there is increasing evidence that these widely distributed species actually comprise endemic forms and cryptic species. Thus, many species seem to be restricted to much smaller geographical areas than was previously assumed (examples include *Chone duneri* (Tovar-Hernández et al. 2007), *Cirratulus cirratus* (Weidhase et al. 2014), *Terebellides stroemii* (Parapar et al. 2013), *Marphysa sanguinea* (Hutchings et al. 2012), *Inermonephtys inermis* (Ravara et al. 2010), *Myriochele heeri* (Parapar 2006), *Pholoe minuta* (Barnich and Fiege 2003), *Magelona cincta* (Wilson 1958)) and many of these species are probably absent from the Mediterranean.

One reason for worldwide reports of many species and thus their assumed cosmopolitanism lies in the use of inadequate literature for the identification of species (Hutchings 1998, Klautau et al. 1999). This is also the case in the Mediterranean, where many of the reported “cosmopolitan” or boreal species were identified using literature covering non-Mediterranean species. Thus, especially reports from older literature need to be treated with care, as many of these specimens may in fact belong to different, possibly endemic, species. Several Mediterranean taxa display morphological differences compared to material from the native distribution range of the species they were originally assigned to, indicating the existence of endemic forms (e.g. *Ophelia roscoffensis* Augener, 1910, *Galathowenia oculata* (Zachs, 1923), *Syllis rosea magna* (Westheide 1974) sensu Ben-Eliahu 1977, *Aphelochaeta monilaris* (Hartman, 1960) or *Therochaeta flabellata* (Sars in Sars, 1872)). Increasingly, many of these taxa are being revised and either described as species new to science or re-instated as previously synonymised Mediterranean species (e.g. *Acromegalomma lanigerum* (Grube, 1846), *Sternaspis thalassemoides* Otto, 1821, *Pherusa mikacae* Salazar-Vallejo, 2014, *Prionospio maciolekae* Dağlı and Çınar 2011, *Syllis parapari* San Martín & López, 2000 or *Terebellides mediterranea* Parapar, Mikac & Fiege, 2013). Over the last years, the increasing availability of updated keys which employ reliable, unique and age-independent diagnostic characters have allowed for a much finer distinction between species and have led to many reports of species new for Greece or the Mediterranean.

Another factor causing uncertainty, concerning the diversity and distribution of polychaete species worldwide, is the increasing discovery of cryptic species through molecular methods (Nygren 2014). Carr et al. (2011) show that polychaete diversity in Canadian waters is highly underestimated, as molecular analyses have revealed one quarter of the 333 morphologically recognised species to actually comprise two or more putative cryptic species. Similarly, many species occurring in the Mediterranean have been shown to actually form complexes of genetically distinct species within the basin (e.g. *Eulalia viridis* (Linnaeus, 1767), *Eurythoe complanata* (Pallas, 1776), *Lysidice ninetta* Audouin & Milne Edwards, 1833, *Myxicola infundibulum* Montagu, 1808, *Ophryotrocha puerilis* Claparède & Mecznikow, 1869, *Perinereis cultrifera* Grube, 1840, *Platynereis dumerilii* (Audouin & Milne Edwards, 1834), *Syllis gracilis* Grube, 1840 *or Trypanosyllis zebra* (Grube, 1860)). As only few of these studies include material from Greece, it remains unknown whether Greek populations of these species actually belong to the nominal taxon or to a different sibling species within the complex. Currently, 51 names (6.1%) of the Greek inventory are considered to refer to species complexes and resolving them will lead to major advances concerning our knowledge of the polychaete fauna in Greece and the Mediterranean.

This increased research effort contributes to an enhanced understanding of polychaete diversity, systematics and distribution but also introduces a high level of uncertainty for legacy records. The re-instatement or description of new species from a complex of cryptic or pseudo-cryptic species invariably renders most literature records under this name questionable, as they could belong to either of the species within this complex (e.g. S*calibregma inflatum* Rathke, 1843, *Spiophanes reyssi* Laubier, 1964, *Stylarioides monilifer* Delle Chiaje, 1841, *Sternaspis scutata* Ranzani, 1817, *Branchiosyllis exilis* (Gravier, 1900) or *Haplosyllis spongicola* (Grube, 1855)). However, in some cases the identity of these records can be resolved *a posteriori*, if information on the literature used for identification is available. A general recommendation of good practice is, therefore, to always provide a list of the literature used for identification alongside any published list of species. Similarly, revisionary publications and species lists should, where possible, report not only the currently accepted name but also the subjective synonyms under which the species were originally recorded. Failing to do so may lead to incorrect distribution records if the synonymised species is re-instated later (e.g. see reports of the non-native species *Cossura delta* Reish, 1958, *Leocrates chinensis* Kinberg, 1866, *Loimia arborea* Moore, 1903 and *Nematonereis hebes* Verrill, 1900 from Greece (Table 1) which had originally been recorded as *Cossura soyeri* Laubier, 1964, *Leocrates claparedii* (Costa in Claparède, 1868), *Loimia medusa* (Savigny in Lamarck, 1818) and *Nematonereis unicornis* (Grube, 1840) but were reported from Greece using the former names which were accepted at that time).

Despite a high number of changes compared to the last published checklist (Simboura and Nicolaidou 2001), the Greek polychaete fauna continues to represent ca. 75% of the Mediterranean inventory (74.2% in 2001, 75.6% this study). However, many families remain understudied in Greece and have only been reported at genus level or not at all. Most of these families include species associated with certain habitats which in turn are understudied, such as deep sea, pelagic and interstitial habitats and hard substrates (especially coralligenous formations and caves). More research effort needs to be targeted towards these habitats, as the knowledge on these communities is of the utmost importance for ecological studies assessing ecosystem functioning, food webs, habitat conservation and changes caused by invasions of non-native species (to which Greece is particularly exposed due to its geographic location). In addition, as morphology alone underestimates species richness (Nygren 2014), more molecular studies are needed to resolve the identity of species complexes in Greece.

The number of species from Greece is similar to those of neighbouring areas from which recent checklists are available. However, individual checklists are influenced by research effort, different criteria for the inclusion or exclusion of species and the application of different species concepts. Thus, these comparisons can only serve as rough estimates of biogeographic relatedness and species richness. Nevertheless, each of these inventories comprises a relatively large number of names not reported from other areas. This can be partly explained by the aforementioned factors, partly by the actual biogeographic distribution and partly by incorrect species identifications. The Greek checklist contains 40 species that are of non-Mediterranean origin and have – to the best of our knowledge – not been reported from other areas in the Mediterranean. Where these species have only been found once or only by the same authors, misidentifications or an author bias must be assumed.

In fact, regional patterns of polychaete diversity in the Mediterranean seem to be biased by researcher identity (Chatzigeorgiou et al. 2017) and existing inventories are characterised by a large number of questionable species (e.g. 23.7% of the Mediterranean inventory (Arvanitidis 1994); 16% of the current Greek inventory). Geographically inappropriate or outdated literature contributes to this confusion (e.g. Hutchings 1998). Several updated keys and revisions for southern European polychaetes have been published recently (e.g. the Fauna Ibérica series) and the internet greatly facilitates access to literature. Nevertheless, comprehensive revisions and updated identification keys are lacking for many taxa in the Mediterranean and the use of century-old treatises such as those by (Fauvel 1923, Fauvel 1927) as the main source for species identification is still common practice in many laboratories.

The Mediterranean Sea is a hotspot of biodiversity and at the same time subject to a range of vectors of change such as habitat loss and degradation, climate change, pollution and invasion of alien species (Coll et al. 2010). To estimate the impact of these changes on biodiversity and ecosystem functioning, accurate knowledge of species’ spatio-temporal distribution patterns is of crucial importance. The observed high levels of uncertainty regarding the regional fauna provide obstacles to such investigations and need to be resolved. The problem could be addressed through the organisation of regular pan-Mediterranean training and intercalibration workshops. These ring tests would train technicians and scientists in the identification of difficult or rarely encountered groups, calibrate and improve identification skills across areas and taxonomic groups and should result in a list of recommended and frequently updated identification literature and resources.

## Supplementary Material

Supplementary material 1 List of unpublished / online resources Data type: Literature list
Brief description: A list of references (unpublished datasets by the Hellenic Centre for Marine Research) and online resources which were used to assess the occurrence status of many species. This list is merely for completeness and for the convenience of the reader.
File: oo_172923.pdf Nomiki Simboura, Sarah Faulwetter

Supplementary material 2 List of species per literature resource Data type: References for species occurrence records in Greece, *.csv format, tab-separated
Brief description: Contains a list of all consulted literature references with polychaete records in Greece. Note that a) the list of polychaetes for a few sources may be incomplete as only rare or new records were noted and b) that records mentioned in two or more sources may in some cases pertain to the same specimen, either because the same dataset was used in several publications or because records were repeated in secondary literature (checklists, databases).
Explanation of columns: *Family*: Family the taxon belongs to; *ScientificName_accepted*: Currently (December 2017) accepted scientific name for the taxon; *Rank*: taxonomic rank; *ScientificAuthority_accepted*: Currently (December 2017) accepted scientific authority for the taxon; *ScientificName_asReported*: Taxon as reported in the given literature /data resource, standardised with WoRMS to remove spelling mistakes and typographic errors; *ScientificAuthority_asReported*: Scientific authority as reported in the given literature /data resource, standardised with WoRMS to remove spelling mistakes and typos; *Source_authors*: Authors of literature/data resource; *Source_year*: Year of literature/data resource; *Source_reference*: Other bibliographic info (title, journal, URL, etc) of literature/data resource; *Status_in_Greece*: current status of the taxon in Greece (present/absent/questionable); *Occurrence_record_notes*: information on the specific taxon-resource combination, e.g. misidentifications of the specific specimens;
File: oo_174725.csv Sarah Faulwetter, Nomiki Simboura, Nikolaos Katsiaras, Georgios Chatzigeorgiou, Christos Arvanitidis

## Figures and Tables

**Table 1.** Species reported from Greece in previous publications but in fact absent from the area, with unclear identity (e.g. nomina dubia), or wrongly applied names.

**Family**	**Scientific name**	**Reported as**	**Remarks**
Acoetidae	*Euarche tubifex* Ehlers, 1887	*Euarche tubifex*	Arvanitidis (2000a) lists Aegean Sea records of *Eupanthalis kinbergi* McIntosh, 1876 under the name *Euarche tubifex*; subsequently the name was included in the Greek checklist by Simboura and Nicolaidou (2001). Arvanitidis (2000a) follows Pettibone (1989) who assigns many Mediterranean records of *Eupanthalis kinbergii*, including those of Fauvel (1923), to *Euarche tubifex*. While it is highly likely that *Euarche tubifex* is present in Greece, it could not yet be confirmed.
Ampharetidae	*Amage gallasii* Marion, 1875	*Amage gallasii*	Reported by Arvanitidis (2000a) for the Aegean Sea based on records from the Turkish Aegean (Ergen 1987); subsequently erroneously included in the Greek checklist by Simboura and Nicolaidou (2001).
Capitellidae	*Dasybranchus carneus* Grube, 1870	*Dasybranchus carneus*	Reported by Arvanitidis (2000a) for the Aegean Sea based on records from the Turkish Aegean (Ergen 1987); subsequently erroneously included in the Greek checklist by Simboura and Nicolaidou (2001).
Cirratulidae	*Timarete anchylochaeta* (Schmarda, 1861)	*Timarete anchylochaeta*	Reported by Arvanitidis (2000a) for the Aegean Sea based on records from the Sea of Marmara (Rullier 1963); subsequently erroneously included in the Greek checklist by Simboura and Nicolaidou (2001).
Cirratulidae	*Timarete dasylophius* (Marenzeller, 1879)	*Timarete dasylophius*	Reported by Arvanitidis (2000a) for the Aegean Sea based on records from the Sea of Marmara (Rullier 1963); subsequently erroneously included in the Greek checklist by Simboura and Nicolaidou (2001).
Cossuridae	*Cossura delta* Reish, 1958	*Cossura delta*	Reported by Faulwetter (2010). Records of *Cossura soyeri* Laubier, 1964 were reported as *Cossura delta*, as this was the accepted name in WoRMS at the time of compilation.
Eunicidae	*Eunice oerstedii* Stimpson, 1853	*Eunice oerstedii*	European specimens could belong to *Eunice dubitata* Fauchald, 1974 or a closely-related species (Wisnens 1989). The available descriptions of *Eunice oerstedii* are confusing and probably based on different species, thus its status was considered indeterminable by Fauchald (1992). Not listed in the key to global *Eunice* species by Carrera-Parra and Salazar-Vallejo (1998).
Eunicidae	*Eunice stigmatura* (Verril, 1990)	*Eunice stigmatura*	Recorded from a single specimen by Chatzigeorgiou et al. (2016) which was re-examined and found to be an incomplete and unidentifiable specimen.
Eunicidae	*Marphysa disjuncta* Hartman, 1961	*Marphysa disjuncta*	Reported from Greece by Simboura et al. (2010). However, Kurt Şahin (2014) doubts its presence in the Mediterranean after re-examination of available material, including some of the Greek specimens of the former authors.
Eunicidae	*Lysidice hebes* (Verrill, 1900)	*Nematonereis hebes*	Reported by Faulwetter (2010). Records of *Lysidice unicornis* (Grube, 1840) were reported as *Nematonereis hebes*, as this was the accepted name in WoRMS at the time of compilation.
Fabriciidae	*Pseudofabriciola capensis* (Monro, 1937)	*Pseudofabriciola capensis*	Records in OBIS (Arvanitidis 2000b) could not be traced back to any source and are believed to be a misidentification or database artefact.
Fabriciidae	*Pseudofabriciola filamentosa* (Day, 1963)	*Fabricia filamentosa*	Fitzhugh et al. (1994) re-examined all specimens identified previously as *Fabricia filamentosa* from Greece (Simboura 1990) and described them as a new species, *Pseudofabriciola longipyga* Fitzhugh, Giangrande & Simboura, 1994.
Glyceridae	*Glycera alba adspersa* Fauvel, 1939	*Glycera alba adspersa*	Reported by Faulwetter (2010), but in fact has only been recorded by Rullier (1963) from the Bosporus and not from Greece. Considered indeterminable by Böggemann (2002).
Glyceridae	*Glycera papillosa* Grube, 1857	*Glycera papillosa*	Considered indeterminable by Böggemann (2002). The holotype is lost and, based on the original description, it could be a synonym of either *Glycera capitata* Ørsted, 1843 or *Glycera lapidum* Quatrefages, 1866.
Hesionidae	*Leocrates chinensis* Kinberg, 1866	*Leocrates chinensis*	The Mediterranean species *Leocrates claparedii* (Costa in Claparède, 1868) was considered synonymous with *Leocrates chinensis* by Hartman (1965a), the synonymy was revoked by Pleijel (1998). Greek specimens were initially identified as *Leocrates claparedii* using the key by Fauvel (1923) but reported under the name *Leocrates chinensis*. The latter name has survived in checklists and databases of introduced species (e.g. Zenetos et al. 2010, ELNAIS), but the specimens behind these records actually belong to the native species *Leocrates claparedii*.
Hesionidae	*Neogyptis rosea* (Malm, 1874)	*Gyptis rosea*	Recorded by Chatzigeorgiou et al. (2016), but the specimen was re-examined and actually belongs to *Neogyptis mediterranea* (Pleijel, 1983).
Hesionidae	*Orseis pulla* Ehlers, 1864	*Orseis pulla*	Considered indeterminable by Pleijel (1998).
Longosomatidae	*Heterospio reducta* Laubier, Picard & Ramos, 1973	*Heterospio reducta*	Records in OBIS (Arvanitidis 2000b) could not be traced back to any source and is believed to be a misidentification or database artefact.
Lumbrineridae	*Lumbrineris labrofimbriata* Saint-Joseph, 1888	*Lumbrineris labrofimbriata*	Species considered a juvenile form and thus invalid by Carrera-Parra (2006). Not listed as a Mediterranean species in recent regional reviews (Carrera-Parra et al. 2011, D’Alessandro et al. 2016).
Lumbrineridae	*Lumbrineris cingulata* Ehlers, 1897	*Lumbrineris cingulata*	Reported by Chatzigeorgiou et al. (2016), but specimens were re-examined and actually belong to *Lumbrineris luciliae* Martins, Carrera-Parra, Quintino & Rodrigues, 2012.
Lumbrineridae	*Lumbrineris perkinsi* Carrera-Parra 2001	*Lumbrinerides perkinsi*	Name applied by Simboura et al. (2010) to literature records of *Lumbrineris inflata* Moore, 1911, based on conclusions by Çınar (2009). However, specimens subsequently identified as *Lumbrineris perkinsi* (unpublished data) were re-examined and found to belong to *Gallardoneris iberica* thus the presence of *Lumbrineris perkinsi* in Greece is not confirmed.
Lumbrineridae	*Scoletoma debilis* (Grube, 1878)	*Scoletoma debilis*	Reported by Arvanitidis (2000a) for the Aegean Sea based on records from the Turkish Aegean and the Sea of Marmara (Ergen 1987, Rullier 1963); subsequently erroneously included in the Greek checklist by Simboura and Nicolaidou (2001).
Nephtyidae	*Nephtys longosetosa* Ørsted, 1842	*Nephtys longosetosa*	Reported by Arvanitidis (2000a) for the Aegean Sea based on records from the Turkish Aegean (Ergen 1987); subsequently erroneously included in the Greek checklist by Simboura and Nicolaidou (2001).
Nephtyidae	*Nephtys paradoxa* Malm, 1874	*Nephtys paradoxa*	Reported by Arvanitidis (2000a) for the Aegean Sea based on records from the Turkish Aegean (Ergen 1987); subsequently erroneously included in the Greek checklist by Simboura and Nicolaidou (2001).
Nereididae	*Neanthes arenaceodentata* (Moore, 1903)	*Nereis arenaceodonta*	Multiple records in OBIS which are database artefacts. Originally recorded as *Nereis caudata* (sensu Delle Chiaje, 1827), which was listed as a synonym of *Nereis arenaceodentata* in WoRMS when datasets were submitted to OBIS. The latter name was erroneously used to standardise the taxonomy.
Oenonidae	*Arabella semimaculata* (Moore, 1911)	*Arabella semimaculata*	Reported by Chatzigeorgiou et al. (2016), but the specimen was re-examined and actually belongs to *Arabella iricolor* (Montagu, 1804).
Opheliidae	*Ophelia neglecta* Schneider, 1892	*Ophelia neglecta*	Specimens reported in OBIS belong in fact to *Ophelia roscoffensis* Augener, 1910.
Paraonidae	*Aricidea nolani* Webster & Benedict, 1887	*Aricidea nolani*	Considered indeterminable (Hartley 1981), as the type material consists of fragments of two different species.
Paraonidae	*Paraonides neapolitana* (Cerruti, 1909)	*Cirrophorus neapolitanus; Paraonides neapolitana; Paraonis neapolitana*	Considered of uncertain identity, as the original description may be based on artefacts resulting from the fixation method and the type material is lost (see Langeneck et al. (2017a) for detailed information).
Paraonidae	*Levinsenia flava* (Strelzov, 1973)	*Levinsenia flava*	Greek specimens were re-examined by A. Castelli and identified tentatively as Levinsenia cf. oculata (Hartman, 1957), but possibly belong to an undescribed species.
Phyllodocidae	*Lugia pterophora* (Ehlers, 1864)	*Lugia pterophora*	Considered indeterminable by Pleijel (1991) as the original description is probably based on juveniles and the type material is lost.
Phyllodocidae	*Paranaitis capensis* (Day, 1960)	*Paranaitis capensis*	Considered an indeterminable species of Paranaitis by Kato and Pleijel (2003) who examined the holotype and found it in a poor condition preventing its identification.
Phyllodocidae	*Phyllodoce lamelligera* (Gmelin in Linnaeus, 1788)	*Anaitides lamelligera*; *Phyllodoce lamelligera*	The species and its authorship are confused. The species was described by Gmelin in Linnaeus (1788), citing a "*Nereis lamellifera* Pallas 1788" but introducing a misspelling (*lamellifera* became *lamelligera*). Fauvel (1923) lists "Johnston, 1865" as the author, although Johnston (1865) merely reports the species from the UK. Both original descriptions (Gmelin and Pallas) could fit a variety of species. Therefore, Pleijel (1991) considers the species (listed by him as *Nereis lamellifera*) indeterminable (see also Mikac 2015). Alós (2004) lists *Phyllodoce lamelligera* Johnston 1865 as a synonym of *Phyllodoce madeirensis* Langerhans, 1880, presumably on the basis of the description by Fauvel (1923), as the same incorrect authority is given.
Phyllodocidae	*Phyllodoce schmardaei* Day, 1963	*Phyllodoce macrophthalma*	*Phyllodoce macrophthalma* Schmarda, 1861 is a junior homonym to *P. macrophthalma* Grube 1856. Day (1963) described *Phyllodoce macrophthalma* Schmarda into synonymy with it. Pleijel (1991), however, considers both *Phyllodoce macrophthalma* Schmarda and *Phyllodoce macrophthalma* Grube nomina dubia.
Phyllodocidae	*Protomystides bidentata* (Langerhans, 1880)	*Mystides bidentata; Protomystides bidentata*	Considered indeterminable by Pleijel (1991) due to the absence of type material. Alós (2004) provides a description of *Phyllodoce bidentata*, but gives no reference to the basis of the description (no type material or material from the type locality seems to have been studied). Thus, it is not certain whether the species of Alós (2004) is actually the same species as the one described by Langerhans. All Greek records are from before 2004 and therefore need to be re-examined.
Phyllodocidae	*Pterocirrus limbatus* (Claparède, 1868)	*Pterocirrus limbata*	Controversial species with a confused history of descriptions and many misidentifications (Alós 2004). Considered a nomen dubium by Pleijel (1991), re-described by Alós (2004) but not on the basis of type material or material from the type locality. The single record from Greece (Simboura 1996) would need to be re-examined to confirm its identity.
Pilargidae	*Ancistrosyllis cingulata* (Korschelt, 1893)	*Ancistrosyllis cingulata*	Larval form, probably indeterminable even to genus according to modern experts (G .Read in WoRMS). Fiege and Böggemann (1999) do not include it in their revision of the genus.
Pilargidae	*Sigambra constricta* (Southern, 1921)	*Sigambra constricta*	Reported by Arvanitidis (2000a) for the Aegean Sea based on records from the Turkish Aegean and the Sea of Marmara (Ergen 1987, Rullier 1963); subsequently erroneously included in the Greek checklist by Simboura and Nicolaidou (2001).
Poecilochaetidae	*Elicodasia mirabilis* Laubier & Ramos, 1973	*Elicodasia mirabilis*	Nomen dubium, may be a posterior end of *Poecilochaetus fauchaldi* Pilato & Cantone, 1976 (Santos and Mackie 2008).
Polycirridae	*Polycirrus haematodes* (Claparède, 1864)	*Polycirrus hematodes*	Considered of uncertain identity by Glasby and Hutchings (2014) as the type material was not deposited, Claparède's description is insufficient to delimit the species and several species were possibly confused under this name in the past.
Polycirridae	*Polycirrus pallidus* (Claparède, 1864)	*Polycirrus pallidus*	Considered of uncertain identity by Glasby and Hutchings (2014) as the type material is lost or was never deposited.
Polycirridae	*Polycirrus tenuisetis* Langerhans, 1881	*Polycirrus tenuisetis*	Considered of uncertain identity by Glasby and Hutchings (2014) as the type material is lost or was never deposited.
Polynoidae	*Eunoe mammiloba* Czerniavsky, 1882	*Eunoe mammiloba*	Records in OBIS (Dounas 1988) but not contained in original report, databasing error is assumed.
Polynoidae	*Harmothoe johnstoni* (McIntosh, 1876)	*Harmothoe johnstoni*	Indeterminable as the original description and the holotype are incomplete (Barnich and Fiege 2000a, Barnich and Fiege 2009).
Polynoidae	*Harmothoe minuta* (Potts, 1910)	*Harmothoe minuta*	Reported by Arvanitidis (2000a) for the Aegean Sea based on records from the Turkish Aegean and the Sea of Marmara (Ergen 1976, Rullier 1963). Considered indeterminable by Barnich and Fiege (2000a) as the original description and the holotype are incomplete.
Sabellariidae	*Sabellaria alcocki fauveli*	*Sabellaria alcocki fauveli*	Proposed by Arvanitidis (1994) as a new subspecies, but was never validly published and therefore does not constitute an available name. Even if it were available, it would constitute a junior homonym of *Sabellaria fauveli* Gravier, 1908.
Sabellariidae	*Tetreres porrectus* (Ehlers, 1908)	*Lygdamis porrectus*; Lygdamis (Pallasia) porrectus	Reported by Pérès (1959), but the specimen belongs in fact to *Lygdamis muratus* Allen, 1904 (Bhaud 1975, G. Bellan pers. comm. to C. Arvanitidis).
Sabellidae	*Bispira crassicornis* (Sars, 1851)	*Bispira crassicornis; Sabellacrassicornis*	Arvanitidis (2000a) listed Aegean Sea records of *Sabella fabricii* Krøyer, 1856 under the name *Bispira crassicornis* (following Fauvel (1927) in the synonymy but giving priority to the older name). The record in OBIS by Antoniadou (1998) from the North Aegean was originally recorded as *Sabella fabricii* (Antoniadou 2003); the name *Bispira crassicornis* was applied during data management. *Bispira crassicornis* is nowadays considered distinct from *Sabella fabricii* (Knight-Jones and Perkins 1998).
Sabellidae	*Bispira mariae* Lo Bianco, 1893	*Bispira mariae*	Reported by Arvanitidis (2000a) for the Aegean Sea based on records from the Turkish Aegean (Knight-Jones et al. 1991); subsequently erroneously included in the Greek checklist by Simboura and Nicolaidou (2001).
Sabellidae	*Bispira viola* (Grube, 1863)	*Bispira viola*	Reported by Arvanitidis (2000a) for the Aegean Sea based on records from the Turkish Aegean (Knight-Jones et al. 1991); subsequently erroneously included in the Greek checklist by Simboura and Nicolaidou (2001).
Sabellidae	*Branchiomma moebii* Knight-Jones, 1994	*Branchiomma moebii*	Reported by Arvanitidis (2000a) for the Aegean Sea based on records from the Turkish Aegean (Knight-Jones et al. 1991); subsequently erroneously included in the Greek checklist by Simboura and Nicolaidou (2001).
Sabellidae	*Chone kroyeri* Sars, 1862	*Chone kroyeri*	Records in OBIS (Zenetos 1986) were subsequently found to be a misidentification.
Sabellidae	*Potamilla neglecta* (Sars, 1851)	*Potamilla neglecta*	Reported by Koukouras et al. (1992), record was referred to *Demonax brachychona* (Claparède, 1870) by Arvanitidis (1994).
Scalibregmatidae	*Polyphysia crassa* (Ørsted, 1843)	*Polyphysia crassa*	Reported by Simboura (1996) but the specimen belongs in fact to the Mediterranean subspecies *Polyphysia crassa fauveli* (Laubier, 1959).
Serpulidae	*Hydroides uncinata* (Phillipi, 1844)	*Hydroides uncinata*	Considered indeterminable by Read et al. (2017), as the original taxon description is insufficient to identify the species and the name has been applied to a number of different species in the past (see also notes in WoRMS)
Serpulidae	*Spirorbis corrugatus* (Montagu, 1803)	*Januacorrugatus; Spirorbis (Janua) corrugatus; Spirorbis corrugatus*	*Spirorbis corrugatus* (Montagu, 1803) is an insufficiently described species with a sinistrally coiled tube and type material is not retained (Knight-Jones et al. 1975, Knight-Jones and Knight-Jones 1977). Most authors actually refer to the dextral species described by Caullery and Mesnil (1897) which was re-described by Bush (1905) as *Spirorbis pseudocorrugatus* (now *Neodexiospira pseudocorrugata*). The description of *Spirorbis corrugatus* by Fauvel (1927) refers in fact to *Neodexiospira pseudocorrugata*.
Sigalionidae	*Pholoe fauveli* Kirkegaard, 1983	*Pholoe fauveli*	Kirkegaard (1983) examined specimens of *Pholoe synophthalmica* Claparède, 1868 by Fauvel (1923) and assigned them to *Pholoe fauveli*. Consequently, Arvanitidis (2000a) listed all Greek records of *Pholoe synophthalmica* under the name *Pholoe fauveli*, assuming that all of them were identified using the key by Fauvel (1923). *Pholoe synophthalmica* is nowadays considered a synonym of *Pholoe inornata* Johnston, 1839 and *Pholoe fauveli* is considered absent from the Mediterranean and possibly a synonym of *Pholoe inornata* (Barnich and Fiege 2003).
Spionidae	*Dipolydora caulleryi* (Mesnil, 1897)	*Polydora caulleryi*	Radashevsky and Simboura (2013) examined all Greek material and assigned it to *Dipolydora blakei* (Maciolek, 1984).
Spionidae	*Prionospio malmgreni* Claparède, 1870	*Prionospio malmgreni*	Considered indeterminable by Maciolek (1985) and Sigvaldadóttir and Mackie (1993). Reported to have up to 9 pairs of branchiae in the original description, a character that was never observed again (Sigvaldadóttir and Mackie 1993). The description of *Prionospio malmgreni* by Fauvel (1927) actually belongs to *Prionospio fallax* Söderström,1920 (Sigvaldadóttir and Mackie 1993), thus Mediterranean records probably belong to *Prionospio fallax* (Mikac 2015,Çınar et al. 2014).
Spionidae	*Spio martinensis* Mesnil, 1896	*Spio martinensis*	Record in OBIS (Zenetos 1986) is possibly a misidentification and was later removed from a revised version of the dataset; these corrections were not incorporated in the OBIS data.
Syllidae	*Autolytus roseus* Claparède, 1864	*Autolytus roseus*	Indeterminable, as types do not exist; species probably is the female stolon of *Proceraea madeirensis* Nygren, 2004 (Nygren 2004).
Syllidae	*Dioplosyllis cirrosa* Gidholm, 1962	*Dioplosyllis cirrosa*	Records in OBIS were digitised from Musco and Giangrande (2005) who list it to occur in the Eastern Mediterranean. During digitisation, the centre of the Eastern Mediterranean was georeferenced, which falls into the Greek EEZ. The only Eastern Mediterranean records are from Cyprus (Çınar and Ergen 2003).
Syllidae	*Epigamia macrophtalma* (Marenzeller, 1875)	*Autolytus sardai*	Records in OBIS were digitised from Musco and Giangrande (2005) who list it to occur in the Eastern Mediterranean. During digitisation, the centre of the Eastern Mediterranean was georeferenced, which falls into the Greek EEZ. The only Eastern Mediterranean records are from Cyprus (Çınar et al. 2003).
Syllidae	*Erinaceusyllis belizensis* (Russel, 1989)	*Erinaceusyllis belizensis*	Records in OBIS were digitised from Musco and Giangrande (2005) who list it to occur in the Eastern Mediterranean and Turkey. During digitisation, the centre of the Eastern Mediterranean was georeferenced, which falls into the Greek EEZ and the Turkish record was wrongly placed into the Greek EEZ in the North Aegean. The only Eastern Mediterranean records are from the Turkish Aegean (Çınar and Ergen 2002).
Syllidae	*Eusyllis kupfferi* Langerhans, 1879	*Eusyllis kupfferi*	Records in OBIS were digitised from Musco and Giangrande (2005) who list it to occur in the Eastern Mediterranean. During digitisation, the centre of the Eastern Mediterranean was georeferenced, which falls into the Greek EEZ. The only Eastern Mediterranean records are from Cyprus (Çınar and Ergen 2003) and Turkey (Çınar et al. 2014).
Syllidae	*Inermosyllis balearica* (San Martín, 1982)	*Inermosyllis balearica*	Records in OBIS were digitised from Musco and Giangrande (2005) who list it to occur in the Eastern Mediterranean. During digitisation, the centre of the Eastern Mediterranean was georeferenced, which falls into the Greek EEZ. The only Eastern Mediterranean records are from Lebanon (Aguado and San Martín 2007).
Syllidae	*Myrianida dentalia* (Imajima, 1966)	*Autolytus dentalius*	Records in OBIS were digitised from Musco and Giangrande (2005) who list it to occur in the Eastern Mediterranean. During digitisation, the centre of the Eastern Mediterranean was georeferenced, which falls into the Greek EEZ. The only Eastern Mediterranean records are from Cyprus (Çınar et al. 2003). Nygren (2004) restricts the distribution to the North-West Atlantic and North Pacific.
Syllidae	*Opisthodonta serratisetosa* (López, San Martín & Jiménez, 1997)	*Pionosyllis serratisetosa*	Records in OBIS were digitised from Musco and Giangrande (2005) who list it to occur in the Eastern Mediterranean. During digitisation, the centre of the Eastern Mediterranean was georeferenced, which falls into the Greek EEZ. The only Eastern Mediterranean records are from Cyprus (Çınar and Ergen 2003).
Syllidae	*Parapionosyllis gestans* Pierantoni, 1903	Parapionosyllis cf. gestans	Reported by Simboura (1996) as Parapionosyllis cf. gestans sensu Campoy 1892. Campoy's species was later described as a new species, *Parapionosyllis cabezali* Parapar, San Martín & Moreira, 2000 and Simboura and Nicolaidou (2001) referred the Greek records to the latter.
Syllidae	*Parapionosyllis labronica* Cognetti, 1965	*Parapionosyllis labronica*	Records in OBIS were digitised from Musco and Giangrande (2005) who list it to occur in the Eastern Mediterranean and Turkey. During digitisation, the centre of the Eastern Mediterranean was georeferenced, which falls into the Greek EEZ and the Turkish record was wrongly placed into the Greek EEZ in the North Aegean. The only Eastern Mediterranean records are from the Turkish Aegean (Çınar and Ergen 2002).
Syllidae	*Paraprocerastea crocantinae* San Martín & Alós, 1989	*Paraprocerastea crocantinae*	Records in OBIS were digitised from Musco and Giangrande (2005) who list it to occur in the Eastern Mediterranean and Turkey. During digitisation, the centre of the Eastern Mediterranean was georeferenced, which falls into the Greek EEZ and the Turkish record was wrongly placed into the Greek EEZ in the North Aegean. The only Eastern Mediterranean records are from the Turkish Aegean (Çınar and Ergen 2002).
Syllidae	*Parexogone caribensis* (San Martín, 1991)	*Exogone caribensis*	Records in OBIS were digitised from Musco and Giangrande (2005) who list it to occur in the Eastern Mediterranean and Turkey. During digitisation, the centre of the Eastern Mediterranean was georeferenced, which falls into the Greek EEZ and the Turkish record was wrongly placed into the Greek EEZ in the North Aegean. The only Eastern Mediterranean records are from the Turkish Aegean (Çınar and Ergen 2002).
Syllidae	*Parexogone cognettii* Castelli, Badalamenti & Lardici, 1987	*Exogone cognettii*	Records in OBIS were digitised from Musco and Giangrande (2005) who list it to occur in the Eastern Mediterranean and Turkey. During digitisation, the centre of the Eastern Mediterranean was georeferenced, which falls into the Greek EEZ and the Turkish record was wrongly placed into the Greek EEZ in the North Aegean. The only Eastern Mediterranean records are from the Turkish Aegean (Çınar et al. 2014).
Syllidae	*Prosphaerosyllis longipapillata* (Hartmann-Schröder, 1979)	*Prosphaerosyllis longipapillata*	Records in OBIS were digitised from Musco and Giangrande (2005) who list it to occur in the Eastern Mediterranean. During digitisation, the centre of the Eastern Mediterranean was georeferenced, which falls into the Greek EEZ. The only Eastern Mediterranean records are from Cyprus (Çınar et al. 2003) and Turkey (Çınar 2009).
Syllidae	*Streptospinigera templadoi* (San Martín, 1984)	*Streptosyllis templadoi*	Records in OBIS were digitised from Musco and Giangrande (2005) who list it to occur in the Eastern Mediterranean and Turkey. During digitisation, the centre of the Eastern Mediterranean was georeferenced, which falls into the Greek EEZ and the Turkish record was wrongly placed into the Greek EEZ in the North Aegean. The only Eastern Mediterranean records are from the Turkish Aegean (Çınar and Ergen 2002).
Syllidae	*Syllis heterochaeta* Moore, 1909	*Langerhansia heterochaeta; Syllis heterochaeta*	Records in OBIS are database artefacts: the species recorded in the original datasets is *Tharyx heterochaeta* / *Monticellina heterochaeta* Laubier, 1961; during taxon standardisation, taxa were incorrectly matched only on the specific epithet.
Syllidae	*Syllis hyllebergi* (Licher, 1999)	*Syllis hyllebergi*	Records in OBIS were digitised from Musco and Giangrande (2005) who list it to occur in the Eastern Mediterranean. During digitisation, the centre of the Eastern Mediterranean was georeferenced, which falls into the Greek EEZ. The only Eastern Mediterranean records are from Cyprus and the Israeli coast (Licher 1999)
Syllidae	*Syllis lutea* (Hartmann-Schröder, 1960)	*Syllis lutea*	Reported by Arvanitidis (1994) who used the key and description by San Martín (1984) for identification which actually belong to *Syllis jorgei* San Martín & López, 2000. *Syllis lutea* is absent from the Mediterranean (San Martín 2003). Arvanitidis' specimens are therefore assigned to *Syllis jorgei*.
Syllidae	*Syllis pontxioi* San Martín & López, 2000	*Syllis pontxioi*	Records in OBIS were digitised from Musco and Giangrande (2005) who list it to occur in the Eastern Mediterranean, Cyprus and Turkey. During digitisation, the centre of the Eastern Mediterranean was georeferenced, which falls into the Greek EEZ and the Turkish record was wrongly placed into the Greek EEZ in the North Aegean. The only Eastern Mediterranean records are from the Turkish Aegean (Çınar and Ergen 2002) and from Cyprus (Çınar and Ergen 2003).
Syllidae	*Syllis schulzi* (Hartmann-Schröder, 1960)	*Syllis schulzi*	Reported by Zenetos et al. (2011) as an alien species, with a reference to its first report by Arvanitidis (1994). However, Arvanitidis (1994) lists the species to occur in the Western Mediterranean, not in Greece.
Syllidae	*Syllis vivipara* Krohn, 1869	*Syllis vivipara*	Records in OBIS were digitised from Musco and Giangrande (2005) who list it to occur in the Eastern Mediterranean. During digitisation, the centre of the Eastern Mediterranean was georeferenced, which falls into the Greek EEZ. The only Eastern Mediterranean records are from the Italian Salento (Corriero et al. 2004).
Syllidae	*Trypanedenta gemmipara* (Johnson, 1901)	*Trypanosyllis gemmipara*	Considered a synonym of *Trypanosyllis aeolis* Langerhans, 1897 by San Martín (2003), re-instated by Álvarez-Campos et al. (2017) and possibly restricted to to the Indo-Pacific area. The only Greek specimens reported under this name by Arvanitidis (1994) actually belong to *Trypanosyllis aeolis* (see discussion there).
Syllidae	*Trypanosyllis gigantea* (McIntosh, 1885)	*Trypanosyllis gigantea*	Records in OBIS were digitised from Musco and Giangrande (2005) who list it to occur in the Greek Aegean, based on Licher (1999) who in turn cites Arvanitidis (1994) for this record. However, Arvanitidis (1994) lists the species to occur in the Adriatic, not in the Aegean. The record is believed to be erroneous.
Terebellidae	*Axionice arborea* (Moore, 1903)	*Loimia arborea*	Reported by Faulwetter (2010). Records of *Loimia medusa* (Savigny in Lamarck, 1818) were reported as *Loimia arborea*, as this was the accepted name in WoRMS at the time of compilation. The genus *Loimia* Malmgren, 1866 is considered a synonym of *Axionice* Malmgren, 1866 by Jirkov and Leontovich (2017).

**Table 2.** New reports of non-native species in Greece. Assessment of the establishment status in Greece follows the terminology and definitions by Zenetos et al. (2011).

**Species**	**Reference**	**Date and location of first detection in Greece**	**Origin**	**Other Mediterranean reports**	**Establishment status in Greece**
*Eurythoe complanata*	Chatzigeorgiou et al. (2016)	2008: Alykes, Northern Crete	Caribbean	Cyprus (Ben-Eliahu 1972b), Israel (Ben-Eliahu 1976a, Arias et al. 2013a), Turkey (Ergen and Çınar 1997), Alboran Sea (Arias et al. 2013a)	casual
*Linopherus canariensis*	Chatzigeorgiou et al. (2016)	2007: Alykes, Northern Crete; Elounda, Northern Crete	Atlantic (Canary Islands)	Lebanon (Laubier 1966), Turkey (Ergen and Çınar 1997) (as *Pseudeurythoe acarunculata*), Cyprus (Çınar 2009), Italy (Cosentino and Giacobbe 2010), Egypt (Dorgham et al. 2014)	cryptogenic
*Leiocapitellides analis*	Akoumianaki (2004); Neofitou et al. (2014)	2000-2001: Maliakos Gulf	Red Sea	Egypt (Abd-Elnaby 2009)	casual
*Mediomastus capensis*	Maidanou et al. (2017)	Elounda Bay, Northern Crete	South Africa	Italy (Castelli et al. 2008), Cyprus (Çınar 2005), Spain (Capaccioni-Azzati and El-Haddad 2015)	casual
*Timarete punctata*	Maidanou et al. (2017)	2006: Elounda Bay, Northern Crete	Caribbean	Turkey (Çınar et al. 2014)	casual
*Lumbrinerides neogesae*	Papageorgiou et al. 2006 (unpublished); this study	2002-2003: Elafonisi Island, South-West Crete	South Africa	Italy (Gravina and Cantone 1991)	casual
*Leonnates persicus*	this study	2013: Argolikos Gulf	Indo-Pacific	Israel (Ben-Eliahu 1991b), Turkey (Çınar et al. 2002)	casual
*Neanthes agulhana*	Chatzigeorgiou et al. (2016)	2007: Alykes, Northern Crete; Elounda, Northern Crete	South Africa	Spain (Núñez 2004), Italy (Occhipinti-Ambrogi et al. 2011)	casual
*Lepidonotus tenuisetosus*	Chatzigeorgiou et al. (2016)	2008: Alykes, Northern Crete	Red Sea	Israel and Egypt (Barnich and Fiege 2003), Turkey (Çınar et al. 2014), Adriatic Mikac 2015)	casual
*Exogone breviantennata*	Dando et al. (1995)	1992: Milos	circumtropical	Lebanon (Aguado and San Martín 2007), Turkey (Çınar et al. 2014)	casual

**Table 3.** Taxa reported at genus level with no species-level representatives known from Greece

Genus	**Notes**
*Abyssoninoe* Orensanz, 1990	New record for Greece. Two specimens, off Limnos island NE Aegean, 77–220 m depth, collected in the framework of the Perseus project (Suppl. material 1).
*Acanthicolepis* McIntosh, 1900	Reported by Koukouras et al. (1985).
*Branchiocapitella* Fauvel, 1932	Reported by Vamvakopoulou (1991). Currently considered a synonym of *Capitella* Blainville, 1828 (Magalhães and Bailey-Brock 2012).
*Apistobranchus* Levinsen, 1883	Reported by Eleftheriou et al. (1990), Simboura (1996).
*Fauveliopsis* McIntosh, 1922	Reported by Eleftheriou et al. (1990), Karakassis (1991), Simboura et al. (1995a), Simboura (1996).
*Hipponoe* Audouin & Milne Edwards, 1830	Reported by Simboura et al. (1995b).
*Meiodorvillea* Jumars, 1974	Reported by Simboura (1996).
*Perolepis* Ehlers, 1908	Reported by Simboura and Zenetos (2005).
*Polygordius* Schneider, 1868	Reported by NCMR (1995), NCMR (1997), NCMR (1998).
*Tomopteris* Eschscholtz, 1825	Reported by Ramfos et al. (2005), Ramfos et al. (2006), Isari et al. (2010).
*Paraleiocapitella* Thomassin, 1970	*Paraleiocapitella* is considered a synonym of *Notomastus* (Ewing, 1982), although Green (2002) argues that *Paraleiocapitella* should be differentiated from *Notomastus* based on chaetal characters. *Paraleiocapitella* is monotypic, thus if the generic identification of the Greek specimens (Nicolaidou and Papadopoulou 1989, Eleftheriou et al. 1990, Simboura 1996) is correct, they probably belong to *Notomastus mossambicus* (Thomassin, 1970), an alien species first reported from the Mediterranean by Çınar (2005).

**Table 4.** Species originally described from Greek waters (i.e. the Exclusive Economic Zone as defined by marineregions.org).

**Family**	**Described as**	**Currently accepted as**	**Type locality**
Acrocirridae	*Flabelligella mediterranea* Kolmer, 1985	*Flabelligena mediterranea* (Kolmer, 1985)	Hellenic Trench, Matapan Deep 4690 m, mud, 35°49.8'N, 22°20.7'E
Ampharetidae	*Uschakovius enigmaticus* Laubier, 1973	*Uschakovius enigmaticus* Laubier, 1973	Hellenic Trench, Matapan Deep 3174 m, 36°01.8'N, 22°24.6'E
Amphinomidae	*Amphinome savigny* Brullé, 1832	*Hermodice carunculata* (Pallas, 1776)	Methana, Saronikos Gulf, Greece
Capitellidae	*Peresiella clymenoides* Harmelin, 1968	*Peresiella clymenoides* Harmelin, 1968	Mediterranean Sea (Marseilles, Crete and Santorini)
Fabriciidae	*Pseudofabriciola longipyga* Fitzhugh, Giangrande & Simboura, 1994	*Pseudofabriciola longipyga* Fitzhugh, Giangrande & Simboura, 1994	Geras Gulf, Lesvos Island, 21 m, muddy sand
Lacydoniidae	*Lacydonia laureci* Laubier, 1975	*Lacydonia laureci* Laubier, 1975	Hellenic Trench, Matapan Deep 4690 m, mud, 35°49.8'N,22°20.7'E
Asteriomyzostomidae	*Myzostoma asteriae* Marenzeller, 1895	*Asteriomyzostomum asteriae* (Marenzeller, 1895)	Santorini, Kythira and Samos
Onuphidae	*Paradiopatra calliopae* Arvanitidis & Koukouras, 1997	*Paradiopatra calliopae* Arvanitidis & Koukouras, 1997	Aegean Sea, off Mount Athos coast, 80-300 m, silty sand to silt, 40°20'25"N,24°18'05"E
Orbiniidae	*Scoloplos chevalieri candiensis* Harmelin, 1969	*Scoloplos chevalieri candiensis* Harmelin, 1969	Kaloi Limenes, Crete, 7-11 m, *Halophila stipulacea* and *Cymodocea nodosa* meadows
Sabellariidae	*Phalacrostemma cidariophilum* Marenzeller, 1895	*Phalacrostemma cidariophilum* Marenzeller, 1895	Several locations near Crete and Kythira
Serpulidae	*Serpula graeca* Brullé, 1832	*Protula intestinum* (Lamarck, 1818)	Methana, Saronikos Gulf, Greece
Serpulidae	Spirorbis (Janua) gnomonicus Bailey, 1969	*Janua heterostropha* (Montagu, 1803)	Emborios Cave, Chios, Aegean Sea, 8-10 m
Serpulidae	Spirorbis (Janua) parvulus Bailey, 1969	*Neodexiospira pseudocorrugata* (Bush, 1905)	Emborios Cave, Chios, Aegean Sea
Serpulidae	*Vermilia agglutinata* Marenzeller, 1893	*Semivermilia agglutinata* (Marenzeller, 1893)	Mediterranean Sea (Libya and Greece)
Sigalionidae	*Pholoe dorsipapillata* Marenzeller, 1893	*Pholoides dorsipapillatus* (Marenzeller, 1893)	Mediterranean Sea (Greece and Egypt)
Syllidae	*Myrianida rodosensis* Çınar, 2015	*Myrianida rodosensis* Çınar, 2015	Rhodes, Faliraki port,1 m, photophilic algae, 36°20.13'N, 28°12.52'E,
Syllidae	*Streptosyllis nunezi* Faulwetter et al., 2008	*Streptosyllis nunezi* Faulwetter et al., 2008	Pachia Ammos, Crete, 1–5 m, coarse sand, 35°06'43''N, 25°48'34''E
Terebellidae	*Amphitritides kuehlmanni* Arvanitidis & Koukouras, 1995	*Amphitritides kuehlmanni* Arvanitidis & Koukouras, 1995	Evoikos Gulf, coast of Loukissia, 5 m, on rocks with *Cystoseira*, 38°28'55"N, 23°29'10"E

**Table 5.** Species not found in Greece since 1993 (chosen as a cut-off date, as thorough investigations of the Greek polychaete fauna (e.g. Arvanitidis 1994, Simboura 1996) followed afterwards. Except for museum specimens, dates are publication dates, not collection dates.

**Year of last report**	**Species**
1887	*Spinther arcticus*
1895	*Asteriomyzostomum asteriae*
1902	*Aglaophamus malmgreni, Leocrates atlanticus*
1939	*Krohnia lepidota, Lopadorrhynchus appendiculatus, Lopadorrhynchus brevis, Lopadorrhynchus nationalis, Lopadorrhynchus uncinatus, Rhynchonereella moebii, Torrea candida, Travisiopsis lanceolata, Vanadis crystallina, Vanadis formosa*
1959	*Aphrodita perarmata, Phyllochaetopterus gracilis*
1961	*Alkmaria romijni*
1962	*Alitta virens, Bushiella (Jugaria) granulata*
1964	*Phalacrostemma cidariophilum*
1969	*Naineris quadraticeps, Vinearia endoumensis*
1971	*Ninoe nigripes, Paradiopatra lepta*
1973	*Bathyfauvelia affinis, Uschakovius enigmaticus*
1975	*Lacydonia laureci*
1977	*Neoamphitrite edwardsi*
1982	*Hyboscolex longiseta, Odontosyllis dugesiana*
1983	*Aonidella cirrobranchiata, Epigamia alexandri, Eulalia bilineata, Eunice floridana, Eunice roussaei, Euphrosine myrtosa, Manayunkia aestuarina, Pareurythoe borealis*
1984	*Anoplosyllis edentula, Ophryotrocha labronica, Parapionosyllis elegans*
1985	*Prionospio decipiens*
1986	*Antinoe aequiseta, Aricidea (Strelzovia) quadrilobata, Lumbrinerides acuta, Maldane capensis, Maupasia coeca, Phylo norvegica, Scolelepis gilchristi, Scoloplos haasi, Spiophanes duplex*
1987	*Aricidea (Aricidea) longicirrata, Goniadella gracilis, Johnstonia clymenoides, Metavermilia acanthophora, Opisthodonta longocirrata, Streptosyllis bidentata, Syllis nigricirris*
1988	*Lindrilus flavocapitatus*
1989	*Desdemona ornata, Eunice norvegica, Gattyana cirrhosa, Proclea graffii, Protocirrineris chrysoderma, Sabellaria eupomatoides*
1990	*Neoamphitrite groenlandica, Oxydromus agilis, Praxillura longissima, Syllides longocirratus*
1991	*Arabella geniculata, Autolytus rubrovittatus, Boccardiella cf. ligerica, Claparedepelogenia inclusa, Malacoceros tetracerus, Neoamphitrite affinis, Neoleanira tetragona, Nephtys ciliata, Notomastus lineatus, Paranaitis kosteriensis, Phylo kupfferi, Protodorvillea atlantica, Scoletoma tetraura*
1992	*Ampharete grubei, Bathyvermilia langerhansi, Eunice schizobranchia, Eunoe assimilis, Nicomache trispinata, Polyodontes frons*
1993	*Diplocirrus hirsutus, Levinsenia multibranchiata, Pelagobia longicirrata, Perinereis tenuisetis, Pseudomystides limbata nigrolineata*
