# Supplementary material for: Polychaetes of Greece: an updated and annotated checklist
Source: Biodivers Data J. 2017 Dec 22;(5):e20997. doi: 10.3897/BDJ.5.e20997 (PMC5769717; doi:10.3897/BDJ.5.e20997)
Supplement: Supplementary material 1 — List of unpublished / online resources [file bdj-05-e20997-s001.pdf]

**List of unpublished / grey literature / online sources used for the compilation of the Checklist of Polychaetes in Greece.**


Unpublished data from meiobenthic studies of sandy beaches (Papageorgiou N, Arvanitidis C, Eleftheriou A (2006) Multicausal environmental severity: A flexible framework for microtidal sandy beaches and the role of polychaetes as an indicator taxon. Estuarine, Coastal and Shelf Science 70: 643–653. doi: 10.1016/j.ecss.2005.11.033

GBIF occurrence data for museum specimens of polychaetes from Greece. Downloaded from http://gbif.org (9th September 2016); http://doi.org/10.15468/dl.z8fcac

SESAM – Online collections of the Senckenberg museum (http://sesam.senckenberg.de/page/index.htm)

HCMR (Hellenic Centre for Marine Research), 2012-2015. The monitoring network for the ecological status quality of the transitional and coastal waters of Greece according to article 8 of the WFD 2000/60/EC. HCMR —Ministry of Environment. N. Simboura & P. Panayotidis, (Eds). Annual Technical Reports, (in Greek). These data can also be accessed through the European SeaDataNet discovery and access system (http://www.seadatanet.org/Data-Access) or through the HCMR oceanographic database (http://hnodc.hcmr.gr/)

HCMR, 2003. Spatial planning for the development of marine aquaculture in the area of Thesprotia. Technical report, D. Georgopoulos (Ed.). (In Greek).

HCMR, 2004. Survey of the coastal ecosystem of the Northern Rhodos island (1996 2003). Dodekanesse Perfecture. Technical report, G.A. Hatiris (Ed.). pp: 1342. (In Greek).

HCMR, 2004. Investigation of oceanographic parameters of spatially planned marine aquaculture areas in Echinades islands. Technical report, Dr. P. Panayotidis & E. Zaggana (Eds). (In Greek).

FATE project, 2005. Transfer and Fate of Harmful Algal Bloom (HAB) toxins in European Marine Waters. (1 January 2002–31 December 2004). E. Graneli (coord.) Final Scientific and Management Report.Contract EVK3 CT0100055, part of the EC EUROHAB cluster.

HCMR, 2006. Oceanographic study of Laganas Bay (Zakynthos island), Technical report. P. Panayotidis (Ed.). (In Greek).

HCMR, 2007. Recording of the marine environmental quality in the area of treated sewage effluents of the biological treatment plants of Marpissa and Paroikia in Paros island. Technical report, E. Krassakopoulou (Ed.). (In Greek).

HCMR, 2008. Oceanographic study of the Aliveri Bay in relation to AGET Herakles cement industry. Technical report. Dr. A. Pavlidou (Ed.). (In Greek).

HCMR, 2008-2009. Recording of the environmental conditions in the area of Nies Bay (Pagassitikos Gulf). Technical report. Dr. M.A. Pancucci Ed. (In Greek).

HCMR, 2009. Preliminary study of the nonindigenous fauna of the marine ecosystem of SE Aegean Sea. Technical report. (M. CorsiniFoka & M.A. Pancucci Eds), 74 p. (In Greek).

HCMR (2007-2011). Study of the short-term effects from the accident of the cruise ship 'SEA DIAMOND' in Athinios Bay, Thera island. Ministry of Merchantile Marine. Technical report. Dr. J. Hatzianestis (Ed.). (In Greek).

HCMR, 2009-2010. Study of the environmental impact from the high salinity water discharged from the cooling system of the thermoelectric plant of the Chalyvourgiki AE steel industry. Technical report. E. Strogyloudi (Ed.). (In Greek).

HCMR, 2010-2011. Environmental monitoring of the marine dumping area (Saronikos Gulf) of the spoil dredges from Kifissos river lower bed. Technical reports, Dr. V. Kapsimalis (Ed.). (In Greek).

HCMR, 2010-2013. Recording of the environmental conditions in Aliveri Bay in relation to the

functioning of the desalination plant in AGET Herakles cement industry. Technical report, Dr. A. Pavlidou (Ed.). (In Greek).

HCMR 2006-2010. Monitoring of the quality of the marine environment of Messiniakos Gulf and the western coasts of Messinia area. Technical reports, Dr. A. Pavlidou (Ed.). (In Greek).

HCMR (2011-2015). Monitoring of marine environmental quality in the area of the sewage outfall of Thriasio treatment plant in Elefsis Gulf. Technical Reports. S. Zervoudaki (Ed). (In Greek).

CoCoNet project FP7, 2014. Towards COast to COast NETworks of marine protected areas (from the shore to the high and deep sea), coupled with sea-based wind energy potential. Report on the soft bottom benthic communities of the study area. Assessment of environmental status.

Perseus, FP7 project, 2012-2015. Policy-oriented marine Environmental Research for the Southern European Seas.The Benthic communities Condition in AEGEX experiment (North Aegean). WP1 Technical report. EC FP7 PERSEUS Project (Grant. Agr. 287600).

H.C.M.R., 1998-2015. Study of the evironmental impact of dumping coarse metalliferous waste in the area off Larymna. N. Simboura (Ed.) & K. Tsangaris (Ed.), Technical Reports, (In Greek).

H.C.M.R., 1999-2012. Monitoring of the Saronikos Gulf ecosystem affected by the Psittalia Sea outfalls. I. SiokouFragou & E. Christou & E. Krassakopoulou & S. Zervoudaki (Eds.), Annual Technical Reports. (In Greek).
